# Identification of a Candidate Proteomic Signature to Discriminate Multipotent and Non-Multipotent Stromal Cells

**DOI:** 10.1371/journal.pone.0038954

**Published:** 2012-06-13

**Authors:** Michael Rosu-Myles, Yi-Min She, Joel Fair, Gauri Muradia, Jelica Mehic, Pablo Menendez, Shiv S. Prasad, Terry D. Cyr

**Affiliations:** 1 Centre for Vaccine Evaluation, Biologics and Genetic Therapies Directorate, Health Products and Food Branch, Health Canada, Ottawa, Canada; 2 Department of Biochemistry, Microbiology and Immunology, University of Ottawa, Ottawa, Canada; 3 Pfizer-University of Granada-Andalusian Government Centre for Genomics and Oncological Research, Granada, Spain; University of Medicine and Dentistry of New Jersey, United States of America

## Abstract

Bone marrow stromal cell cultures contain multipotent cells that may have therapeutic utility for tissue restoration; however, the identity of the cell that maintains this function remains poorly characterized. We have utilized a unique model of murine bone marrow stroma in combination with liquid chromatography mass spectrometry to compare the nuclear, cytoplasmic and membrane associated proteomes of multipotent (MSC) (CD105+) and non-multipotent (CD105−) stromal cells. Among the 25 most reliably identified proteins, 10 were verified by both real-time PCR and Western Blot to be highly enriched, in CD105+ cells and were members of distinct biological pathways and functional networks. Five of these proteins were also identified as potentially expressed in human MSC derived from both standard and serum free human stromal cultures. The quantitative amount of each protein identified in human stromal cells was only minimally affected by media conditions but varied highly between bone marrow donors. This study provides further evidence of heterogeneity among cultured bone marrow stromal cells and identifies potential candidate proteins that may prove useful for identifying and quantifying both murine and human MSC *in vitro*.

## Introduction

Bone marrow contains several different cell types including the precursors of blood, bone, fat and connective tissue. In the early seventies, it was shown that a monolayer of adherent fibroblast-like cells could be propagated *in vitro* by culturing whole bone marrow cells for several weeks in serum containing media [1]. These cells were found to support the growth of hematopoietic progenitors and could differentiate into fat (adipocytes), bone (osteocytes) and cartilage (chondrocytes) precursors both *in vitro* and *in vivo* [2], [3]. Subsequently, they have been labelled as multipotent stromal cells (MSC) or mesenchymal stem cells. More recently, MSC cultures have been ascribed with other clinically relevant properties, such as immune suppression [4]–[6], and a capacity to enhance tissue repair [5]–[12]. Hence, MSC cultures have immense therapeutic potential and are currently being tested in clinical trials for the treatment of cartilage degeneration, myocardial infarction, diabetes, graft versus host disease and neurodegenerative disorders, among others [8]–[10], [13]–[17].

There have been several lines of evidence suggesting that stromal cultures represent a heterogeneous population of cells containing MSC. Differences have been identified in expansion potential [18], differentiation capacity [19]–[22] and transcriptomic and proteomic profiles [23], [24]. In all, the body of evidence within the literature suggests that the frequency of MSC among stromal cultures varies based on donor, media conditions and culture age. Despite this, markers that can discriminate MSC from non-multipotent stroma remain elusive. The identification of such markers may allow variation to be characterized between cultures, providing a useful tool in both basic research and clinical settings.

Previous research in our lab has demonstrated that C57BL/6 mouse bone marrow provides a unique stromal cell culture model that can be used to discriminate multipotent and non-multipotent stroma based on the expression of CD105 [20], [23]. In the current work, CD105 expressing (CD105+) and non-expressing (CD105−) stromal cells were purified from C57BL/6 stromal cultures and analyzed by liquid chromatography mass spectrometry (LC-MS/MS) to compare the proteome of MSC and non-multipotent stroma. Subfractionation techniques allowed the identification of several hundred nuclear, cytoplasmic and membrane derived proteins that were unique to either CD105+ or CD105− cells and contributed to distinct canonical pathways, as demonstrated by Ingenuity Pathway Analysis (IPA). Specific expression in CD105+ cells of 10 different proteins was verified by Western Blotting and real time polymerase chain reaction. Screening of human stromal cultures determined that five of the 10 proteins uniquely expressed in murine CD105+ cells were also detectable, at varying levels, in human bone marrow stromal cultures shown to contain MSC. This work provides a proteomic signature that is specific to MSC and a list of potential candidate *in vitro* markers for these therapeutically relevant cells.

## Materials and Methods

### Bone Marrow Stromal Cultures

This study was approved by the Health Canada Animal Care Committee and all housing and treatment of animals was carried out according to the approved protocol. Murine stromal cultures were initiated as previously described [23]. Briefly, bone marrow (BM) was flushed from femur, tibia and iliac crest of 8–12 week old female C57BL/6J mice (Jackson Laboratories, Bar Harbour, ME) and was seeded at 4.0×10^6^ white blood cells (WBC) per millilitre (mL) of Mesencult MSC Basal Medium containing murine MSC stimulatory supplements, referred to here-in as Mesencult Complete Medium (StemCell Technologies, Vancouver, BC). After 14 to 21 days, cells were harvested with Trypsin-EDTA (StemCell Technologies) and endothelial and hematopoietic cells were removed using 2 rounds of immunomagnetic purification with a custom EasySep cocktail (StemCell Technologies). Human bone marrow was purchased from Lonza Walkersville (Lonza, Walkersville, MD), an establishment registered under the FDA for the processing of human cells, tissue and cellular and tissue based products in accordance with the US Code of Federal Regulations (21 CFR Par 1271). Human tissues provided by Lonza are obtained from various tissue suppliers and recovery agencies according to Institutional Review Board approved protocols and informed consent that allow the use of obtained tissues for general research purposes. Human stromal cell cultures were initiated either by plating 1×10^6^ cells per mL in low glucose Dulbecco's Modified Eagles Medium (DMEM) (Invitrogen/GIBCO BRL, Burlington, ON) with 15% Fetal Bovine Serum (FBS) qualified for human MSC (HyClone-Thermo-Fisher, Nepean, ON) [Serum Containing (SC) media] or in Serum and Animal Component Free media provided by Stem Cell Technologies, herein referred to as Serum Free (SF) media.

### Flow Cytometry and Cell Sorting

Stromal cells were trypsinized, filtered through a 70 µm cell strainer (BD Bioscience, San Diego, CA) and resuspended in PBS/2%FBS at 1×10^3^ cells/µL. Cells were simultaneously stained with fluorochrome-conjugated monoclonal antibodies (mAb) to human CD105-Allophycocyanin (APC) (SN6); CD34-Phycoerythrin-Cy7 (PE-Cy7) (4H11); CD45-Fluorescein Iso-Thiocyanate (FITC) (H130); CD90-PE-Cy5.5 (5E10); Mdr-1-PE (U1C2) (eBioscience, San Diego, CA) and CD73-PE (AD2) (BD Bioscience). For Fluorescence Activated Cell Sorting (FACS), murine stromal cells were stained with anti-mouse CD105-PE (MJ7/18) (eBioscience). Flow cytometric analysis was completed on a minimum of 30 000 viable cells using an LSR II instrument (BD Bioscience) and the data were analyzed using FLOWJO™ software (TreeStar Inc., Ashland, OR). FACS was completed on a MoFlo™ instrument (Beckman Coulter, Mississauga, ON).

### Multipotent Differentiation Cultures

Differentiation of stromal cells into adipocytes, osteocytes and chondrocytes was completed using the Human MSC Functional Identification Kit from R&D Systems (R&D Systems, Minneapolis, MN). Briefly, to initiate adipocyte and osteocyte formation, stromal cells were cultured in either SC or SF media in 24-well plates using 2.1×10^4^ cells/cm^2^ and 4.2×10^3^ cells/cm^2^ cells, respectively. Media containing supplements to allow the differentiation of adipocytes or osteocytes was added when cells reached 100% or 50% confluence, respectively. Media was changed every 3–4 days over a 10–28 day period. For chondrogenic differentiation, 1.25×10^5^ of cultured stromal cells were grown in 15 mL polypropylene conical tubes with DMEM/F12 media containing chondrogenic supplements. Media was changed every 3 days for 17–21 days. Adherent cells and pellets were fixed in 4% paraformaldehyde and either stained directly (adipocytes/osteocytes) or cryosectioned prior to staining (chondrocytes). Sections of 8–10 µm were placed on charged glass slides (VWR, Mississauga, ON). For chemical detection of adipocytes, fixed cells were treated with 60% isopropanol for 10 minutes and were stained with Oil Red O for 5 minutes (Sigma-Aldrich, St. Louis, MO). Osteocyte identification was accomplished by staining fixed cells with 0.2% Alizarin Red Stain at pH 6.36–6.4 (Baker) for 60 minutes. The cryosectioned pellets acquired from chondrocyte differentiation cultures were stained in Alcian blue stain, pH 1.0, for 15 minutes. Stained cells were washed thoroughly with distilled water and visualized under a light microscope (Zeiss, Toronto, ON) equipped with an Axiom camera.

### Cellular Subfractionation and Protein Purification

For cellular subfractionation, 5×10^7^ CD105+ and CD105− cells were harvested using enzyme-free cell dissociation buffer (Invitrogen) and centrifuged at 700 g for 5 minutes at +4°C. The cells were washed and resuspended in 1 ml of homogenization buffer mix from BioVision's Membrane Protein Extraction Kit (BioVision, San Francisco, CA). The cells were lysed in an ice cold Dounce homogenizer and centrifuged at 700 g for 10 minutes at +4°C. Pellets consisting of intact nuclei were kept for nuclear extraction while supernatants were used to purify cytosolic and membrane protein fractions according to the manufacturer's instructions (BioVision). The nuclear pellet was dissolved in 250 µl ice-cold NEB mix from BioVision's Nuclear/Cytosol Fractionation Kit, incubated on ice for 10 minutes and vortexed for 15 seconds. The nuclear proteins were collected by centrifugation at 16,000 g for 10 minutes. Proteins from each subfraction were quantified using a BCA Protein Assay Kit and 200 µg of each was utilized for mass spectrometry.

### LC MS/MS and Proteomic Data Analysis

Proteins were digested by sequencing-grade trypsin in 25 mM NH_4_HCO_3_ at 37°C overnight, following reduction with 10 mM dithiothreitol, alkylation with 55 mM iodoacetamide and subsequently dialysis to remove these chemicals. Further separation of the protein extracts was carried out on 10% SDS-PAGE gel (Bio-Rad Laboratories, CA), and the Coomassie-stained bands were excised and digested in-gel using a standard method described elsewhere [23].

Online LC MS/MS analysis was performed on a Nano-Acquity ultra-performance liquid chromatography system (UPLC, Waters, Milford, MA) coupled to a 7-tesla hybrid linear ion trap Fourier transform ion cyclotron resonance mass spectrometer (LTQ-FT ICR, Thermo Fisher, San Jose, CA). The peptides were trapped by a RP Symmetry C18 column (180 µm i.d.×20 mm length, 5 µm; Waters) at 5 µL/min, and subsequently separated on a C18 analytical column (100 µm i.d.×100 mm, 1.7 µm, BEH 130; Waters) at 500 nl/min. Peptides were eluted using mobile phases consisting of solvent A (0.1% formic acid) and solvent B (97.9% acetonitrile/0.1% formic acid/2% water). NanoUPLC separation was achieved by a linear gradient from 5% to 45%, and then 85% of solvent B at a duration time of either 90-min for the tryptic digest of protein gel isolates, or 4 hours for the digest of proteins isolated directly from subcellular fractions.

Mass spectrometric data were acquired by the data-dependent mode following a full FT-MS survey scan over a mass range of m/z 300–2000. Protein identification was achieved by searching against SwissProt-UniProt (updated on December 1^st^, 2011, 533049 sequences) and NCBI (16338050 sequences) decoy databases using an in-house Mascot Server (version 2.3.2, Matrix Science, London, UK) with a false discovery rate less than 5%. The Mascot search parameter settings allowed trypsin digestion for maximum 2 missed cleavage sites, and carbamidomethylation of cysteine as a fixed modification. Deamidation of Asn and Gln, oxidation of Met, and N-terminal pyroglutamation of Gln were considered as variable modifications. Mass tolerances were set up to 10ppm for the FT MS ions, and 1 Da for the ion trap MS/MS fragment ions. The results were then combined and analyzed by Scaffold (version 3.1.4.1, Proteome software Inc. Portland, OR) to allow side-by-side comparison of proteins identified in subcelluar fractions. For Scaffold analysis, parameters were based on the identification of at least 1 peptide with an ion score greater than 20. This resulted in 77079 spectra with a 0.1% FDR that were assigned to 1254 proteins with an FDR of 0.8%.

### Real Time Quantitative RT-PCR

Total RNA was purified from 2×10^6^ CD105+ and CD105− cells using the RNeasy Plus Mini Kit (Qiagen, Toronto, ON) according to the manufacturer's protocol. Extracts were treated with DNase (Applied Biosystems, Streetsville, ON) and quantified using a Nanodrop 1000 spectrophotometer (Fisher Scientific). One microgram RNA was used to synthesize cDNA using Superscript III First Strand Synthesis System for RT-PCR (Invitrogen/Gibco). Real time PCR was completed using the Power SYBR Green PCR Master Mix and the 7500 FAST Real-Time PCR System (Applied Biosystems). PCR results were normalized to actin expression and the delta-delta CT method was used for determining fold changes. For a complete list of utilized primers see Table S1.

### Western Blotting

Whole cell protein extracts (5–40 ug) were loaded onto 8–12% acrylamide gels, separated and transferred to PVDF membranes (Millipore, Etobicoke, ON) for 1 hour. After overnight blocking at 4°C with 1% Western Blocking Reagent (Roche, Mississauga, ON) in TBS + 0.1% Tween 20, blots were incubated with primary antibodies (Table S2) as per manufacturer's instructions. Primary antibody was visualized using either anti-rabbit or anti-mouse IgG Horseradish Peroxidase (HRP) conjugated antibody (1∶2000) (GE Healthcare, Mississauga, ON), anti-Goat IgG-HRP (1∶10 000) (Santa Cruz Biotechnology) or anti-Sheep IgG HRP (1∶200 000) (Jackson Labs) for 1 hour at room temperature. After washing, the presence of antibody was detected using SuperSignal West Dura Extended Duration Substrate. Protein loading was normalized using anti-actin (1∶50 000) or anti-vinculin (1∶2000) (Sigma)

## Results

### Purification of murine stromal cells based on CD105 expression allows proteomic comparison of MSC and non-multipotent stroma

**Figure 1:**
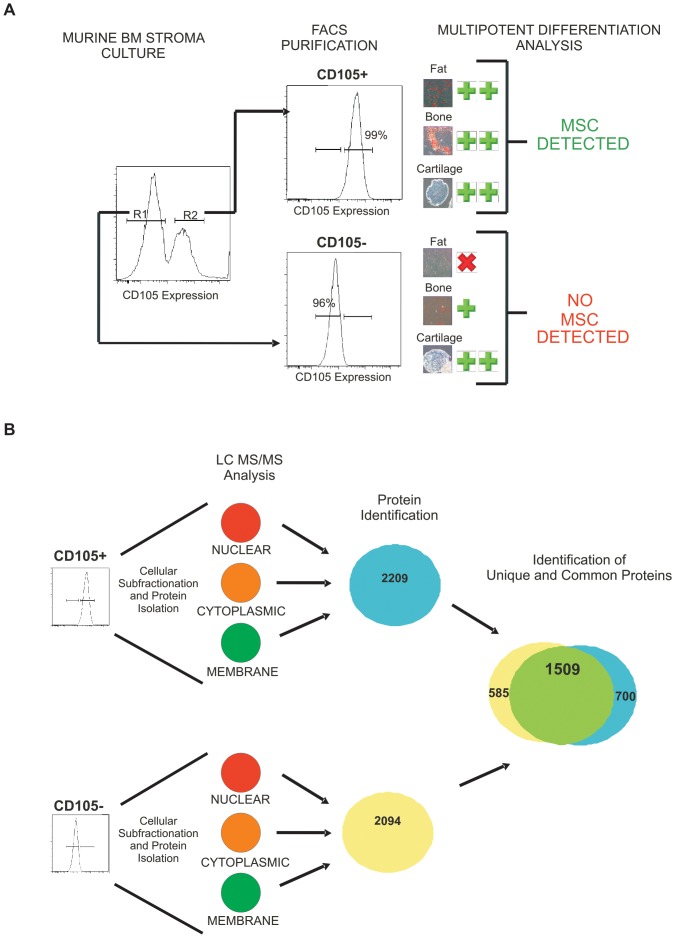
Purification of murine stroma based on CD105 expression allows the identification of proteins that are unique to multipotent and non-multipotent stroma. (A) CD105 expression demarcates MSC and non-multipotent stromal cells in C57BL/6 bone marrow derived cultures. CD105− (R1) and CD105+ (R2) stromal cells were purified by FACS and cultured under conditions that support adipocyte, osteocyte and chondrocyte differentiation. (n = 3) (B) Schematic of method used to compare the proteome of multipotent and non-multipotent stroma. CD105+ and CD105− stroma were isolated from 3 separate cultures. Proteins were isolated from the nuclear, cytoplasmic and membrane fractions and analyzed by LC MS/MS. Proteins identified in each of the 3 experiments were pooled to generate data sets representative of MSC (blue) or non-multipotent stroma (yellow). Data from the two populations was generated from both the NLM and Swiss Prot data bases and compared using Microsoft Access™ to generate lists of both unique and common proteins.

The identification of distinct populations of multipotent and non-multipotent stromal cells in C57BL/6 mouse bone marrow cultures that could be distinguished based on CD105 expression (20) provided a unique opportunity to identify specific markers of MSC. As a first step in accomplishing this goal, we set out to utilize the C57BL/6 stromal culture model to generate a list of proteins that are specifically expressed in CD105+ stromal cells. As mass spectrometry has proven a reliable method for analyzing cellular proteins [23], [25], [26], we utilized LC MS/MS to compare the proteome of FACS purified CD105+ and CD105− stroma. In three replicate experiments, stromal cell populations were isolated to a purity of ≥95% and tested in differentiation culture assays to ensure that the multipotent potential of CD105+ and CD105− fractions was maintained (Figure 1A). As observed in our previous studies [20], only CD105+ cells demonstrated the capacity to differentiate into adipocytes, osteocytes and chondrocytes.

To increase the depth of coverage for LC MS/MS analysis, and reduce masking from high abundance proteins, FACS purified CD105+ and CD105− cells were subfractionated into nuclear, cytosolic and membrane components (Figure 1B). Proteins were isolated from each of these subfractions individually and nuclear and cytosolic proteins were analyzed by LC MS/MS directly in each of the three replicate experiments. CD105+ and CD105− cell derived membrane proteins were divided in half and further purified to remove detergents using one of two methods: PAGE separation and Filter Assisted Sample Preparation (FASP) [27]. While more proteins were identified during LC MS/MS analysis of PAGE separated membrane proteins, some proteins could only be consistently identified by FASP (data not shown). Thus, the results obtained from the purification of membrane proteins using both methods were combined to determine membrane proteomic profiles for CD105+ and CD105− stroma.

A list of proteins expressed by CD105+ or CD105− cells was generated from datasets obtained by LC MS/MS analyses of nuclear, cytoplasmic and membrane subfractions through MASCOT using P values of >0.05 and a 5% false discovery rate. Peptides were assigned using both the mouse NCBI and SwissProt-UniProt databases and those with MASCOT scores less than 20 were discarded. Lists were further refined using Scaffold analysis to compare the nuclear, cytosolic and membrane proteins identified in replicate experiments; any proteins that were not identified in each experiment were omitted. Proteins determined from nuclear, cytosolic and membrane subfractions were then compiled to generate a list of 2209 and 2094 proteins expressed in CD105+ and CD105− cells, respectively. A database comparison of these lists found 1509 proteins (70%) common to both cell types (Figure 1B). Proteins were also identified that were characteristic to either CD105+ or CD105− cell extracts. Specifically, 700 proteins were detected only in CD105+ cells, while 585 were detected in CD105− cells, in each of the three biological replicate cultures analyzed. Thus, through extensive protein fractionation, LC MS/MS analysis and data comparison techniques, we have identified a list of proteins that may be expressed at different levels in murine MSC and non-multipotent stroma.

### Proteins characteristic to either CD105+ or CD105− stroma contribute to divergent canonical pathways

**Figure 2:**
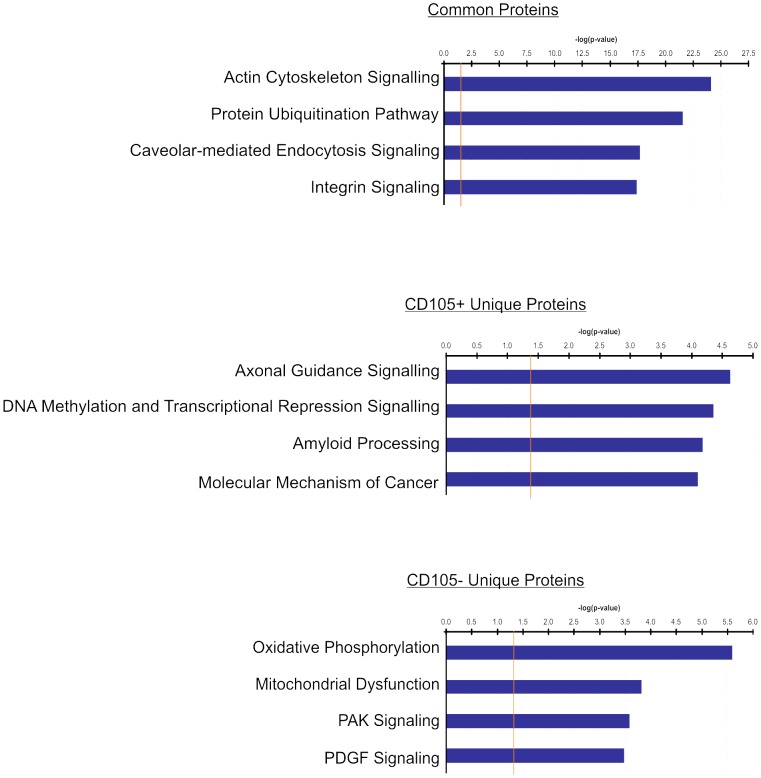
Proteins uniquely expressed in multipotent and non-multipotent stroma are involved in distinct canonical pathways. Lists of proteins that were either common or unique to CD105+ and CD105− stroma were analyzed using Ingenuity Pathway Analysis software. The orange line signifies the cut-off P value used to assess the significance of each pathway. The top 4 canonical pathways identified in each group are reported.

Currently, little is known about the biological pathways that are activated in cultured stromal cells. To determine whether proteins that were identified as common or unique to MSC and non-multipotent stroma were enriched within specific canonical pathways, the sets of proteins identified by LC MS/MS analysis of CD105+ and CD105− cells were subjected to Ingenuity Pathway Analysis (IPA). The top four most significant canonical pathways identified from the common and unique protein lists are shown in Figure 2 (p≤0.05). Cytoskeletal, cell maintenance and integrin signalling pathways were identified as common to both cell types. IPA determined that the LC MS/MS generated CD105+ and CD105− unique protein lists were enriched for proteins involved in distinct canonical pathways. Specifically, proteins identified among CD105− cell extracts were involved in oxidative phosphorylation and mitochondrial dysfunction pathways while the top canonical pathways among proteins characterized in CD105+ cells were axonal guidance signalling, DNA methylation and transcriptional repression signalling. The gene symbols corresponding to each of the proteins associated with these four canonical pathways are listed in Table 1. Overall, IPA provides evidence that proteins identified through LC MS/MS analysis of CD105+ and CD105− cells comprise distinct protein sets representing two functionally distinct stromal cell populations.

**Table 1:** List of proteins identified by LC MS/MS in CD105+ cells that participate in specific canonical pathways as determined by IPA analysis.

**Axonal Guidance Signaling**	ABLIM3; ADAM29; AKT1; AKT2; CDK5; CRK; EPHB6; GLI2PAK1; GNA12; GNAL; GNAO1; GNAT2; MYL6B; NGEF; NRP1; SRGAP1; PAK2; PRKCA; PRKACB; PRKAR1A; PRKAR2A; PLXNA1; RAC3; RGS3; ROBO1; ROCK1; RRAS2; TUBA1C; TUBA4A
**DNA Methylation and Transcriptional Repression Signaling**	CHD3; CHD4; MTA1; RBBP7; MTA2; ARID4B
**Amyloid Processing**	AKT1; AKT2; CAPN6; CDK5; CSNK2A2; CSNK2B; PRKACB; PRKAR2A; PRKAR1A
**Molecular Mechanisms of Cancer**	AKT1; AKT2; ARHGEF1; CASP6; CDK2; CDK4; CDK6; CRK; DAXX; FOS; GNA12; GNAO1; GNAL; GNAT2; PAK1; PAK2; PRKACB; PRKAR1A; PRKAR2A; PRKCA; RAC3 RAPGEF1; RASGRF2; RHOC; RHOG; RRAS2

Proteins detected only in CD105+ cell extracts were analyzed by IPA software to determine their potential role in canonical signalling pathways. Proteins identified to participate in the top four canonical pathways are listed in alphabetical order.

### Real-time RT-PCR and Western Blot analysis confirm LC MS/MS identified proteins as candidate markers of murine MSC

In order to further assess the list of proteins identified in CD105+ cells as potential markers of MSC, we set out to quantitatively compare their expression at the mRNA and protein level. To narrow the focus of the study to a more manageable number of proteins, we chose to focus on the top 25 CD105+ cell specific proteins as ranked by Scaffold analysis. These proteins are listed in Table 2, in the order of their Scaffold ranking, along with the subcellular fraction in which they were associated. The number of unique peptides identified and percent protein coverage from a single representative experiment are also given. The sequences of peptides identified by LC MS/MS and used for protein identification, in this representative experiment, are shown in Table S3 along with their ion scores. Among the 25 proteins listed, 10 proteins were chosen for quantitative verification based on the commercial availability of reliable antibodies. These included the membrane associated proteins CD248, Thrombospondin-1 (Thbs1), Neuropilin-1 (Nrp1) and Multi-drug Resistant Protein-1 (Mdr-1; P-glycoprotein) as well as nuclear and cytosolic proteins Prostacyclin synthase (Ptgis), Adenosine kinase (Adk), Growth arrest specific protein-2 (Gas2), Epoxide hydrolase-1 (Ephx1), Fibulin-2 and High mobility group box-1 (Hmgb1). In addition, based on their known roles in cell signalling, we chose to investigate the expression levels of two additional membrane proteins that were identified with lower Scaffold ranks: Oncostatin M receptor-1-beta (Osmr1) and Ephrin receptor B3 (EphB3).

**Table 2:** List of 25 CD105+ cell unique proteins with the highest Scaffold ranking.

Protein name	Abbr.	Access. number	Unique peptides (coverage) Nuclear Cytosol Membrane		
High mobility group box 1	Hmgb1	P63158	19(54%)	0	0
Chondroitin sulfate proteoglycan 4	Cspg4	Q8VHY0	0	0	63(34%)
Type VI collagen alpha 3	Col6a3	D3YWD1	0	0	38(35%)
Prostacyclin synthase	Ptgis	O35074	7(20%)	2(7%)	19(47%)
Serum deprivation-response protein	Sdpr	Q63918	7(22%)	5(18%)	0
FK506 binding protein 3	Fkbp3	Q3UBU9	7(33%)	0	0
Adenosine kinase	Adk	P55264	9(36%)	9(36%)	0
Biglycan	Bgn	P28653	0	0	11(35%)
Endosialin-CD248	CD248	Q91V98	0	0	3(3%)
Collagen alpha-1(VI) chain	Col6a1	Q04857	0	0	18(20%)
Growth arrest-specific protein 2	Gas2	P11862	0	0	16(64%)
Topoisomerase (DNA) II beta	Top2b	Q64511	9(7%)	0	0
Flightless I homolog	Flii	Q9JJ28	0	5(5%)	0
Collagen alpha-2(VI) chain	Col6a2	Q02788	0	0	11(13%)
Yap1 protein	Yap1	P46938	6(24%)	0	0
Epoxide hydrolase 1	Ephx1	Q9D379	6(14%)	0	28(56%)
Isoform 6 of Dynamin-1	Dnm1	P39053	0	0	27(36%)
Thrombospondin 1	Thbs1	P35441	9(10%)	0	4(3%)
Peptidyl-prolyl cis-trans isomerase FKBP4	Fkbp4	P30416	0	5	6(14%)
Neuropilin-1	Nrp1	P97333	0	0	12(18%)
Treacle Protein	Tcof	O08784	5(5%)	0	0
Y-box-binding protein 2	Ybox2	Q9Z2C8	5(12%)	0	0
Multidrug resistance protein 1	Mdr1	P06795	0	0	6(8%)
Fibulin 2	Fbln2	P37889	3(4%)	3(4%)	0
Matrix metalloproteinase-14	Mmp14	P53690	0	0	4(6%)

Proteins identified by LC MS/MS analysis of the nuclear, cytoplasmic and membrane subfractions of CD105+ cells were compared to those identified from CD105− cells in each of 3 replicate experiments. Unique proteins were sorted according to their Scaffold rank scores. The 25 highest ranking proteins are listed along with the number of unique peptides identified and percentage of protein coverage from a representative experiment (n = 3). Also listed are the subcellular fractions from which each protein was consistently isolated in each of the three experiments.

**Figure 3:**
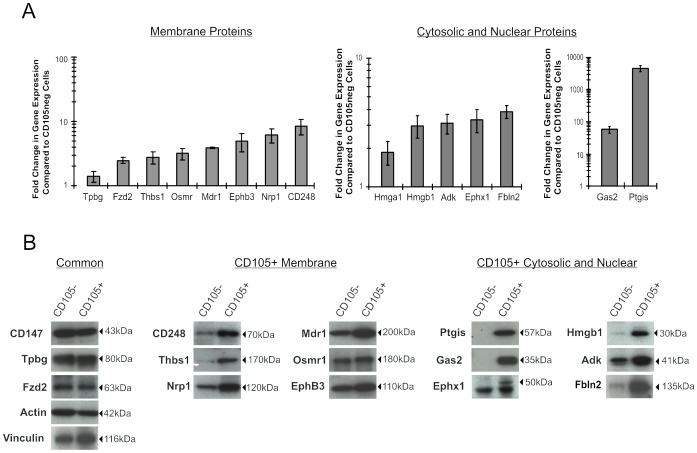
Real-time PCR and Western Blot analysis validate potential markers of MSC that are associated with distinct functional networks. The expression levels of 16 proteins that were identified as either common to all stroma, or unique to CD105+ cells, were chosen for verification based on the availability of reliable antibodies. (A) Comparison of gene expression levels in CD105+ and CD105− cells. An aliquot of the CD105+ and CD105− cells used for LC MS/MS was utilized for mRNA isolation and converted to cDNA for real-time RT PCR comparison of gene expression (n = 3). Results were normalized to GAPDH and fold change in gene expression was determined in comparison to levels detected in CD105− cells. (B) Western Blot analysis of protein expression levels in CD105+ and CD105− cells. In three separate experiments, whole proteins extracts were obtained from CD105+ and CD105− cells grown to the identical passage as used for LC-MS/MS experiments. Equivalent amounts of each protein extract were probed by Western Blot for the presence of common or unique proteins. Analysis of either actin or vinculin expression was completed on each blot to act as a loading control.

To compare the gene expression levels of these proteins, mRNA was extracted from a portion of the same CD105+ and CD105− cells used for LC MS/MS analysis. Gene expression levels for candidate MSC markers were determined by real-time RT PCR and normalized with actin expression. The gene expression levels for 3 proteins that were identified as common between CD105+ and CD105− cells (Trophoblast glycoprotein (Tpbg), Frizzled-2 (Fzd2) and High mobility group AT-hook 1 (Hmga1)) were utilized as controls for relative quantification. As expected, less than 2-fold differences in mRNA transcript were detected for Tpbg and Hmga1 in CD105+ and CD105− cell types; however, Fzd2 transcript was upregulated 2.5±0.3 fold in CD105+ cells (Figure 3A). Each of the 12 candidate MSC markers tested were detected in CD105+ cells at levels greater than 2 fold compared to CD105− cells. Of particular interest were the Ptgis mRNA which was exclusively identified in CD105+ cells, as well as Gas2 and CD248 that were expressed at 58±13 and 8.5±2.3 fold greater levels compared to CD105− cells.

Previous work from our group has demonstrated that mRNA expression levels in stromal cells, as detected by real-time RT PCR, do not always correlate with the amount of protein present in the cell [23]. To this end, the 12 candidate markers identified by LC MS/MS were further validated by Western Blotting. Whole cell protein extracts isolated from CD105+ and CD105− cells were confirmed to contain equivalent amounts of CD147, Tpbg and Actin (Figure 3B). As well, Western Blot analysis of Fzd2 expression demonstrated that while mRNA transcripts were upregulated in CD105+ cells, the amount of protein detected in both cell types was the same. A clear discrepancy between gene expression profiles and Western Blot analysis was also determined for Osmr1 and EphB3 which showed little to no quantitative differences at the protein level despite a 3.2±0.6 and 4.9±1.5 fold higher level of mRNA expression. These results re-iterate the importance of utilizing both protein and gene expression methods of quantification when investigating biological systems.

Of the membrane proteins detected only in CD105+ cell extracts by LC MS/MS, CD248 and Thbs1 were confirmed as highly upregulated by Western Blotting while Nrp1 and Mdr1 showed a more modest level of enrichment. Western Blot analysis of nuclear and cytosolic proteins that were characteristic of CD105+ cells showed clear differences compared to CD105− stroma for each of the 6 candidate markers. The largest differences in expression, were found in Ptgis, Gas2, Ephx1 proteins where distinct bands corresponding to these proteins were detected in CD105+ cell extracts, but notably absent in extracts derived from CD105− cells. These results were confirmed over three different protein concentrations (data not shown). Hmgb1, Fbln2 and Adk proteins were also enriched in CD105+ cells, although to a lower extent compared to the other candidate MSC markers. Overall, real-time RT-PCR and Western Blot analysis have confirmed 10 candidate markers for the discrimination of MSC and non-multipotent stroma *in vitro*. These candidate markers include both membrane (CD248, Thbs1, Nrp1 and Mdr1) and nuclear or cytosolic (Ptgis, Gas2, Ephx1, Hmgb1, Adk and Fbln2) associated proteins.

### Candidate markers of murine MSC are detectable in human bone marrow stromal cultures containing MSC

**Figure 4:**
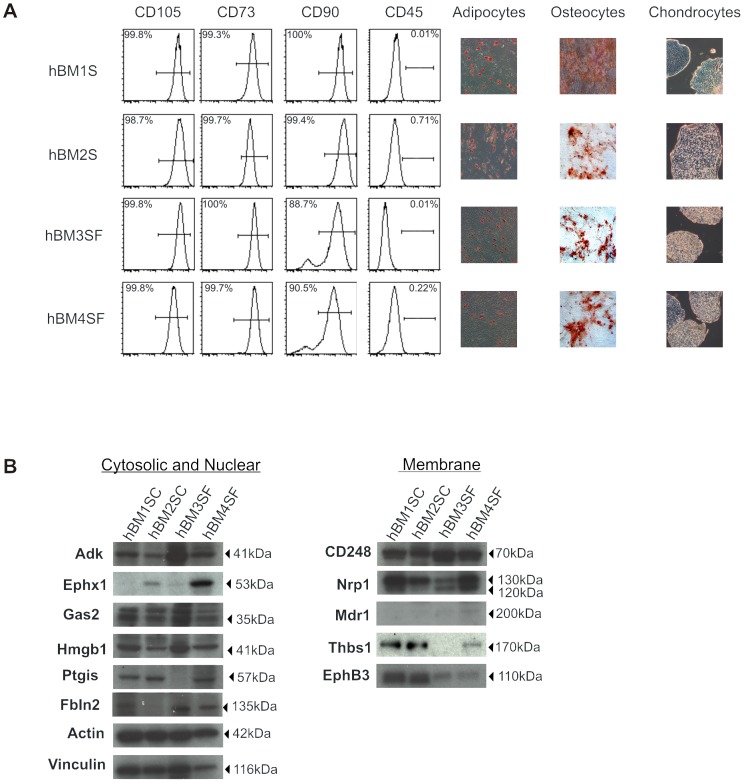
Standard and serum free human stromal cultures containing MSC, harbour cells that express candidate markers of murine MSC at varying quantities. Human bone marrow derived stromal cultures were initiated from 4 different donors in either serum containing (SC) or serum free (SF) conditions. (A) Human stromal cultures containing MSC can be derived in the either SC or SF media. Cultures were analyzed after first passage by both flow cytometry and in multipotent differentiation assays according to ISCT established criteria for MSC. Gates established based on unstained controls were used to compare the frequency of CD105, CD73, CD90 and CD45 expressing cells. Evidence of adipocyte, osteocyte and chondrocyte differentiation from each culture was verified by Oil Red O, Alizarin Red and Alcian Blue staining respectively (n = 4). (B) Extracts from human MSC containing stromal cultures express murine MSC specific proteins at frequencies that vary according to both donor and culture condition. An equal quantity of whole cell protein extracts was analyzed by Western Blotting for the presence of murine MSC associated proteins. The molecular weight of each protein is shown at the right of each blot. Nrp1 antibody recognized both modified (130 kDa) and unmodified (120 kDa) forms. Individual blots were analyzed for either actin or vinculin as a control for protein loading.

To determine the potential utility of our identified markers of murine MSC towards the identification of human MSC, we generated human bone marrow stromal cultures from four separate donors to use for marker validation. To determine the effect of growth conditions on protein expression, cultures were generated using both serum containing (SC) and serum free (SF) media. Early passage adherent cells were analyzed for stromal cell purity by flow cytometry according to criteria described by the International Society of Cell Therapy [28]. As demonstrated in Figure 4A, all cell lines lacked cells expressing CD45, demonstrating that they were free of hematopoietic cell contamination. Cultures were also found to be free of cells expressing CD34 (data not shown). Stromal cell purity was further demonstrated in both SC and SF cultures by the detection of CD105, CD73 and CD90 on the surface of greater than 90% of all cells.

To determine the presence of MSC, an aliquot of cells from each culture was plated in media that promotes the differentiation of adipocyte, osteocyte or chondrocyte precursors (Figure 4A). Regardless of the presence of serum, cultures derived from each of the 4 donors tested were found to contain MSC as demonstrated by the presence of cells staining positive for Oil Red O (adipocytes), Alizarin Red (osteocytes) and Alcian Blue (chondrocytes). Together, flow cytometric and multipotent differentiation assays demonstrate the successful generation of human bone marrow stromal cultures containing MSC using both SC and SF media.

To analyze the relative expression levels of candidate MSC markers in human bone marrow derived stromal cultures, we utilized Western Blot. Whole cell proteins were extracted from early passage cultures shown above to have multipotent function. Antibodies specific to the human proteins corresponding to those identified in murine stroma, were used to probe equal amounts of whole protein lysate derived from each of the four human stromal cultures (Figure 4B). To control for errors in loading, individual blots were stripped and reprobed using antibodies specific to either actin or vinculin. Of the candidate marker proteins analyzed, Ptgis, Ephx1, Fbln2, Mdr1 and Thbs1 were not detected in all human stromal cultures containing MSC; suggesting that they may not be markers of human MSC. Interestingly, the detection of Ephx1 (hBM1SC, hBM4SF) and Ptgis (hBM1SC, hBM2SC, hBM4SF) seemed to vary by donor. In contrast, the expression of Thbs1 and EphB3 proteins varied by condition, being detected at much higher levels in cultures propagated with serum. The Mdr1 protein, which was highly enriched in murine CD105+ cells, was not detectable in human stromal cultures containing MSC. A lack of Mdr-1 expression on human stroma was confirmed by flow cytometry (data not shown).

Western blot analysis of human stromal cell lysates did identify five proteins; Adk; Gas2; Hmgb1; CD248; and Nrp1 in all MSC containing stromal cultures regardless of donor origin or culture conditions. CD248 was the only protein that was detected at similar quantities in all cultures tested. The pattern of expression for intracellular proteins, Adk, Gas2 and Hmgb1, was similar and seemed to be dependent upon donor, with higher levels identified in hBM1SC and hBM3SF. By contrast, the membrane associated protein Nrp1 was detected at similar amounts in hBM1SC and hBM4SF but showed reduced expression in hBM2SC and hBM3SF. Interestingly, both modified (130 kDa) and unmodified (120 kDa) forms of Nrp1 were detected in each of the cell culture derived lysates. The ratio of modified to unmodified protein also varied by donor and did not correlate directly with the overall amount of Nrp1 protein detected in each lysate. Stromal cells derived from hBM2SC did not express unmodified Nrp1 at levels detectable by Western Blot. These data suggest that variable amounts of activated and inactivated Nrp1 are present in stromal cultures derived from different donors. Overall, our work demonstrates that Adk, Gas2, Hmgb1, Nrp1 and CD248 protein expression may represent a novel signature of human bone marrow derived MSC *in vitro*.

## Discussion

With the vast number of recent studies providing pre-clinical evidence for the potential therapeutic utility of stromal cell cultures, it has become increasingly important to address their heterogeneity as a measure of both safety and effectiveness in a clinical setting. This fact is underscored by the variable results that have been collected from clinical studies on graft versus host disease by different groups [14], [17]. The identification of prospective markers for stromal cells based on their therapeutic function could have immense benefit in both clinical and non-clinical settings. To further this goal, a non-quantitative LC MS/MS approach was utilized in an attempt to identify proteins expressed in MSC, that were not detectable in non-multipotent murine stroma. Several proteins were differentially detected in multipotent or non-multipotent murine stromal cells that could be further tested as candidate markers of human MSC. One measure of the accuracy of our data was provided by the identification of proteins previously associated with both human and murine stromal cells, such as CD248 (Endosialin) and Nrp1. CD248 is a membrane bound glycoprotein that is a known *in vivo* marker of stromal cells that support lymphoid development and splenic repair [29], [30]. In addition, cultured human stromal cells have been shown to express CD248 using both flow cytometric and real-time RT PCR analysis [30]. Our identification of CD248 expression in MSC containing cultures not only provides an indication of the accuracy of our method for determining potential markers of MSC, but suggests a more specific role for this protein in regulating stromal cell multipotency.

Proteins detected exclusively in murine MSC and non-multipotent stroma by LC MS/MS were found to group into distinct canonical pathways by IPA analysis. Interestingly, three of the four MSC specific canonical pathways included proteins that have been previously implicated in the proliferation and differentiation of stem cells. These include cyclin dependent kinases (CDK2 and 4) and protein kinase family members (AKT1, PRKACB) of which reduced expression has been shown to promote differentiation in MSC and other somatic stem cells [31]–[33]. These data provide further support for the reliability of our approach. As many of the proteins identified in this study have yet to be associated with stem cells, it is interesting to speculate that the lists produced from this work may include novel regulators of MSC function.

The current manuscript describes twenty-five different proteins that were uniquely identified in murine MSC enriched cells by LC MS/MS, ten of which were verified using real-time RT PCR and Western Blot. Of these, only five were further shown to be expressed by human stromal cultures containing MSC. This finding may be indicative of the distinct differences that have been previously reported between human and murine derived MSC. Such differences include changes in surface phenotype, growth rate, and the frequency of transformation events [34], [35]. In particular, our finding that the Mdr1 protein, an ABC transporter efflux pump responsible for the side population (SP) phenotype of several different types of somatic stem cells [36]–[38], was expressed in murine but not human stroma correlates with previous *in vivo* studies. Specifically, MSC have been found to exist in murine bone marrow cells with an SP phenotype but not in human bone marrow SP cells [35]. Nonetheless, it is important to note that despite some inherent differences, murine stromal cells may still provide an appropriate model for studying MSC as differences in the differentiation potential, immune suppressive capacity and tissue restorative function of murine and human MSC have yet to be discovered. In all, the markers identified here could provide a useful means for further purification of murine or human MSC which may allow more accurate study of the therapeutic utility of stromal cell cultures.

An additional protein reported in our study to be exclusively detected in murine CD105+ is Ephx1. Ephx1 has been previously associated with adipogenesis in MSC through the activation of cryoprotective lipid mediators [39]. Thus, the varied expression of Ephx1 in human stromal cultures identified here may indicate that this protein is an early marker of adipocyte committed progenitors within cultured stroma. In support of this, our previous work demonstrated that clones of adipocyte restricted progenitors exist within the CD105+ fraction of murine stromal cultures, while such clones could not be detected in CD105− fractions [20]. Thus, we suggest that the identification of Ephx1 expressing cells in human stromal cultures, may be an indication of increased numbers of cells that have committed to the adipocyte lineage.

Nrp1 is a membrane associated glycoprotein that functions as a co-receptor for vascular endothelial growth factors and plexins [40] with a primary function in vascular and neural development. Ball and others demonstrated that human stromal cells express Nrp1 which co-localizes with platelet derived growth factor (PDGF) receptors, initiating cell migration and proliferation in response to PDGFs and VEGF-A [41]. This finding correlates with reports from several other groups that have demonstrated PDGF-BB to be an important factor in the maintenance and expansion of MSC in culture [42]–[44]. The association of Nrp1 expression with both human and murine MSC in our study supports this body of work. In addition, identification of greater quantities of Nrp1 in a murine stromal population enriched for multipotent function suggests that signalling through this receptor may be enhanced in MSC. Of particular interest in human stromal cells are the differences uncovered in both the overall quantity of Nrp1 and the relative amounts of modified and unmodified protein between bone marrow donors. The significance of this requires further investigation but adds to the current body of evidence demonstrating heterogeneity between stromal cultures derived from different donors. Overall, our data support previous work demonstrating an important role for Nrp1 and PDGF signalling in MSC and suggest that differences in Nrp1 expression levels may provide a suitable marker for discriminating MSC from non-multipotent stroma.

The work presented here represents the first association of elevated Hmgb1 expression levels in stromal cells with multipotent function. Hmgb1 was originally identified as a nuclear protein that regulates gene transcription through nucleosome binding and stabilisation [45]–[47]. Since that time it has been further demonstrated to function as a cytokine with the capacity to regulate inflammatory immune responses [48]–[50] and participate in cardiac repair [51], [52], functions that have also been ascribed to MSC. Thus, understanding the role of enhanced Hmgb1 expression in MSC may provide further insights into their clinical utility. Of further interest are the findings that stromal cells express the putative receptors of Hmgb1, Toll-like receptor-2 and 4 proteins [53], [54], and are capable of migration and proliferation *in vitro* in response to Hmgb1 [55], [56]. These findings, together with our work, suggest the potential for autocrine signalling mechanisms in MSC through Hmgb1. Overall, our work suggests Hmgb1 as an important candidate as both a distinguishing marker and potential regulator of bone marrow derived MSC function.

Several different culture conditions have been reported to allow the maintenance and expansion of human stromal cells [44], [57]–[60]. While standard conditions contain 10 to 15 percent FBS, the potential clinical utility of stromal cells has pushed the development of serum and animal component-free culture media. Compared to standard media conditions, human stromal cells grown in serum free media have been shown to have comparable multipotent capacity but expand more rapidly and have a slightly increased capacity to suppress T-cell activation [58]. Our study provides evidence that the removal of serum has minimal effects on the protein expression levels, at least for the ten potential MSC markers evaluated. Of these, only Thbs1 was found to be differentially expressed in serum free cultures compared to cells grown in standard conditions. In fact, the majority of differences identified between cell lines were identified between donors. While the number of samples tested in our study is not sufficient to make definitive conclusions regarding donor variation, the differences found in protein expression levels between samples emphasize the degree of heterogeneity present in human bone marrow derived stromal cell cultures.

Overall, we have identified a list of 25 proteins that provide a candidate signature for MSC within stromal cell cultures. Five proteins specifically (Adk, Gas2, Hmgb1, Nrp1 and CD248) were verified to be expressed at significantly higher levels in murine MSC and detected in both serum free and serum supplemented human stromal cultures containing MSC. With further validation, these proteins may provide a useful tool for addressing stromal culture heterogeneity and further characterizing the therapeutic potential of MSC.

## Supporting Information

Table S1 **Sequences for primers used in real time RT-PCR verification of candidate MSC markers in CD105+ and CD105− stroma.**
(DOC)

Table S2 **List of antibodies used for Western Blot verification of candidate murine and human MSC markers.**
(DOC)

Table S3 **Protein identification by UPLC LTQ-FT MS/MS analyses and Mascot database search.**
(DOC)
