# Supplementary material for: Identification of a Candidate Proteomic Signature to Discriminate Multipotent and Non-Multipotent Stromal Cells
Source: PLoS One. 2012 Jun 13;7(6):e38954. doi: 10.1371/journal.pone.0038954 (PMC3374805; doi:10.1371/journal.pone.0038954)
Supplement: Table S1 — Sequences for primers used in real time RT-PCR verification of candidate MSC markers in CD105+ and CD105− stroma. (DOC) [file pone.0038954.s001.doc]

|  | Forward | Reverse |
| --- | --- | --- |
| TPBG | CCAAACCGAGCCACGAGAGAGGA | GGAGCTGCACAGGTTACATCCGC |
| FZD2 | GCTGCGAGCATTTCCCGCGT | TGAGTAGCGCAGGAGCTCCGT |
| THBS1 | GGACTTCTGCAGGCAATCGCGA | CGCTGAGTCCGGTGAAGACGC |
| OSMR | GCCCCCACCCCAAGCTGTATCTA | ACCAGACAACCAAGCGTGCAAAAAG |
| MDR1 | GCAGTGGCTCTTGAAGCCGTAAGA | TCAAACTCCATCACCACCTCACGTG |
| EPHB3 | GGTCCGTCCAGATTCCCGCG | AGGCTCGCGCACAGTCCAAG |
| NRP1 | GGGGCAGGGTTTTCCATCCGC | GGTGCACTCCAAGCAGTTGGGG |
| CD248 | GGCCCACTAACCAGACCTCATCTATCA | AGGGCAGCTGTGGCACTGATTT |
| HMGA1 | CCGGGGCTGCGCTCCTCTAA | GCGGCGAACTCACGCCTTCT |
| HMGB1 | CGTCTGGCTCCCGCTCTCAC | GGCACAGAGTCGCCCAGTGC |
| ADK | AGCACCTTGACCTGGAGCGGA | TCGGCAGCATAGCGAGCCAC |
| EPHX1 | CGGCGACTGGGGGTCTCTCA | GGCCCAGGAGAGGGGTCAGG |
| FBLN2 | CAGTGCTGTGTGCAGAGGGCTA | GGGTGCACGTGTGCAGGTCT |
| GAS2 | CGCATGCTGCAGATCTCCCGT | CCTTGTAGGTGGCAGAGACCACCA |
| PTGIS | GCACGGTCCTGCTGGGTGAC | GCGCTGAGCAGGGCGTTGTA |
| ACTIN | GCCTCACTGTCCACCTTCCA | GGGCCGGACTCATCGTACT |
|  |  |  |
