# Supplementary material for: Identification of a Candidate Proteomic Signature to Discriminate Multipotent and Non-Multipotent Stromal Cells
Source: PLoS One. 2012 Jun 13;7(6):e38954. doi: 10.1371/journal.pone.0038954 (PMC3374805; doi:10.1371/journal.pone.0038954)
Supplement: Table S2 — List of antibodies used for Western Blot verification of candidate murine and human MSC markers. (DOC) [file pone.0038954.s002.doc]

Supplementary Table 2

| Antibody | Company - Cat # | Species Reactivity | Dilution |
| --- | --- | --- | --- |
|  |  |  |  |
| ADK | Abcam - ab38010 | Human, mouse | 1:1,000 |
| CD147 | Novus Biologicals - NBP1-19677 | Mouse | 1:1,000 |
| CD248 | Protein Tech Group - 18160-1-AP | Human, mouse | 1:600 |
| EphB3 | Abcam - ab76885 | Human, mouse | 1:10,000 |
| Ephx1 | Abcam - ab76226 | Human | 1:500 |
| Ephx1  Clone#AT2E5 | Novus Biologicals - NBP1-30163 | Mouse | 1:1,000 |
| Fibulin 2 | Novus Biologicals – NBP1-74628 | Human, mouse | 1:500, 1:2000 |
| Frizzled 2 | Abcam - ab75084 | Mouse | 1:1,000 |
| Gas 2  Clone #4E11 | Abnova - H00002620-M01 | Human, mouse | 1:5,000,1:10,000 |
| Hmgb1 | Protein Tech Group - 10829-1-AP | Human, mouse | 1:500, 1,1000 |
| Mdr-1  (P Glycoprotein)  Clone #C219 | Abcam - ab3364 | Human, mouse | 1:200, 1:100 |
| NRP1  Clone #EPR3113 | Abcam - ab81321 | Human, mouse | 1:1,000 |
| OSMR β | Santa Cruz - sc-30010 | Human | 1:1,000 |
| OSMR β | Santa Cruz - sc-30011 | Mouse | 1:500 |
| PTGIS | Abcam - ab23668 | Human, mouse | 1:2,000, 1:4,000 |
| Thbs1 | Lifespan Biosciences - LS-B2570 | Human, mouse | 1:500 |
| Tpbg | R&D Systems – AF5049 | Mouse | 1:200 |
