# Supplementary material for: Identification of a Candidate Proteomic Signature to Discriminate Multipotent and Non-Multipotent Stromal Cells
Source: PLoS One. 2012 Jun 13;7(6):e38954. doi: 10.1371/journal.pone.0038954 (PMC3374805; doi:10.1371/journal.pone.0038954)
Supplement: Table S3 — Protein identification by UPLC LTQ-FT MS/MS analyses and Mascot database search. (DOC) [file pone.0038954.s003.doc]

**Table S3. Protein identification by UPLC LTQ-FT MS/MS analyses and Mascot database search**

| 1. [HMGB1_MOUSE](http://10.139.25.109/mascot/cgi/protein_view.pl?file=../data/20120413/F007791.dat&hit=HMGB1_MOUSE&db_idx=1&px=1&ave_thresh=1&_ignoreionsscorebelow=20&report=0&_sigthreshold=0.05&_msresflags=1089&_msresflags2=2&percolate=-1&percolate_rt=0)  **Mass:** 25049 **Score:** 1592 **Matches:** 34(34) **Sequences:** 19(19)  High mobility group protein B1  Sequence Coverage: **54%**, Matched peptides shown in **Bold Red**  **1** MGKGDPKKPR GK**MSSYAFFV QTCREEHKKK HPDASVNFSE FSKK**CSERWK TMSAKEK**GKF EDMAK**ADKAR YEREMK**TYIP PKGETK**KK**FK DPNAPKRPPS**  **101 AFFLFCSEYR PKIKGEHPGL SIGDVAKKLG EMWNNTAADD KQPYEKK**AAK LKEK**YEKDIA AYR**AKGKPDA AKKGVVKAEK SKKKKEEEDD EEDEEDEEEE  **201** EEEEDEDEEE DDDDE  **Start - End Observed Mr(expt) Mr(calc) ppm Miss Sequence**  **13 - 24 748.8367 1495.6589 1495.6588 0 0 K.MSSYAFFVQTCR.E**  ([Ions score 93](http://10.139.25.109/mascot/cgi/peptide_view.pl?file=../data/20120413/F007791.dat&query=22402&hit=1&index=HMGB1_MOUSE&px=1&section=5&ave_thresh=1&_ignoreionsscorebelow=20&report=0&_sigthreshold=0.05&_msresflags=1089&_msresflags2=2&percolate=-1&percolate_rt=0))  **13 - 28 505.7314 2018.8963 2018.8979 -1 1 K.MSSYAFFVQTCREEHK.K**  ([Ions score 25](http://10.139.25.109/mascot/cgi/peptide_view.pl?file=../data/20120413/F007791.dat&query=40465&hit=1&index=HMGB1_MOUSE&px=1&section=5&ave_thresh=1&_ignoreionsscorebelow=20&report=0&_sigthreshold=0.05&_msresflags=1089&_msresflags2=2&percolate=-1&percolate_rt=0))  **13 - 29 716.6724 2146.9938 2146.9928 0 2 K.MSSYAFFVQTCREEHKK.K**  ([Ions score 39](http://10.139.25.109/mascot/cgi/peptide_view.pl?file=../data/20120413/F007791.dat&query=43456&hit=1&index=HMGB1_MOUSE&px=1&section=5&ave_thresh=1&_ignoreionsscorebelow=20&report=0&_sigthreshold=0.05&_msresflags=1089&_msresflags2=2&percolate=-1&percolate_rt=0))  **29 - 43 574.2931 1719.8576 1719.8580 0 2 K.KKHPDASVNFSEFSK.K**  ([Ions score 30](http://10.139.25.109/mascot/cgi/peptide_view.pl?file=../data/20120413/F007791.dat&query=30399&hit=1&index=HMGB1_MOUSE&px=1&section=5&ave_thresh=1&_ignoreionsscorebelow=20&report=0&_sigthreshold=0.05&_msresflags=1089&_msresflags2=2&percolate=-1&percolate_rt=0))  **29 - 43 860.9368 1719.8591 1719.8580 1 2 K.KKHPDASVNFSEFSK.K**  ([Ions score 59](http://10.139.25.109/mascot/cgi/peptide_view.pl?file=../data/20120413/F007791.dat&query=30402&hit=1&index=HMGB1_MOUSE&px=1&section=5&ave_thresh=1&_ignoreionsscorebelow=20&report=0&_sigthreshold=0.05&_msresflags=1089&_msresflags2=2&percolate=-1&percolate_rt=0))  **30 - 43 796.8884 1591.7623 1591.7631 0 1 K.KHPDASVNFSEFSK.K**  ([Ions score 100](http://10.139.25.109/mascot/cgi/peptide_view.pl?file=../data/20120413/F007791.dat&query=25849&hit=1&index=HMGB1_MOUSE&px=1&section=5&ave_thresh=1&_ignoreionsscorebelow=20&report=0&_sigthreshold=0.05&_msresflags=1089&_msresflags2=2&percolate=-1&percolate_rt=0))  **30 - 43 398.9477 1591.7615 1591.7631 -1 1 K.KHPDASVNFSEFSK.K**  ([Ions score 21](http://10.139.25.109/mascot/cgi/peptide_view.pl?file=../data/20120413/F007791.dat&query=25847&hit=1&index=HMGB1_MOUSE&px=1&section=5&ave_thresh=1&_ignoreionsscorebelow=20&report=0&_sigthreshold=0.05&_msresflags=1089&_msresflags2=2&percolate=-1&percolate_rt=0))  **30 - 43 531.5946 1591.7620 1591.7631 -1 1 K.KHPDASVNFSEFSK.K**  ([Ions score 39](http://10.139.25.109/mascot/cgi/peptide_view.pl?file=../data/20120413/F007791.dat&query=25848&hit=1&index=HMGB1_MOUSE&px=1&section=5&ave_thresh=1&_ignoreionsscorebelow=20&report=0&_sigthreshold=0.05&_msresflags=1089&_msresflags2=2&percolate=-1&percolate_rt=0))  **30 - 44 574.2933 1719.8582 1719.8580 0 2 K.KHPDASVNFSEFSKK.C**  ([Ions score 33](http://10.139.25.109/mascot/cgi/peptide_view.pl?file=../data/20120413/F007791.dat&query=30400&hit=1&index=HMGB1_MOUSE&px=1&section=5&ave_thresh=1&_ignoreionsscorebelow=20&report=0&_sigthreshold=0.05&_msresflags=1089&_msresflags2=2&percolate=-1&percolate_rt=0))  **30 - 44 430.9718 1719.8582 1719.8580 0 2 K.KHPDASVNFSEFSKK.C**  ([Ions score 33](http://10.139.25.109/mascot/cgi/peptide_view.pl?file=../data/20120413/F007791.dat&query=30401&hit=1&index=HMGB1_MOUSE&px=1&section=5&ave_thresh=1&_ignoreionsscorebelow=20&report=0&_sigthreshold=0.05&_msresflags=1089&_msresflags2=2&percolate=-1&percolate_rt=0))  **31 - 43 732.8387 1463.6628 1463.6681 -4 0 K.HPDASVNFSEFSK.K**  ([Ions score 88](http://10.139.25.109/mascot/cgi/peptide_view.pl?file=../data/20120413/F007791.dat&query=21476&hit=1&index=HMGB1_MOUSE&px=1&section=5&ave_thresh=1&_ignoreionsscorebelow=20&report=0&_sigthreshold=0.05&_msresflags=1089&_msresflags2=2&percolate=-1&percolate_rt=0))  **31 - 44 531.5953 1591.7640 1591.7631 1 1 K.HPDASVNFSEFSKK.C**  ([Ions score 34](http://10.139.25.109/mascot/cgi/peptide_view.pl?file=../data/20120413/F007791.dat&query=25851&hit=1&index=HMGB1_MOUSE&px=1&section=5&ave_thresh=1&_ignoreionsscorebelow=20&report=0&_sigthreshold=0.05&_msresflags=1089&_msresflags2=2&percolate=-1&percolate_rt=0))  **58 - 65 463.2259 924.4373 924.4375 0 1 K.GKFEDMAK.A**  ([Ions score 39](http://10.139.25.109/mascot/cgi/peptide_view.pl?file=../data/20120413/F007791.dat&query=2903&hit=1&index=HMGB1_MOUSE&px=1&section=5&ave_thresh=1&_ignoreionsscorebelow=20&report=0&_sigthreshold=0.05&_msresflags=1089&_msresflags2=2&percolate=-1&percolate_rt=0))  **58 - 65 309.1529 924.4368 924.4375 -1 1 K.GKFEDMAK.A**  ([Ions score 25](http://10.139.25.109/mascot/cgi/peptide_view.pl?file=../data/20120413/F007791.dat&query=2901&hit=1&index=HMGB1_MOUSE&px=1&section=5&ave_thresh=1&_ignoreionsscorebelow=20&report=0&_sigthreshold=0.05&_msresflags=1089&_msresflags2=2&percolate=-1&percolate_rt=0))  **77 - 86 567.3147 1132.6148 1132.6128 2 1 K.TYIPPKGETK.K**  ([Ions score 35](http://10.139.25.109/mascot/cgi/peptide_view.pl?file=../data/20120413/F007791.dat&query=9408&hit=1&index=HMGB1_MOUSE&px=1&section=5&ave_thresh=1&_ignoreionsscorebelow=20&report=0&_sigthreshold=0.05&_msresflags=1089&_msresflags2=2&percolate=-1&percolate_rt=0))  **89 - 96 458.7477 915.4809 915.4814 -1 1 K.FKDPNAPK.R**  ([Ions score 32](http://10.139.25.109/mascot/cgi/peptide_view.pl?file=../data/20120413/F007791.dat&query=2673&hit=1&index=HMGB1_MOUSE&px=1&section=5&ave_thresh=1&_ignoreionsscorebelow=20&report=0&_sigthreshold=0.05&_msresflags=1089&_msresflags2=2&percolate=-1&percolate_rt=0))  **89 - 96 306.1674 915.4804 915.4814 -1 1 K.FKDPNAPK.R**  ([Ions score 46](http://10.139.25.109/mascot/cgi/peptide_view.pl?file=../data/20120413/F007791.dat&query=2668&hit=1&index=HMGB1_MOUSE&px=1&section=5&ave_thresh=1&_ignoreionsscorebelow=20&report=0&_sigthreshold=0.05&_msresflags=1089&_msresflags2=2&percolate=-1&percolate_rt=0))  **97 - 112 668.0047 2000.9923 2000.9931 0 0 K.RPPSAFFLFCSEYRPK.I**  ([Ions score 27](http://10.139.25.109/mascot/cgi/peptide_view.pl?file=../data/20120413/F007791.dat&query=39994&hit=1&index=HMGB1_MOUSE&px=1&section=5&ave_thresh=1&_ignoreionsscorebelow=20&report=0&_sigthreshold=0.05&_msresflags=1089&_msresflags2=2&percolate=-1&percolate_rt=0))  **113 - 127 380.9660 1519.8350 1519.8358 -1 1 K.IKGEHPGLSIGDVAK.K**  ([Ions score 22](http://10.139.25.109/mascot/cgi/peptide_view.pl?file=../data/20120413/F007791.dat&query=23189&hit=1&index=HMGB1_MOUSE&px=1&section=5&ave_thresh=1&_ignoreionsscorebelow=20&report=0&_sigthreshold=0.05&_msresflags=1089&_msresflags2=2&percolate=-1&percolate_rt=0))  **113 - 127 760.9248 1519.8350 1519.8358 -1 1 K.IKGEHPGLSIGDVAK.K**  ([Ions score 71](http://10.139.25.109/mascot/cgi/peptide_view.pl?file=../data/20120413/F007791.dat&query=23191&hit=1&index=HMGB1_MOUSE&px=1&section=5&ave_thresh=1&_ignoreionsscorebelow=20&report=0&_sigthreshold=0.05&_msresflags=1089&_msresflags2=2&percolate=-1&percolate_rt=0))  **113 - 127 507.6198 1519.8377 1519.8358 1 1 K.IKGEHPGLSIGDVAK.K**  ([Ions score 60](http://10.139.25.109/mascot/cgi/peptide_view.pl?file=../data/20120413/F007791.dat&query=23202&hit=1&index=HMGB1_MOUSE&px=1&section=5&ave_thresh=1&_ignoreionsscorebelow=20&report=0&_sigthreshold=0.05&_msresflags=1089&_msresflags2=2&percolate=-1&percolate_rt=0))  **113 - 127 507.6204 1519.8393 1519.8358 2 1 K.IKGEHPGLSIGDVAK.K**  ([Ions score 48](http://10.139.25.109/mascot/cgi/peptide_view.pl?file=../data/20120413/F007791.dat&query=23203&hit=1&index=HMGB1_MOUSE&px=1&section=5&ave_thresh=1&_ignoreionsscorebelow=20&report=0&_sigthreshold=0.05&_msresflags=1089&_msresflags2=2&percolate=-1&percolate_rt=0))  **113 - 128 550.3175 1647.9307 1647.9308 0 2 K.IKGEHPGLSIGDVAKK.L**  ([Ions score 46](http://10.139.25.109/mascot/cgi/peptide_view.pl?file=../data/20120413/F007791.dat&query=27936&hit=1&index=HMGB1_MOUSE&px=1&section=5&ave_thresh=1&_ignoreionsscorebelow=20&report=0&_sigthreshold=0.05&_msresflags=1089&_msresflags2=2&percolate=-1&percolate_rt=0))  **113 - 128 412.9901 1647.9312 1647.9308 0 2 K.IKGEHPGLSIGDVAKK.L**  ([Ions score 43](http://10.139.25.109/mascot/cgi/peptide_view.pl?file=../data/20120413/F007791.dat&query=27939&hit=1&index=HMGB1_MOUSE&px=1&section=5&ave_thresh=1&_ignoreionsscorebelow=20&report=0&_sigthreshold=0.05&_msresflags=1089&_msresflags2=2&percolate=-1&percolate_rt=0))  **115 - 127 640.3366 1278.6587 1278.6568 1 0 K.GEHPGLSIGDVAK.K**  ([Ions score 55](http://10.139.25.109/mascot/cgi/peptide_view.pl?file=../data/20120413/F007791.dat&query=15790&hit=1&index=HMGB1_MOUSE&px=1&section=5&ave_thresh=1&_ignoreionsscorebelow=20&report=0&_sigthreshold=0.05&_msresflags=1089&_msresflags2=2&percolate=-1&percolate_rt=0))  **128 - 146 746.6883 2237.0415 2237.0423 0 2 K.KLGEMWNNTAADDKQPYEK.K** ([Ions score 77](http://10.139.25.109/mascot/cgi/peptide_view.pl?file=../data/20120413/F007791.dat&query=44770&hit=1&index=HMGB1_MOUSE&px=1&section=5&ave_thresh=1&_ignoreionsscorebelow=20&report=0&_sigthreshold=0.05&_msresflags=1089&_msresflags2=2&percolate=-1&percolate_rt=0))  **129 - 146 703.9901 2108.9469 2108.9473 0 1 K.LGEMWNNTAADDKQPYEK.K** ([Ions score 85](http://10.139.25.109/mascot/cgi/peptide_view.pl?file=../data/20120413/F007791.dat&query=42758&hit=1&index=HMGB1_MOUSE&px=1&section=5&ave_thresh=1&_ignoreionsscorebelow=20&report=0&_sigthreshold=0.05&_msresflags=1089&_msresflags2=2&percolate=-1&percolate_rt=0))  **129 - 146 1055.4805 2108.9454 2108.9473 1 1 K.LGEMWNNTAADDKQPYEK.K** ([Ions score 62](http://10.139.25.109/mascot/cgi/peptide_view.pl?file=../data/20120413/F007791.dat&query=42764&hit=1&index=HMGB1_MOUSE&px=1&section=5&ave_thresh=1&_ignoreionsscorebelow=20&report=0&_sigthreshold=0.05&_msresflags=1089&_msresflags2=2&percolate=-1&percolate_rt=0))  **129 - 147 560.2681 2237.0411 2237.0423 1 2 K.LGEMWNNTAADDKQPYEKK.A** ([Ions score 46](http://10.139.25.109/mascot/cgi/peptide_view.pl?file=../data/20120413/F007791.dat&query=44771&hit=1&index=HMGB1_MOUSE&px=1&section=5&ave_thresh=1&_ignoreionsscorebelow=20&report=0&_sigthreshold=0.05&_msresflags=1089&_msresflags2=2&percolate=-1&percolate_rt=0))  **129 - 147 746.6885 2237.0421 2237.0423 0 2 K.LGEMWNNTAADDKQPYEKK.A** ([Ions score 58](http://10.139.25.109/mascot/cgi/peptide_view.pl?file=../data/20120413/F007791.dat&query=44779&hit=1&index=HMGB1_MOUSE&px=1&section=5&ave_thresh=1&_ignoreionsscorebelow=20&report=0&_sigthreshold=0.05&_msresflags=1089&_msresflags2=2&percolate=-1&percolate_rt=0))  **155 - 163 376.8601 1127.5583 1127.5611 -2 1 K.YEKDIAAYR.A**  ([Ions score 24](http://10.139.25.109/mascot/cgi/peptide_view.pl?file=../data/20120413/F007791.dat&query=9148&hit=1&index=HMGB1_MOUSE&px=1&section=5&ave_thresh=1&_ignoreionsscorebelow=20&report=0&_sigthreshold=0.05&_msresflags=1089&_msresflags2=2&percolate=-1&percolate_rt=0))  **155 - 163 564.7871 1127.5597 1127.5611 -1 1 K.YEKDIAAYR.A**  ([Ions score 50](http://10.139.25.109/mascot/cgi/peptide_view.pl?file=../data/20120413/F007791.dat&query=9150&hit=1&index=HMGB1_MOUSE&px=1&section=5&ave_thresh=1&_ignoreionsscorebelow=20&report=0&_sigthreshold=0.05&_msresflags=1089&_msresflags2=2&percolate=-1&percolate_rt=0))  **155 - 163 376.8612 1127.5618 1127.5611 1 1 K.YEKDIAAYR.A**  ([Ions score 24](http://10.139.25.109/mascot/cgi/peptide_view.pl?file=../data/20120413/F007791.dat&query=9158&hit=1&index=HMGB1_MOUSE&px=1&section=5&ave_thresh=1&_ignoreionsscorebelow=20&report=0&_sigthreshold=0.05&_msresflags=1089&_msresflags2=2&percolate=-1&percolate_rt=0))  **158 - 163 354.6872 707.3599 707.3602 0 0 K.DIAAYR.A**  ([Ions score 23](http://10.139.25.109/mascot/cgi/peptide_view.pl?file=../data/20120413/F007791.dat&query=62&hit=1&index=HMGB1_MOUSE&px=1&section=5&ave_thresh=1&_ignoreionsscorebelow=20&report=0&_sigthreshold=0.05&_msresflags=1089&_msresflags2=2&percolate=-1&percolate_rt=0))  2. [CSPG4_MOUSE](http://10.139.25.109/mascot/cgi/protein_view.pl?file=../data/20120413/F007793.dat&hit=CSPG4_MOUSE&db_idx=1&px=1&ave_thresh=1&_ignoreionsscorebelow=20&report=0&_sigthreshold=0.05&_msresflags=1089&_msresflags2=2&percolate=-1&percolate_rt=0)    **Mass:** 252992   **Score:** 4229   **Matches:** 77(77)  **Sequences:** 63(63)  Chondroitin sulfate proteoglycan 4  Sequence Coverage: **34%**; Matched peptides shown in **Bold Red**  **1** MLLGPGHPLS APALALALTL ALLVRSTAPA SFFGENHLEV PVPSALTRVD LLLQFSTSQP EALLLLAAGQ DDHLLLQLHS GCLQVRLALG QKELKLQTPA  **101** DTVLSDSAPH TVVLTVSDSW AVLSVDGVLN TSAPIPRASH LKATYGLFVG SSGSLDLPYL KGISRPLR**GC LHSAILNGR**N LLRPLTSDVH EGCAEEFSAG  **201** DEVGLGFSGP HSLAAFPAWS TREEGTLEFT LTTR**SQQAPL AFQAGDKR**GN FIYVDIFEGH LRAVVEKGQG TMLLRNSVPV ADGQPHEVSV HIDVHR**LEIS**  **301 VDQYPTR**TFN R**GVLSYLEPR GSLLLGGLDT EASR**HLQEHR LGLAPGAANI SLVGCIEDFS VNGRRQGLR**D AWLTR**DMSAG CRPEEDEYEE EVYGPYETFS  **401** TLAPEAWPAM ELPEPCIPEP GLPAVFANFT QLLTISPLVV AEGGTAWLEW R**HVQPTLDLT EAELRKSQVL FSVSQSARHG DLELDILGAQ TRKMFTLLDV**  **501 VNRK**ARFVHD GSEDTSDQLM LEVSVTAR**AP VPSCLRRGQI YILPIQVNPV NDPPR**IIFPH GSLMVILEHT QKPLGPEIFQ AYDPDSACEG LTFQLLGVSS  **601** GVPVEHR**DQP GEPATEFSCR** ELEVGDIVYV HR**GGPAQDLT FRVSDGMQAS APATLK**VVAV RPAIQILHNT GLHLAQGSAA AILPANLSVE TNAVGQDVSV  **701** LFR**VTGTLQF GELQKQGAGG VEGTEWWDTL AFHQR**DVEQG RVRYLSTDPQ HHTQDTVEDL ILEVQVGQET LSNLSFPVTI QR**ATVWMLR**L EPLHTQNPHQ  **801** ETLTPAHLEA SLEEEEEEGS PQPHTFHYEL VQAPR**RGNLL LQGTRLSDGE SFSQSDLQAG R**VTYRATMR**T SEAADDSFR**F RVTSPPHFSP LYTFPIHIGG  **901** DPNAPVLTNV LLMVPEGGEG VLSADHLFVK **SLNSASYLYE VMEQPHHGK**L AWRDPKGK**ST PVTSFTNEDL LHGRLVYQHD DSETIEDDIP FVATR**QGEGS  **1001** GDMAWEEVRG VFR**VAIQPVN DHAPVQTISR VFHVAR**GGQR LLTTDDVAFS DADSGFSVAQ LVLTRKDLLF GSIVAMEEPT RPIYR**FTQED LR**KKQVLFVH  **1101** SGADHGWLQL QVSDGQHQAT AMLEVQASEP YLHVANSSSL VVPQGGQGTI DTAVLQLDTN LDIR**SGNEVH YHVTAGPQWG QLLRDGQSVT SFSQR**DLLDG  **1201** AILYSHNGSL SPQDTLAFSV AAGPVHTNTF LQVTIALEGP LAPLQLVQHK KIYVFQGEAA EIRR**DQLEVV QEAVLPADIM FSLRSPPNAG YLVMVSHGAS**  **1301 AEEPPSLDPV QSFSQEAVNS GR**VLYLHSRP GAWSDSFSLD VASGLGDPLE GISVELEVLP TVIPLDVQNF SVPEGGTR**TL APPLVQITGP YFPTLPGLVL**  **1401 QVLEPPQHGA LQK**EDHSQDG SLSTFSRR**EV EEQLIR**YVHD GSETQTDAFV LLANASEMDR QSQPVAFTIT ILPVNDQPPV LTTNTGLQIW EGAIVPIPPE  **1501** ALRGTDNDSG PEDLVYTIEE PSNGRIALR**V APDTEVHR**FT QAQLDGGLVL FSHRGALEGG FHFDLSDGAH TSPGHFFRVV AQK**QALLSLE GTRKLTVCPE**  **1601 SVQPLSSQSL SASSSTGADP RHLLYR**VVRG PQLGRLLHAQ QGSAEEVLVN FTQAEVNAGN ILYEHEMSSE PFWEAHDTIG LLLSSPPARD LAATLAVMVS  **1701** FDAACPQRPS RLWK**NKGLWV PEGQRAKITV AALDAANLLA SVPASQRSRH DVLFQVTQFP TRGQLLVSEE PLHARRPYFL QSELAAGQLV YAHGGGGTQQ**  **1801 DGFR**FR**AHLQ GPTGTSVAGP QTSEAFVITV RDVNERPPQP QASIPLR**VTR GSRAPVSR**AQ LSVVDPDSAP GEIEYEVQR**A PHNGFLSLAG DNTGPVTHFT  **1901** QADVDAGRLA FVANGSSVAG VFQLSMSDEA SPPIPMSLAV DVLPSTIEVQ LR**APLEVPQA LGR**TSLSR**QQ LQVISDREEP DVAYR**LTQGP LYGQLLVGGR  **2001** PASAFSQLQV DQGDVVFVFT NFSSSQDHFK VVALARGVNA SATVNVTVQA LLHVWAGGPW PQGTTLR**LDP TVLDASELAN RTGSMPHFR**L LAEPRYGRVV  **2101** RVSQGRTESR **SNQLVEHFTQ RDLEEGQLGL EVGKPEGR**ST GPAGDRLTLE LWAKGVPPAV ALLDFATEPY HAAK**SYSVAL LSVPEAVR**TE TEKPGRSVPT  **2201** GQPGQAASSP VPTAAKGGFL GFLEANMFSI IIPVCLILLL LALILPLLFY LRKRNKTGK**H DVQVLTAKPR NGLAGDTETF R**KVEPGQAIP LITVPGQGPP  **2301** PGGQPDPELL QFCR**TPNPAL RNGQYWV**  **Start - End Observed Mr(expt) Mr(calc) ppm Miss Sequence**  **169 - 179 400.2047 1197.5922 1197.5924 0 0 R.GCLHSAILNGR.N**  Deamidated (NQ) ([Ions score 53](http://10.139.25.109/mascot/cgi/peptide_view.pl?file=../data/20120413/F007793.dat&query=18208&hit=1&index=CSPG4_MOUSE&px=1&section=5&ave_thresh=1&_ignoreionsscorebelow=20&report=0&_sigthreshold=0.05&_msresflags=1089&_msresflags2=2&percolate=-1&percolate_rt=0))  **235 - 248 758.8967 1515.7788 1515.7794 -0 1 R.SQQAPLAFQAGDKR.G**  ([Ions score 84](http://10.139.25.109/mascot/cgi/peptide_view.pl?file=../data/20120413/F007793.dat&query=28379&hit=1&index=CSPG4_MOUSE&px=1&section=5&ave_thresh=1&_ignoreionsscorebelow=20&report=0&_sigthreshold=0.05&_msresflags=1089&_msresflags2=2&percolate=-1&percolate_rt=0))  **297 - 307 660.8442 1319.6738 1319.6721 1 0 R.LEISVDQYPTR.T**  ([Ions score 56](http://10.139.25.109/mascot/cgi/peptide_view.pl?file=../data/20120413/F007793.dat&query=23752&hit=1&index=CSPG4_MOUSE&px=1&section=5&ave_thresh=1&_ignoreionsscorebelow=20&report=0&_sigthreshold=0.05&_msresflags=1089&_msresflags2=2&percolate=-1&percolate_rt=0))  **312 - 320 517.2870 1032.5594 1032.5604 -1 0 R.GVLSYLEPR.G**  ([Ions score 56](http://10.139.25.109/mascot/cgi/peptide_view.pl?file=../data/20120413/F007793.dat&query=10462&hit=1&index=CSPG4_MOUSE&px=1&section=5&ave_thresh=1&_ignoreionsscorebelow=20&report=0&_sigthreshold=0.05&_msresflags=1089&_msresflags2=2&percolate=-1&percolate_rt=0))  **321 - 334 694.8719 1387.7292 1387.7307 -1 0 R.GSLLLGGLDTEASR.H**  ([Ions score 91](http://10.139.25.109/mascot/cgi/peptide_view.pl?file=../data/20120413/F007793.dat&query=25426&hit=1&index=CSPG4_MOUSE&px=1&section=5&ave_thresh=1&_ignoreionsscorebelow=20&report=0&_sigthreshold=0.05&_msresflags=1089&_msresflags2=2&percolate=-1&percolate_rt=0))  **370 - 375 381.2004 760.3862 760.3868 -1 0 R.DAWLTR.D**  ([Ions score 29](http://10.139.25.109/mascot/cgi/peptide_view.pl?file=../data/20120413/F007793.dat&query=1148&hit=1&index=CSPG4_MOUSE&px=1&section=5&ave_thresh=1&_ignoreionsscorebelow=20&report=0&_sigthreshold=0.05&_msresflags=1089&_msresflags2=2&percolate=-1&percolate_rt=0))  **452 - 465 541.2878 1620.8415 1620.8471 -3 0 R.HVQPTLDLTEAELR.K**  ([Ions score 47](http://10.139.25.109/mascot/cgi/peptide_view.pl?file=../data/20120413/F007793.dat&query=31412&hit=1&index=CSPG4_MOUSE&px=1&section=5&ave_thresh=1&_ignoreionsscorebelow=20&report=0&_sigthreshold=0.05&_msresflags=1089&_msresflags2=2&percolate=-1&percolate_rt=0))  **452 - 465 811.4314 1620.8482 1620.8471 1 0 R.HVQPTLDLTEAELR.K**  ([Ions score 97](http://10.139.25.109/mascot/cgi/peptide_view.pl?file=../data/20120413/F007793.dat&query=31413&hit=1&index=CSPG4_MOUSE&px=1&section=5&ave_thresh=1&_ignoreionsscorebelow=20&report=0&_sigthreshold=0.05&_msresflags=1089&_msresflags2=2&percolate=-1&percolate_rt=0))  **452 - 466 583.9855 1748.9348 1748.9421 -4 1 R.HVQPTLDLTEAELRK.S**  ([Ions score 54](http://10.139.25.109/mascot/cgi/peptide_view.pl?file=../data/20120413/F007793.dat&query=34721&hit=1&index=CSPG4_MOUSE&px=1&section=5&ave_thresh=1&_ignoreionsscorebelow=20&report=0&_sigthreshold=0.05&_msresflags=1089&_msresflags2=2&percolate=-1&percolate_rt=0))  **467 - 478 654.8467 1307.6789 1307.6834 -3 0 K.SQVLFSVSQSAR.H**  ([Ions score 58](http://10.139.25.109/mascot/cgi/peptide_view.pl?file=../data/20120413/F007793.dat&query=23220&hit=1&index=CSPG4_MOUSE&px=1&section=5&ave_thresh=1&_ignoreionsscorebelow=20&report=0&_sigthreshold=0.05&_msresflags=1089&_msresflags2=2&percolate=-1&percolate_rt=0))  **479 - 492 513.2707 1536.7902 1536.7896 0 0 R.HGDLELDILGAQTR.K**  ([Ions score 42](http://10.139.25.109/mascot/cgi/peptide_view.pl?file=../data/20120413/F007793.dat&query=28920&hit=1&index=CSPG4_MOUSE&px=1&section=5&ave_thresh=1&_ignoreionsscorebelow=20&report=0&_sigthreshold=0.05&_msresflags=1089&_msresflags2=2&percolate=-1&percolate_rt=0))  **493 - 503 668.3782 1334.7418 1334.7380 3 1 R.KMFTLLDVVNR.K**  ([Ions score 89](http://10.139.25.109/mascot/cgi/peptide_view.pl?file=../data/20120413/F007793.dat&query=24164&hit=1&index=CSPG4_MOUSE&px=1&section=5&ave_thresh=1&_ignoreionsscorebelow=20&report=0&_sigthreshold=0.05&_msresflags=1089&_msresflags2=2&percolate=-1&percolate_rt=0))  **493 - 504 488.6172 1462.8298 1462.8330 -2 2 R.KMFTLLDVVNRK.A**  ([Ions score 38](http://10.139.25.109/mascot/cgi/peptide_view.pl?file=../data/20120413/F007793.dat&query=27182&hit=1&index=CSPG4_MOUSE&px=1&section=5&ave_thresh=1&_ignoreionsscorebelow=20&report=0&_sigthreshold=0.05&_msresflags=1089&_msresflags2=2&percolate=-1&percolate_rt=0))  **494 - 503 604.3304 1206.6463 1206.6431 3 0 K.MFTLLDVVNR.K**  ([Ions score 67](http://10.139.25.109/mascot/cgi/peptide_view.pl?file=../data/20120413/F007793.dat&query=18774&hit=1&index=CSPG4_MOUSE&px=1&section=5&ave_thresh=1&_ignoreionsscorebelow=20&report=0&_sigthreshold=0.05&_msresflags=1089&_msresflags2=2&percolate=-1&percolate_rt=0))  **529 - 536 450.2419 898.4691 898.4695 -0 0 R.APVPSCLR.R**  ([Ions score 36](http://10.139.25.109/mascot/cgi/peptide_view.pl?file=../data/20120413/F007793.dat&query=5001&hit=1&index=CSPG4_MOUSE&px=1&section=5&ave_thresh=1&_ignoreionsscorebelow=20&report=0&_sigthreshold=0.05&_msresflags=1089&_msresflags2=2&percolate=-1&percolate_rt=0))  **537 - 555 730.4111 2188.2099 2188.2117 1 1 R.RGQIYILPIQVNPVNDPPR.I** ([Ions score 63](http://10.139.25.109/mascot/cgi/peptide_view.pl?file=../data/20120413/F007793.dat&query=42481&hit=1&index=CSPG4_MOUSE&px=1&section=5&ave_thresh=1&_ignoreionsscorebelow=20&report=0&_sigthreshold=0.05&_msresflags=1089&_msresflags2=2&percolate=-1&percolate_rt=0))  **538 - 555 678.3768 2032.1085 2032.1106 -1 0 R.GQIYILPIQVNPVNDPPR.I**  ([Ions score 30](http://10.139.25.109/mascot/cgi/peptide_view.pl?file=../data/20120413/F007793.dat&query=40747&hit=1&index=CSPG4_MOUSE&px=1&section=5&ave_thresh=1&_ignoreionsscorebelow=20&report=0&_sigthreshold=0.05&_msresflags=1089&_msresflags2=2&percolate=-1&percolate_rt=0))  **538 - 555 1017.0628 2032.1100 2032.1106 0 0 R.GQIYILPIQVNPVNDPPR.I**  ([Ions score 76](http://10.139.25.109/mascot/cgi/peptide_view.pl?file=../data/20120413/F007793.dat&query=40754&hit=1&index=CSPG4_MOUSE&px=1&section=5&ave_thresh=1&_ignoreionsscorebelow=20&report=0&_sigthreshold=0.05&_msresflags=1089&_msresflags2=2&percolate=-1&percolate_rt=0))  **608 - 620 747.3194 1492.6242 1492.6253 -1 0 R.DQPGEPATEFSCR.E**  ([Ions score 60](http://10.139.25.109/mascot/cgi/peptide_view.pl?file=../data/20120413/F007793.dat&query=27827&hit=1&index=CSPG4_MOUSE&px=1&section=5&ave_thresh=1&_ignoreionsscorebelow=20&report=0&_sigthreshold=0.05&_msresflags=1089&_msresflags2=2&percolate=-1&percolate_rt=0))  **633 - 642 531.2730 1060.5315 1060.5302 1 0 R.GGPAQDLTFR.V**  ([Ions score 61](http://10.139.25.109/mascot/cgi/peptide_view.pl?file=../data/20120413/F007793.dat&query=11637&hit=1&index=CSPG4_MOUSE&px=1&section=5&ave_thresh=1&_ignoreionsscorebelow=20&report=0&_sigthreshold=0.05&_msresflags=1089&_msresflags2=2&percolate=-1&percolate_rt=0))  **643 - 656 688.3481 1374.6817 1374.6813 0 0 R.VSDGMQASAPATLK.V**  ([Ions score 74](http://10.139.25.109/mascot/cgi/peptide_view.pl?file=../data/20120413/F007793.dat&query=25082&hit=1&index=CSPG4_MOUSE&px=1&section=5&ave_thresh=1&_ignoreionsscorebelow=20&report=0&_sigthreshold=0.05&_msresflags=1089&_msresflags2=2&percolate=-1&percolate_rt=0))  **643 - 656 696.3454 1390.6762 1390.6762 0 0 R.VSDGMQASAPATLK.V**  Oxidation (M) ([Ions score 90](http://10.139.25.109/mascot/cgi/peptide_view.pl?file=../data/20120413/F007793.dat&query=25483&hit=1&index=CSPG4_MOUSE&px=1&section=5&ave_thresh=1&_ignoreionsscorebelow=20&report=0&_sigthreshold=0.05&_msresflags=1089&_msresflags2=2&percolate=-1&percolate_rt=0))  **704 - 715 660.8631 1319.7116 1319.7085 2 0 R.VTGTLQFGELQK.Q**  ([Ions score 80](http://10.139.25.109/mascot/cgi/peptide_view.pl?file=../data/20120413/F007793.dat&query=23771&hit=1&index=CSPG4_MOUSE&px=1&section=5&ave_thresh=1&_ignoreionsscorebelow=20&report=0&_sigthreshold=0.05&_msresflags=1089&_msresflags2=2&percolate=-1&percolate_rt=0))  **716 - 735 749.0194 2244.0348 2244.0348 0 0 K.QGAGGVEGTEWWDTLAFHQR.D** ([Ions score 94](http://10.139.25.109/mascot/cgi/peptide_view.pl?file=../data/20120413/F007793.dat&query=42941&hit=1&index=CSPG4_MOUSE&px=1&section=5&ave_thresh=1&_ignoreionsscorebelow=20&report=0&_sigthreshold=0.05&_msresflags=1089&_msresflags2=2&percolate=-1&percolate_rt=0))  **783 - 789 438.7414 875.4682 875.4687 -1 0 R.ATVWMLR.L**  ([Ions score 29](http://10.139.25.109/mascot/cgi/peptide_view.pl?file=../data/20120413/F007793.dat&query=4259&hit=1&index=CSPG4_MOUSE&px=1&section=5&ave_thresh=1&_ignoreionsscorebelow=20&report=0&_sigthreshold=0.05&_msresflags=1089&_msresflags2=2&percolate=-1&percolate_rt=0))  **836 - 845 564.3365 1126.6584 1126.6571 1 1 R.RGNLLLQGTR.L**  ([Ions score 51](http://10.139.25.109/mascot/cgi/peptide_view.pl?file=../data/20120413/F007793.dat&query=14700&hit=1&index=CSPG4_MOUSE&px=1&section=5&ave_thresh=1&_ignoreionsscorebelow=20&report=0&_sigthreshold=0.05&_msresflags=1089&_msresflags2=2&percolate=-1&percolate_rt=0))  **837 - 845 486.2854 970.5563 970.5560 0 0 R.GNLLLQGTR.L**  ([Ions score 61](http://10.139.25.109/mascot/cgi/peptide_view.pl?file=../data/20120413/F007793.dat&query=7849&hit=1&index=CSPG4_MOUSE&px=1&section=5&ave_thresh=1&_ignoreionsscorebelow=20&report=0&_sigthreshold=0.05&_msresflags=1089&_msresflags2=2&percolate=-1&percolate_rt=0))  **846 - 861 848.8901 1695.7656 1695.7700 -3 0 R.LSDGESFSQSDLQAGR.V**  ([Ions score 106](http://10.139.25.109/mascot/cgi/peptide_view.pl?file=../data/20120413/F007793.dat&query=33377&hit=1&index=CSPG4_MOUSE&px=1&section=5&ave_thresh=1&_ignoreionsscorebelow=20&report=0&_sigthreshold=0.05&_msresflags=1089&_msresflags2=2&percolate=-1&percolate_rt=0))  **870 - 879 549.7374 1097.4603 1097.4625 -2 0 R.TSEAADDSFR.F**  ([Ions score 67](http://10.139.25.109/mascot/cgi/peptide_view.pl?file=../data/20120413/F007793.dat&query=13255&hit=1&index=CSPG4_MOUSE&px=1&section=5&ave_thresh=1&_ignoreionsscorebelow=20&report=0&_sigthreshold=0.05&_msresflags=1089&_msresflags2=2&percolate=-1&percolate_rt=0))  **931 - 949 548.2629 2189.0203 2189.0212 0 0 K.SLNSASYLYEVMEQPHHGK.L** ([Ions score 43](http://10.139.25.109/mascot/cgi/peptide_view.pl?file=../data/20120413/F007793.dat&query=42482&hit=1&index=CSPG4_MOUSE&px=1&section=5&ave_thresh=1&_ignoreionsscorebelow=20&report=0&_sigthreshold=0.05&_msresflags=1089&_msresflags2=2&percolate=-1&percolate_rt=0))  **931 - 949 552.2555 2204.9907 2204.9922 -1 0 K.SLNSASYLYEVMEQPHHGK.L**  Oxidation (M) ([Ions score 45](http://10.139.25.109/mascot/cgi/peptide_view.pl?file=../data/20120413/F007793.dat&query=42600&hit=1&index=CSPG4_MOUSE&px=1&section=5&ave_thresh=1&_ignoreionsscorebelow=20&report=0&_sigthreshold=0.05&_msresflags=1089&_msresflags2=2&percolate=-1&percolate_rt=0))  **959 - 974 591.9640 1772.8686 1772.8693 0 0 K.STPVTSFTNEDLLHGR.L**  ([Ions score 45](http://10.139.25.109/mascot/cgi/peptide_view.pl?file=../data/20120413/F007793.dat&query=35360&hit=1&index=CSPG4_MOUSE&px=1&section=5&ave_thresh=1&_ignoreionsscorebelow=20&report=0&_sigthreshold=0.05&_msresflags=1089&_msresflags2=2&percolate=-1&percolate_rt=0))  **975 - 995 821.7276 2462.1594 2462.1601 0 0 R.LVYQHDDSETIEDDIPFVATR.Q**  ([Ions score 59](http://10.139.25.109/mascot/cgi/peptide_view.pl?file=../data/20120413/F007793.dat&query=44306&hit=1&index=CSPG4_MOUSE&px=1&section=5&ave_thresh=1&_ignoreionsscorebelow=20&report=0&_sigthreshold=0.05&_msresflags=1089&_msresflags2=2&percolate=-1&percolate_rt=0))  **1014 - 1030 615.6710 1843.9896 1843.9904 0 0 R.VAIQPVNDHAPVQTISR.V** ([Ions score 43](http://10.139.25.109/mascot/cgi/peptide_view.pl?file=../data/20120413/F007793.dat&query=37326&hit=1&index=CSPG4_MOUSE&px=1&section=5&ave_thresh=1&_ignoreionsscorebelow=20&report=0&_sigthreshold=0.05&_msresflags=1089&_msresflags2=2&percolate=-1&percolate_rt=0))  **1014 - 1030 923.0030 1843.9904 1843.9904 0 0 R.VAIQPVNDHAPVQTISR.V** ([Ions score 68](http://10.139.25.109/mascot/cgi/peptide_view.pl?file=../data/20120413/F007793.dat&query=37328&hit=1&index=CSPG4_MOUSE&px=1&section=5&ave_thresh=1&_ignoreionsscorebelow=20&report=0&_sigthreshold=0.05&_msresflags=1089&_msresflags2=2&percolate=-1&percolate_rt=0))  **1031 - 1036 364.7138 727.4130 727.4129 0 0 R.VFHVAR.G**  ([Ions score 33](http://10.139.25.109/mascot/cgi/peptide_view.pl?file=../data/20120413/F007793.dat&query=436&hit=1&index=CSPG4_MOUSE&px=1&section=5&ave_thresh=1&_ignoreionsscorebelow=20&report=0&_sigthreshold=0.05&_msresflags=1089&_msresflags2=2&percolate=-1&percolate_rt=0))  **1086 - 1092 454.7272 907.4398 907.4399 0 0 R.FTQEDLR.K**  ([Ions score 47](http://10.139.25.109/mascot/cgi/peptide_view.pl?file=../data/20120413/F007793.dat&query=5367&hit=1&index=CSPG4_MOUSE&px=1&section=5&ave_thresh=1&_ignoreionsscorebelow=20&report=0&_sigthreshold=0.05&_msresflags=1089&_msresflags2=2&percolate=-1&percolate_rt=0))  **1165 - 1184 750.3791 2248.1156 2248.1138 1 0 R.SGNEVHYHVTAGPQWGQLLR.D**  ([Ions score 59](http://10.139.25.109/mascot/cgi/peptide_view.pl?file=../data/20120413/F007793.dat&query=42954&hit=1&index=CSPG4_MOUSE&px=1&section=5&ave_thresh=1&_ignoreionsscorebelow=20&report=0&_sigthreshold=0.05&_msresflags=1089&_msresflags2=2&percolate=-1&percolate_rt=0))  **1165 - 1184 563.0364 2248.1143 2248.1138 0 0 R.SGNEVHYHVTAGPQWGQLLR.D**  ([Ions score 42](http://10.139.25.109/mascot/cgi/peptide_view.pl?file=../data/20120413/F007793.dat&query=42960&hit=1&index=CSPG4_MOUSE&px=1&section=5&ave_thresh=1&_ignoreionsscorebelow=20&report=0&_sigthreshold=0.05&_msresflags=1089&_msresflags2=2&percolate=-1&percolate_rt=0))  **1185 - 1195 606.2859 1210.5572 1210.5578 -1 0 R.DGQSVTSFSQR.D**  ([Ions score 75](http://10.139.25.109/mascot/cgi/peptide_view.pl?file=../data/20120413/F007793.dat&query=18925&hit=1&index=CSPG4_MOUSE&px=1&section=5&ave_thresh=1&_ignoreionsscorebelow=20&report=0&_sigthreshold=0.05&_msresflags=1089&_msresflags2=2&percolate=-1&percolate_rt=0))  **1265 - 1284 758.4007 2272.1787 2272.1773 1 0 R.DQLEVVQEAVLPADIMFSLR.S** ([Ions score 55](http://10.139.25.109/mascot/cgi/peptide_view.pl?file=../data/20120413/F007793.dat&query=43105&hit=1&index=CSPG4_MOUSE&px=1&section=5&ave_thresh=1&_ignoreionsscorebelow=20&report=0&_sigthreshold=0.05&_msresflags=1089&_msresflags2=2&percolate=-1&percolate_rt=0))  **1285 - 1322 978.7188 3910.8439 3910.8432 0 0 R.SPPNAGYLVMVSHGASAEEPPSLDPVQSFSQEAVNSGR.V** ([Ions score 31](http://10.139.25.109/mascot/cgi/peptide_view.pl?file=../data/20120413/F007793.dat&query=48544&hit=1&index=CSPG4_MOUSE&px=1&section=5&ave_thresh=1&_ignoreionsscorebelow=20&report=0&_sigthreshold=0.05&_msresflags=1089&_msresflags2=2&percolate=-1&percolate_rt=0))  **1379 – 1413 1245.0408 3732.0990 3732.1018 1 0 R.TLAPPLVQITGPYFPTLPGLVLQVLEPPQHGALQK.E** ([Ions score 49](http://10.139.25.109/mascot/cgi/peptide_view.pl?file=../data/20120413/F007793.dat&query=48472&hit=1&index=CSPG4_MOUSE&px=1&section=5&ave_thresh=1&_ignoreionsscorebelow=20&report=0&_sigthreshold=0.05&_msresflags=1089&_msresflags2=2&percolate=-1&percolate_rt=0))  **1379 - 1413 934.0333 3732.1019 3732.1018 0 0 R.TLAPPLVQITGPYFPTLPGLVLQVLEPPQHGALQK.E** ([Ions score 22](http://10.139.25.109/mascot/cgi/peptide_view.pl?file=../data/20120413/F007793.dat&query=48473&hit=1&index=CSPG4_MOUSE&px=1&section=5&ave_thresh=1&_ignoreionsscorebelow=20&report=0&_sigthreshold=0.05&_msresflags=1089&_msresflags2=2&percolate=-1&percolate_rt=0))  **1429 - 1436 508.2741 1014.5336 1014.5345 -1 0 R.EVEEQLIR.Y**  ([Ions score 43](http://10.139.25.109/mascot/cgi/peptide_view.pl?file=../data/20120413/F007793.dat&query=9611&hit=1&index=CSPG4_MOUSE&px=1&section=5&ave_thresh=1&_ignoreionsscorebelow=20&report=0&_sigthreshold=0.05&_msresflags=1089&_msresflags2=2&percolate=-1&percolate_rt=0))  **1530 - 1538 512.2637 1022.5129 1022.5145 -2 0 R.VAPDTEVHR.F**  ([Ions score 39](http://10.139.25.109/mascot/cgi/peptide_view.pl?file=../data/20120413/F007793.dat&query=9973&hit=1&index=CSPG4_MOUSE&px=1&section=5&ave_thresh=1&_ignoreionsscorebelow=20&report=0&_sigthreshold=0.05&_msresflags=1089&_msresflags2=2&percolate=-1&percolate_rt=0))  **1584 - 1593 544.3097 1086.6048 1086.6033 1 0 K.QALLSLEGTR.K**  ([Ions score 66](http://10.139.25.109/mascot/cgi/peptide_view.pl?file=../data/20120413/F007793.dat&query=12791&hit=1&index=CSPG4_MOUSE&px=1&section=5&ave_thresh=1&_ignoreionsscorebelow=20&report=0&_sigthreshold=0.05&_msresflags=1089&_msresflags2=2&percolate=-1&percolate_rt=0))  **1584 - 1594 608.3571 1214.6986 1214.6983 0 1 K.QALLSLEGTRK.L**  ([Ions score 28](http://10.139.25.109/mascot/cgi/peptide_view.pl?file=../data/20120413/F007793.dat&query=19199&hit=1&index=CSPG4_MOUSE&px=1&section=5&ave_thresh=1&_ignoreionsscorebelow=20&report=0&_sigthreshold=0.05&_msresflags=1089&_msresflags2=2&percolate=-1&percolate_rt=0))  **1594 - 1621 963.8141 2888.4189 2888.4186 0 1 R.KLTVCPESVQPLSSQSLSASSSTGADPR.H** ([Ions score 53](http://10.139.25.109/mascot/cgi/peptide_view.pl?file=../data/20120413/F007793.dat&query=46790&hit=1&index=CSPG4_MOUSE&px=1&section=5&ave_thresh=1&_ignoreionsscorebelow=20&report=0&_sigthreshold=0.05&_msresflags=1089&_msresflags2=2&percolate=-1&percolate_rt=0))  **1622 – 1626 351.2080 700.4015 700.4020 -1 0 R.HLLYR.V**  ([Ions score 24](http://10.139.25.109/mascot/cgi/peptide_view.pl?file=../data/20120413/F007793.dat&query=13&hit=1&index=CSPG4_MOUSE&px=1&section=5&ave_thresh=1&_ignoreionsscorebelow=20&report=0&_sigthreshold=0.05&_msresflags=1089&_msresflags2=2&percolate=-1&percolate_rt=0))  **1715 - 1725 642.3460 1282.6773 1282.6782 -1 1 K.NKGLWVPEGQR.A**  ([Ions score 39](http://10.139.25.109/mascot/cgi/peptide_view.pl?file=../data/20120413/F007793.dat&query=22314&hit=1&index=CSPG4_MOUSE&px=1&section=5&ave_thresh=1&_ignoreionsscorebelow=20&report=0&_sigthreshold=0.05&_msresflags=1089&_msresflags2=2&percolate=-1&percolate_rt=0))  **1717 - 1725 521.2781 1040.5417 1040.5403 1 0 K.GLWVPEGQR.A**  ([Ions score 24](http://10.139.25.109/mascot/cgi/peptide_view.pl?file=../data/20120413/F007793.dat&query=10820&hit=1&index=CSPG4_MOUSE&px=1&section=5&ave_thresh=1&_ignoreionsscorebelow=20&report=0&_sigthreshold=0.05&_msresflags=1089&_msresflags2=2&percolate=-1&percolate_rt=0))  **1726 - 1747 727.4189 2179.2333 2179.2325 0 1 R.AKITVAALDAANLLASVPASQR.S** ([Ions score 32](http://10.139.25.109/mascot/cgi/peptide_view.pl?file=../data/20120413/F007793.dat&query=42400&hit=1&index=CSPG4_MOUSE&px=1&section=5&ave_thresh=1&_ignoreionsscorebelow=20&report=0&_sigthreshold=0.05&_msresflags=1089&_msresflags2=2&percolate=-1&percolate_rt=0))  **1728 - 1747 661.0412 1980.1002 1980.1004 0 0 K.ITVAALDAANLLASVPASQR.S** ([Ions score 81](http://10.139.25.109/mascot/cgi/peptide_view.pl?file=../data/20120413/F007793.dat&query=39970&hit=1&index=CSPG4_MOUSE&px=1&section=5&ave_thresh=1&_ignoreionsscorebelow=20&report=0&_sigthreshold=0.05&_msresflags=1089&_msresflags2=2&percolate=-1&percolate_rt=0))  **1748 - 1762 915.9830 1829.9515 1829.9537 -1 1 R.SRHDVLFQVTQFPTR.G**  ([Ions score 79](http://10.139.25.109/mascot/cgi/peptide_view.pl?file=../data/20120413/F007793.dat&query=36951&hit=1&index=CSPG4_MOUSE&px=1&section=5&ave_thresh=1&_ignoreionsscorebelow=20&report=0&_sigthreshold=0.05&_msresflags=1089&_msresflags2=2&percolate=-1&percolate_rt=0))  **1748 - 1762 610.9917 1829.9533 1829.9537 0 1 R.SRHDVLFQVTQFPTR.G**  ([Ions score 77](http://10.139.25.109/mascot/cgi/peptide_view.pl?file=../data/20120413/F007793.dat&query=36954&hit=1&index=CSPG4_MOUSE&px=1&section=5&ave_thresh=1&_ignoreionsscorebelow=20&report=0&_sigthreshold=0.05&_msresflags=1089&_msresflags2=2&percolate=-1&percolate_rt=0))  **1748 - 1762 458.4961 1829.9531 1829.9537 0 1 R.SRHDVLFQVTQFPTR.G**  ([Ions score 25](http://10.139.25.109/mascot/cgi/peptide_view.pl?file=../data/20120413/F007793.dat&query=36985&hit=1&index=CSPG4_MOUSE&px=1&section=5&ave_thresh=1&_ignoreionsscorebelow=20&report=0&_sigthreshold=0.05&_msresflags=1089&_msresflags2=2&percolate=-1&percolate_rt=0))  **1763 - 1775 483.6000 1447.7781 1447.7783 0 0 R.GQLLVSEEPLHAR.R**  ([Ions score 48](http://10.139.25.109/mascot/cgi/peptide_view.pl?file=../data/20120413/F007793.dat&query=26789&hit=1&index=CSPG4_MOUSE&px=1&section=5&ave_thresh=1&_ignoreionsscorebelow=20&report=0&_sigthreshold=0.05&_msresflags=1089&_msresflags2=2&percolate=-1&percolate_rt=0))  **1776 - 1804 781.6416 3122.5351 3122.5322 1 0 R.RPYFLQSELAAGQLVYAHGGGGTQQDGFR.F** ([Ions score 91](http://10.139.25.109/mascot/cgi/peptide_view.pl?file=../data/20120413/F007793.dat&query=47488&hit=1&index=CSPG4_MOUSE&px=1&section=5&ave_thresh=1&_ignoreionsscorebelow=20&report=0&_sigthreshold=0.05&_msresflags=1089&_msresflags2=2&percolate=-1&percolate_rt=0))  **1807 - 1831 842.1067 2523.2967 2523.3082 4 0 R.AHLQGPTGTSVAGPQTSEAFVITVR.D** ([Ions score 80](http://10.139.25.109/mascot/cgi/peptide_view.pl?file=../data/20120413/F007793.dat&query=44772&hit=1&index=CSPG4_MOUSE&px=1&section=5&ave_thresh=1&_ignoreionsscorebelow=20&report=0&_sigthreshold=0.05&_msresflags=1089&_msresflags2=2&percolate=-1&percolate_rt=0))  **1832 - 1847 606.3273 1815.9600 1815.9591 0 0 R.DVNERPPQPQASIPLR.V**  ([Ions score 46](http://10.139.25.109/mascot/cgi/peptide_view.pl?file=../data/20120413/F007793.dat&query=36552&hit=1&index=CSPG4_MOUSE&px=1&section=5&ave_thresh=1&_ignoreionsscorebelow=20&report=0&_sigthreshold=0.05&_msresflags=1089&_msresflags2=2&percolate=-1&percolate_rt=0))  **1832 - 1847 908.9900 1815.9654 1815.9591 3 0 R.DVNERPPQPQASIPLR.V**  ([Ions score 71](http://10.139.25.109/mascot/cgi/peptide_view.pl?file=../data/20120413/F007793.dat&query=36556&hit=1&index=CSPG4_MOUSE&px=1&section=5&ave_thresh=1&_ignoreionsscorebelow=20&report=0&_sigthreshold=0.05&_msresflags=1089&_msresflags2=2&percolate=-1&percolate_rt=0))  **1859 - 1879 1151.5637 2301.1118 2301.1125 0 0 R.AQLSVVDPDSAPGEIEYEVQR.A** ([Ions score 96](http://10.139.25.109/mascot/cgi/peptide_view.pl?file=../data/20120413/F007793.dat&query=43333&hit=1&index=CSPG4_MOUSE&px=1&section=5&ave_thresh=1&_ignoreionsscorebelow=20&report=0&_sigthreshold=0.05&_msresflags=1089&_msresflags2=2&percolate=-1&percolate_rt=0))  **1859 - 1879 768.0453 2301.1125 2301.1125 0 0 R.AQLSVVDPDSAPGEIEYEVQR.A** ([Ions score 62](http://10.139.25.109/mascot/cgi/peptide_view.pl?file=../data/20120413/F007793.dat&query=43334&hit=1&index=CSPG4_MOUSE&px=1&section=5&ave_thresh=1&_ignoreionsscorebelow=20&report=0&_sigthreshold=0.05&_msresflags=1089&_msresflags2=2&percolate=-1&percolate_rt=0))  **1953 - 1963 575.8321 1149.6496 1149.6506 -1 0 R.APLEVPQALGR.T**  ([Ions score 40](http://10.139.25.109/mascot/cgi/peptide_view.pl?file=../data/20120413/F007793.dat&query=15924&hit=1&index=CSPG4_MOUSE&px=1&section=5&ave_thresh=1&_ignoreionsscorebelow=20&report=0&_sigthreshold=0.05&_msresflags=1089&_msresflags2=2&percolate=-1&percolate_rt=0))  **1969 - 1985 1014.9917 2027.9678 2027.9674 0 1 R.QQLQVISDREEPDVAYR.L**  Gln->pyro-Glu(N-term Q)([Ions score 53](http://10.139.25.109/mascot/cgi/peptide_view.pl?file=../data/20120413/F007793.dat&query=40709&hit=1&index=CSPG4_MOUSE&px=1&section=5&ave_thresh=1&_ignoreionsscorebelow=20&report=0&_sigthreshold=0.05&_msresflags=1089&_msresflags2=2&percolate=-1&percolate_rt=0))  **1969 - 1985 682.6809 2045.0193 2045.0178 1 1 R.QQLQVISDREEPDVAYR.L** ([Ions score 43](http://10.139.25.109/mascot/cgi/peptide_view.pl?file=../data/20120413/F007793.dat&query=40930&hit=1&index=CSPG4_MOUSE&px=1&section=5&ave_thresh=1&_ignoreionsscorebelow=20&report=0&_sigthreshold=0.05&_msresflags=1089&_msresflags2=2&percolate=-1&percolate_rt=0))  **1969 - 1985 1023.5163 2045.0170 2045.0178 0 1 R.QQLQVISDREEPDVAYR.L**  ([Ions score 53](http://10.139.25.109/mascot/cgi/peptide_view.pl?file=../data/20120413/F007793.dat&query=40931&hit=1&index=CSPG4_MOUSE&px=1&section=5&ave_thresh=1&_ignoreionsscorebelow=20&report=0&_sigthreshold=0.05&_msresflags=1089&_msresflags2=2&percolate=-1&percolate_rt=0))  **2068 - 2081 757.3966 1512.7776 1512.7784 1 0 R.LDPTVLDASELANR.T** ([Ions score 23](http://10.139.25.109/mascot/cgi/peptide_view.pl?file=../data/20120413/F007793.dat&query=28264&hit=1&index=CSPG4_MOUSE&px=1&section=5&ave_thresh=1&_ignoreionsscorebelow=20&report=0&_sigthreshold=0.05&_msresflags=1089&_msresflags2=2&percolate=-1&percolate_rt=0))  **2082 - 2089 466.7247 931.4338 931.4334 0 0 R.TGSMPHFR.L**  ([Ions score 32](http://10.139.25.109/mascot/cgi/peptide_view.pl?file=../data/20120413/F007793.dat&query=6331&hit=1&index=CSPG4_MOUSE&px=1&section=5&ave_thresh=1&_ignoreionsscorebelow=20&report=0&_sigthreshold=0.05&_msresflags=1089&_msresflags2=2&percolate=-1&percolate_rt=0))  **2111 - 2121 453.5652 1357.6738 1357.6739 0 0 R.SNQLVEHFTQR.D**  ([Ions score 43](http://10.139.25.109/mascot/cgi/peptide_view.pl?file=../data/20120413/F007793.dat&query=24737&hit=1&index=CSPG4_MOUSE&px=1&section=5&ave_thresh=1&_ignoreionsscorebelow=20&report=0&_sigthreshold=0.05&_msresflags=1089&_msresflags2=2&percolate=-1&percolate_rt=0))  **2122 - 2138 913.4690 1824.9224 1824.9217 0 0 R.DLEEGQLGLEVGKPEGR.S** ([Ions score 62](http://10.139.25.109/mascot/cgi/peptide_view.pl?file=../data/20120413/F007793.dat&query=36792&hit=1&index=CSPG4_MOUSE&px=1&section=5&ave_thresh=1&_ignoreionsscorebelow=20&report=0&_sigthreshold=0.05&_msresflags=1089&_msresflags2=2&percolate=-1&percolate_rt=0))  **2122 - 2138 609.3147 1824.9207 1824.9218 1 0 R.DLEEGQLGLEVGKPEGR.S** ([Ions score 58](http://10.139.25.109/mascot/cgi/peptide_view.pl?file=../data/20120413/F007793.dat&query=36841&hit=1&index=CSPG4_MOUSE&px=1&section=5&ave_thresh=1&_ignoreionsscorebelow=20&report=0&_sigthreshold=0.05&_msresflags=1089&_msresflags2=2&percolate=-1&percolate_rt=0))  **2175 - 2188 745.9143 1489.8139 1489.8140 0 0 K.SYSVALLSVPEAVR.T**  ([Ions score 59](http://10.139.25.109/mascot/cgi/peptide_view.pl?file=../data/20120413/F007793.dat&query=27783&hit=1&index=CSPG4_MOUSE&px=1&section=5&ave_thresh=1&_ignoreionsscorebelow=20&report=0&_sigthreshold=0.05&_msresflags=1089&_msresflags2=2&percolate=-1&percolate_rt=0))  **2260 - 2270 421.9110 1262.7111 1262.7095 1 0 K.HDVQVLTAKPR.N**  ([Ions score 43](http://10.139.25.109/mascot/cgi/peptide_view.pl?file=../data/20120413/F007793.dat&query=21441&hit=1&index=CSPG4_MOUSE&px=1&section=5&ave_thresh=1&_ignoreionsscorebelow=20&report=0&_sigthreshold=0.05&_msresflags=1089&_msresflags2=2&percolate=-1&percolate_rt=0))  **2271 - 2281 591.2785 1180.5424 1180.5360 5 0 R.NGLAGDTETFR.K**  Deamidated (NQ) ([Ions score 31](http://10.139.25.109/mascot/cgi/peptide_view.pl?file=../data/20120413/F007793.dat&query=17296&hit=1&index=CSPG4_MOUSE&px=1&section=5&ave_thresh=1&_ignoreionsscorebelow=20&report=0&_sigthreshold=0.05&_msresflags=1089&_msresflags2=2&percolate=-1&percolate_rt=0))  **2315 - 2327 758.8814 1515.7483 1515.7470 1 1 R.TPNPALRNGQYWV.-** Deamidated (NQ) ([Ions score 30](http://10.139.25.109/mascot/cgi/peptide_view.pl?file=../data/20120413/F007793.dat&query=28373&hit=1&index=CSPG4_MOUSE&px=1&section=5&ave_thresh=1&_ignoreionsscorebelow=20&report=0&_sigthreshold=0.05&_msresflags=1089&_msresflags2=2&percolate=-1&percolate_rt=0))  3. [D3YWD1_MOUSE](http://10.139.25.109/mascot/cgi/protein_view.pl?file=../data/20120413/F007793.dat&hit=D3YWD1_MOUSE&db_idx=1&px=1&ave_thresh=1&_ignoreionsscorebelow=20&report=0&_sigthreshold=0.05&_msresflags=1089&_msresflags2=2&percolate=-1&percolate_rt=0)    **Mass:** 163838   **Score:** 2491   **Matches:** 44(44)  **Sequences:** 38(38)  Collagen, type VI, alpha 3 (Fragment)  Sequence Coverage: **35%**; Matched peptides shown in **Bold Red**  **1** MRKHRHLPLV AVFSLLLSGI ATTHAQQHGD VKNGAAADIV FLVDSSWSAG KDRFLLVQEF LSDVVESLAV GDNDFHFALV RLNGNPHTEF LLNTYHSKQE  **101** VLSHIVNMSY IGGSNQTGKG LEYVIHSHLT EASGSRAADG VPQVIIVLTD GQSEDGFALP SAELKSADVN VFAVGVEGAD ERALGEVASE PLSMHVFNLE  **201** NVTSLHGLVG NLVSCIHSSV NPERAGDKES LKDITAQDSA DIIFLIDGSQ NTGNANFDVI RDFLVNVLER LSVGNQQVQV GVVQYSEEPI TMFSLNSYPS  **301** KAAVLDAVKG LSLVGGESAN IGQALDFVVE NHFTRAGGSR VEEGVPQVLV LISAGPSSDE IRDSVVALKQ ASVFSFGLGA QAASRAELQH IATDDSLVFT  **401** VPEFR**SFGDL QEQILPYLVG VAQR**HIVLQP PAIVTQVMEV NKRDIVFLVD GSSSLGPSNF NAIRDFVTRV IQRLEIGQDL VQVSVAQYAD TVKPEFYLNS  **501** YTNKRDAITA VRKMRALNGS ALYTGSSLDF VRNNLFTSSA GHRAAEGVPK **LLVLITGGKS LDEVSQPAQE LKRGSIMALA VGSK**AADEDE LKEIAFDSSL  **601** VFIPAEFRPA PLQNMLPSLM APLRTLTGTT EESKRDILFL FDGSVNVLGQ FPAVRDFLYR **IIEELDVKPD GTRVAIAQFS DDVR**LESRFS EHQTK**AEILN**  **701 LVK**KMKLKTG K**ALNLGYALD YALR**NIFVRS AGSR**IEDNVQ QFLVLLVAGR SSDAVAGPAS SLK**QRGVVPF IFQAK**NANPS ELEQIVLSPA FILAAESLPK**  **801 IGDLQSQIVS LLK**AEQGSGP VSGEKDVVFL IDGSEGVR**SG FPLLK**DFVQR **VVESLDVGPD R**VR**VALVQYS DR**TRPEFYLN SHMDQQGVIS AIRR**LTLLGG**  **901 PTPNTGAALE FVLRNILTSS TGSRIAEGVP QLLIVLTAEP SGDDVRGPSV VLK**QGGAVPI GIGIGNADIS EMQTISFIPD FAVAIPTFR**E LGTIQQVISE**  **1001 RVIQLNREEL SSLKPILTPS TGAGVGSK**K**D VVFLIDGSRN AGPEFQYIR**T LIERIVEYLD IGFDTTRVAV IQFSEDSKME FPLNAHFSKD EVQNAVRRLR  **1101** PK**GGSQVYIG NALEYVLK**NI FQRPLGSR**IE EGVPQFLVLI SSGK**SDDEVD DSAVELK**QFG VAPLTIAR**HT DQEELVK**ISL SPEYVYSVST FR**ELPRLEQK  **1201** **LLTPITTLTS QQIHQILAST R**YPPSVVESD AADIVFLIDS SDAVKPDGIA HIRDFVSRIV RR**LNIGPSK**V R**IGVVQFSND VFPEFYLK**TH K**SQSSVLEAI**  **1301 R**RLRFKGGSP LNTGR**ALEFV AR**NLFVKSAG SR**IEDGVPQH LVLFLGGK**SQ DDVARHAQVI SSSGIVSLGI GDR**NIDRTDL QTITNDPRLV FTVR**EFRELP  **1401** NIEER**VMLSF GPSGATPQPP GVDLPSPSRP EK**KKADIVFL LDGSINFRR**D SFQEVLRFAS EIVDTVYEDG DSIRVGLVQY NSDPTDEFFL R**DFSTKRQII  **1501** DAI  **Start - End Observed Mr(expt) Mr(calc) ppm Miss Sequence**  **406 - 424 711.7155 2132.1245 2132.1266 -1 0 R.SFGDLQEQILPYLVGVAQR.H**  ([Ions score 63](http://10.139.25.109/mascot/cgi/peptide_view.pl?file=../data/20120413/F007793.dat&query=41993&hit=1&index=D3YWD1_MOUSE&px=1&section=5&ave_thresh=1&_ignoreionsscorebelow=20&report=0&_sigthreshold=0.05&_msresflags=1089&_msresflags2=2&percolate=-1&percolate_rt=0))  **406 - 424 1067.0723 2133.1290 2132.1266 1 0 R.SFGDLQEQILPYLVGVAQR.H**  ([Ions score 103](http://10.139.25.109/mascot/cgi/peptide_view.pl?file=../data/20120413/F007793.dat&query=42000&hit=1&index=D3YWD1_MOUSE&px=1&section=5&ave_thresh=1&_ignoreionsscorebelow=20&report=0&_sigthreshold=0.05&_msresflags=1089&_msresflags2=2&percolate=-1&percolate_rt=0))  **551 - 559 457.3077 912.6009 912.6008 0 0 K.LLVLITGGK.S**  ([Ions score 60](http://10.139.25.109/mascot/cgi/peptide_view.pl?file=../data/20120413/F007793.dat&query=5609&hit=1&index=D3YWD1_MOUSE&px=1&section=5&ave_thresh=1&_ignoreionsscorebelow=20&report=0&_sigthreshold=0.05&_msresflags=1089&_msresflags2=2&percolate=-1&percolate_rt=0))  **560 - 573 800.4211 1598.8277 1598.8264 1 1 K.SLDEVSQPAQELKR.G**  ([Ions score 62](http://10.139.25.109/mascot/cgi/peptide_view.pl?file=../data/20120413/F007793.dat&query=30744&hit=1&index=D3YWD1_MOUSE&px=1&section=5&ave_thresh=1&_ignoreionsscorebelow=20&report=0&_sigthreshold=0.05&_msresflags=1089&_msresflags2=2&percolate=-1&percolate_rt=0))  **574 - 584 517.2880 1032.5615 1032.5638 2 0 R.GSIMALAVGSK.A**  ([Ions score 48](http://10.139.25.109/mascot/cgi/peptide_view.pl?file=../data/20120413/F007793.dat&query=10464&hit=1&index=D3YWD1_MOUSE&px=1&section=5&ave_thresh=1&_ignoreionsscorebelow=20&report=0&_sigthreshold=0.05&_msresflags=1089&_msresflags2=2&percolate=-1&percolate_rt=0))  **661 - 673 495.6031 1483.7875 1483.7882 0 0 R.IIEELDVKPDGTR.V**  ([Ions score 34](http://10.139.25.109/mascot/cgi/peptide_view.pl?file=../data/20120413/F007793.dat&query=27593&hit=1&index=D3YWD1_MOUSE&px=1&section=5&ave_thresh=1&_ignoreionsscorebelow=20&report=0&_sigthreshold=0.05&_msresflags=1089&_msresflags2=2&percolate=-1&percolate_rt=0))  **674 - 684 610.8170 1219.6194 1219.6197 0 0 R.VAIAQFSDDVR.L**  ([Ions score 98](http://10.139.25.109/mascot/cgi/peptide_view.pl?file=../data/20120413/F007793.dat&query=19411&hit=1&index=D3YWD1_MOUSE&px=1&section=5&ave_thresh=1&_ignoreionsscorebelow=20&report=0&_sigthreshold=0.05&_msresflags=1089&_msresflags2=2&percolate=-1&percolate_rt=0))  **696 - 703 450.2814 898.5482 898.5487 -1 0 K.AEILNLVK.K**  ([Ions score 55](http://10.139.25.109/mascot/cgi/peptide_view.pl?file=../data/20120413/F007793.dat&query=5027&hit=1&index=D3YWD1_MOUSE&px=1&section=5&ave_thresh=1&_ignoreionsscorebelow=20&report=0&_sigthreshold=0.05&_msresflags=1089&_msresflags2=2&percolate=-1&percolate_rt=0))  **712 - 724 726.8957 1451.7768 1451.7772 0 0 K.ALNLGYALDYALR.N**  ([Ions score 93](http://10.139.25.109/mascot/cgi/peptide_view.pl?file=../data/20120413/F007793.dat&query=26875&hit=1&index=D3YWD1_MOUSE&px=1&section=5&ave_thresh=1&_ignoreionsscorebelow=20&report=0&_sigthreshold=0.05&_msresflags=1089&_msresflags2=2&percolate=-1&percolate_rt=0))  **735 - 750 605.3445 1813.0116 1813.0098 1 0 R.IEDNVQQFLVLLVAGR.S**  ([Ions score 63](http://10.139.25.109/mascot/cgi/peptide_view.pl?file=../data/20120413/F007793.dat&query=36482&hit=1&index=D3YWD1_MOUSE&px=1&section=5&ave_thresh=1&_ignoreionsscorebelow=20&report=0&_sigthreshold=0.05&_msresflags=1089&_msresflags2=2&percolate=-1&percolate_rt=0))  **735 - 750 907.5129 1814.0102 1813.0098 0 0 R.IEDNVQQFLVLLVAGR.S** ([Ions score 77](http://10.139.25.109/mascot/cgi/peptide_view.pl?file=../data/20120413/F007793.dat&query=36492&hit=1&index=D3YWD1_MOUSE&px=1&section=5&ave_thresh=1&_ignoreionsscorebelow=20&report=0&_sigthreshold=0.05&_msresflags=1089&_msresflags2=2&percolate=-1&percolate_rt=0))  **751 - 763 595.3060 1188.5975 1188.5986 -1 0 R.SSDAVAGPASSLK.Q**  ([Ions score 34](http://10.139.25.109/mascot/cgi/peptide_view.pl?file=../data/20120413/F007793.dat&query=17783&hit=1&index=D3YWD1_MOUSE&px=1&section=5&ave_thresh=1&_ignoreionsscorebelow=20&report=0&_sigthreshold=0.05&_msresflags=1089&_msresflags2=2&percolate=-1&percolate_rt=0))  **776 - 800 884.4819 2650.4223 2650.4218 0 0 K.NANPSELEQIVLSPAFILAAESLPK.I** ([Ions score 60](http://10.139.25.109/mascot/cgi/peptide_view.pl?file=../data/20120413/F007793.dat&query=45694&hit=1&index=D3YWD1_MOUSE&px=1&section=5&ave_thresh=1&_ignoreionsscorebelow=20&report=0&_sigthreshold=0.05&_msresflags=1089&_msresflags2=2&percolate=-1&percolate_rt=0))  **776 - 800 1326.2185 2650.4214 2650.4218 0 0 K.NANPSELEQIVLSPAFILAAESLPK.I** ([Ions score 55](http://10.139.25.109/mascot/cgi/peptide_view.pl?file=../data/20120413/F007793.dat&query=45697&hit=1&index=D3YWD1_MOUSE&px=1&section=5&ave_thresh=1&_ignoreionsscorebelow=20&report=0&_sigthreshold=0.05&_msresflags=1089&_msresflags2=2&percolate=-1&percolate_rt=0))  **801 - 813 707.4198 1412.8250 1412.8239 1 0 K.IGDLQSQIVSLLK.A**  ([Ions score 90](http://10.139.25.109/mascot/cgi/peptide_view.pl?file=../data/20120413/F007793.dat&query=26013&hit=1&index=D3YWD1_MOUSE&px=1&section=5&ave_thresh=1&_ignoreionsscorebelow=20&report=0&_sigthreshold=0.05&_msresflags=1089&_msresflags2=2&percolate=-1&percolate_rt=0))  **839 - 845 381.2293 760.4440 760.4483 -6 0 R.SGFPLLK.D**  ([Ions score 28](http://10.139.25.109/mascot/cgi/peptide_view.pl?file=../data/20120413/F007793.dat&query=1176&hit=1&index=D3YWD1_MOUSE&px=1&section=5&ave_thresh=1&_ignoreionsscorebelow=20&report=0&_sigthreshold=0.05&_msresflags=1089&_msresflags2=2&percolate=-1&percolate_rt=0))  **851 - 861 593.3088 1184.6030 1184.6037 -1 0 R.VVESLDVGPDR.V**  ([Ions score 54](http://10.139.25.109/mascot/cgi/peptide_view.pl?file=../data/20120413/F007793.dat&query=17515&hit=1&index=D3YWD1_MOUSE&px=1&section=5&ave_thresh=1&_ignoreionsscorebelow=20&report=0&_sigthreshold=0.05&_msresflags=1089&_msresflags2=2&percolate=-1&percolate_rt=0))  **864 - 872 525.7823 1049.5501 1049.5506 -0 0 R.VALVQYSDR.T**  ([Ions score 40](http://10.139.25.109/mascot/cgi/peptide_view.pl?file=../data/20120413/F007793.dat&query=11213&hit=1&index=D3YWD1_MOUSE&px=1&section=5&ave_thresh=1&_ignoreionsscorebelow=20&report=0&_sigthreshold=0.05&_msresflags=1089&_msresflags2=2&percolate=-1&percolate_rt=0))  **895 - 914 1020.5791 2039.1426 2039.1415 1 0 R.LTLLGGPTPNTGAALEFVLR.N**([Ions score 85](http://10.139.25.109/mascot/cgi/peptide_view.pl?file=../data/20120413/F007793.dat&query=40848&hit=1&index=D3YWD1_MOUSE&px=1&section=5&ave_thresh=1&_ignoreionsscorebelow=20&report=0&_sigthreshold=0.05&_msresflags=1089&_msresflags2=2&percolate=-1&percolate_rt=0))  **915 - 924 518.2752 1034.5359 1034.5356 0 0 R.NILTSSTGSR.I**  ([Ions score 36](http://10.139.25.109/mascot/cgi/peptide_view.pl?file=../data/20120413/F007793.dat&query=10564&hit=1&index=D3YWD1_MOUSE&px=1&section=5&ave_thresh=1&_ignoreionsscorebelow=20&report=0&_sigthreshold=0.05&_msresflags=1089&_msresflags2=2&percolate=-1&percolate_rt=0))  **925 - 953 991.5605 2971.6581 2971.6594 0 1 R.IAEGVPQLLIVLTAEPSGDDVRGPSVVLK.Q** ([Ions score 67](http://10.139.25.109/mascot/cgi/peptide_view.pl?file=../data/20120413/F007793.dat&query=47114&hit=1&index=D3YWD1_MOUSE&px=1&section=5&ave_thresh=1&_ignoreionsscorebelow=20&report=0&_sigthreshold=0.05&_msresflags=1089&_msresflags2=2&percolate=-1&percolate_rt=0))  **990 - 1001 686.8747 1371.7348 1371.7358 -1 0 R.ELGTIQQVISER.V** ([Ions score 68](http://10.139.25.109/mascot/cgi/peptide_view.pl?file=../data/20120413/F007793.dat&query=25020&hit=1&index=D3YWD1_MOUSE&px=1&section=5&ave_thresh=1&_ignoreionsscorebelow=20&report=0&_sigthreshold=0.05&_msresflags=1089&_msresflags2=2&percolate=-1&percolate_rt=0))  **1002 - 1028 699.3981 2793.5611 2793.5600 0 1 R.VIQLNREELSSLKPILTPSTGAGVGSK.K** ([Ions score 32](http://10.139.25.109/mascot/cgi/peptide_view.pl?file=../data/20120413/F007793.dat&query=46378&hit=1&index=D3YWD1_MOUSE&px=1&section=5&ave_thresh=1&_ignoreionsscorebelow=20&report=0&_sigthreshold=0.05&_msresflags=1089&_msresflags2=2&percolate=-1&percolate_rt=0))  **1030 - 1039 560.8036 1119.5926 1119.5924 0 0 K.DVVFLIDGSR.N**  ([Ions score 22](http://10.139.25.109/mascot/cgi/peptide_view.pl?file=../data/20120413/F007793.dat&query=14371&hit=1&index=D3YWD1_MOUSE&px=1&section=5&ave_thresh=1&_ignoreionsscorebelow=20&report=0&_sigthreshold=0.05&_msresflags=1089&_msresflags2=2&percolate=-1&percolate_rt=0))  **1040 - 1049 597.8006 1193.5866 1193.5829 3 0 R.NAGPEFQYIR.T**  ([Ions score 37](http://10.139.25.109/mascot/cgi/peptide_view.pl?file=../data/20120413/F007793.dat&query=18003&hit=1&index=D3YWD1_MOUSE&px=1&section=5&ave_thresh=1&_ignoreionsscorebelow=20&report=0&_sigthreshold=0.05&_msresflags=1089&_msresflags2=2&percolate=-1&percolate_rt=0))  **1103 - 1118 855.9596 1709.9046 1709.8988 3 0 K.GGSQVYIGNALEYVLK.N**  ([Ions score 42](http://10.139.25.109/mascot/cgi/peptide_view.pl?file=../data/20120413/F007793.dat&query=33760&hit=1&index=D3YWD1_MOUSE&px=1&section=5&ave_thresh=1&_ignoreionsscorebelow=20&report=0&_sigthreshold=0.05&_msresflags=1089&_msresflags2=2&percolate=-1&percolate_rt=0))  **1129 - 1144 858.4826 1714.9506 1714.9505 0 0 R.IEEGVPQFLVLISSGK.S**  ([Ions score 75](http://10.139.25.109/mascot/cgi/peptide_view.pl?file=../data/20120413/F007793.dat&query=33884&hit=1&index=D3YWD1_MOUSE&px=1&section=5&ave_thresh=1&_ignoreionsscorebelow=20&report=0&_sigthreshold=0.05&_msresflags=1089&_msresflags2=2&percolate=-1&percolate_rt=0))  **1129 - 1144 572.6572 1714.9482 1714.9505 1 0 R.IEEGVPQFLVLISSGK.S**  ([Ions score 28](http://10.139.25.109/mascot/cgi/peptide_view.pl?file=../data/20120413/F007793.dat&query=33906&hit=1&index=D3YWD1_MOUSE&px=1&section=5&ave_thresh=1&_ignoreionsscorebelow=20&report=0&_sigthreshold=0.05&_msresflags=1089&_msresflags2=2&percolate=-1&percolate_rt=0))  **1158 - 1168 586.8434 1171.6723 1171.6713 1 0 K.QFGVAPLTIAR.H**  ([Ions score 45](http://10.139.25.109/mascot/cgi/peptide_view.pl?file=../data/20120413/F007793.dat&query=16977&hit=1&index=D3YWD1_MOUSE&px=1&section=5&ave_thresh=1&_ignoreionsscorebelow=20&report=0&_sigthreshold=0.05&_msresflags=1089&_msresflags2=2&percolate=-1&percolate_rt=0))  **1178 - 1192 874.4484 1746.8823 1746.8828 0 0 K.ISLSPEYVYSVSTFR.E**  ([Ions score 105](http://10.139.25.109/mascot/cgi/peptide_view.pl?file=../data/20120413/F007793.dat&query=34675&hit=1&index=D3YWD1_MOUSE&px=1&section=5&ave_thresh=1&_ignoreionsscorebelow=20&report=0&_sigthreshold=0.05&_msresflags=1089&_msresflags2=2&percolate=-1&percolate_rt=0))  **1201 - 1221 779.1170 2334.3276 2334.3271 0 0 K.LLTPITTLTSQQIHQILASTR.Y** ([Ions score 94](http://10.139.25.109/mascot/cgi/peptide_view.pl?file=../data/20120413/F007793.dat&query=43527&hit=1&index=D3YWD1_MOUSE&px=1&section=5&ave_thresh=1&_ignoreionsscorebelow=20&report=0&_sigthreshold=0.05&_msresflags=1089&_msresflags2=2&percolate=-1&percolate_rt=0))  **1263 - 1269 364.7192 727.4239 727.4228 1 0 R.LNIGPSK.V**  ([Ions score 30](http://10.139.25.109/mascot/cgi/peptide_view.pl?file=../data/20120413/F007793.dat&query=441&hit=1&index=D3YWD1_MOUSE&px=1&section=5&ave_thresh=1&_ignoreionsscorebelow=20&report=0&_sigthreshold=0.05&_msresflags=1089&_msresflags2=2&percolate=-1&percolate_rt=0))  **1272 - 1288 1001.5193 2001.0240 2001.0248 0 0 R.IGVVQFSNDVFPEFYLK.T**  ([Ions score 58](http://10.139.25.109/mascot/cgi/peptide_view.pl?file=../data/20120413/F007793.dat&query=40292&hit=1&index=D3YWD1_MOUSE&px=1&section=5&ave_thresh=1&_ignoreionsscorebelow=20&report=0&_sigthreshold=0.05&_msresflags=1089&_msresflags2=2&percolate=-1&percolate_rt=0))  **1272 - 1288 668.0167 2001.0267 2001.0248 1 0 R.IGVVQFSNDVFPEFYLK.T**  ([Ions score 35](http://10.139.25.109/mascot/cgi/peptide_view.pl?file=../data/20120413/F007793.dat&query=40315&hit=1&index=D3YWD1_MOUSE&px=1&section=5&ave_thresh=1&_ignoreionsscorebelow=20&report=0&_sigthreshold=0.05&_msresflags=1089&_msresflags2=2&percolate=-1&percolate_rt=0))  **1292 - 1301 545.2983 1088.5820 1088.5826 -1 0 K.SQSSVLEAIR.R**  ([Ions score 67](http://10.139.25.109/mascot/cgi/peptide_view.pl?file=../data/20120413/F007793.dat&query=12898&hit=1&index=D3YWD1_MOUSE&px=1&section=5&ave_thresh=1&_ignoreionsscorebelow=20&report=0&_sigthreshold=0.05&_msresflags=1089&_msresflags2=2&percolate=-1&percolate_rt=0))  **1316 - 1322 403.2321 804.4496 804.4494 0 0 R.ALEFVAR.N**  ([Ions score 42](http://10.139.25.109/mascot/cgi/peptide_view.pl?file=../data/20120413/F007793.dat&query=2225&hit=1&index=D3YWD1_MOUSE&px=1&section=5&ave_thresh=1&_ignoreionsscorebelow=20&report=0&_sigthreshold=0.05&_msresflags=1089&_msresflags2=2&percolate=-1&percolate_rt=0))  **1333 - 1348 574.6572 1720.9499 1720.9512 -1 0 R.IEDGVPQHLVLFLGGK.S**  ([Ions score 45](http://10.139.25.109/mascot/cgi/peptide_view.pl?file=../data/20120413/F007793.dat&query=34017&hit=1&index=D3YWD1_MOUSE&px=1&section=5&ave_thresh=1&_ignoreionsscorebelow=20&report=0&_sigthreshold=0.05&_msresflags=1089&_msresflags2=2&percolate=-1&percolate_rt=0))  **1333 - 1348 861.4834 1720.9512 1720.9512 0 0 R.IEDGVPQHLVLFLGGK.S**  ([Ions score 34](http://10.139.25.109/mascot/cgi/peptide_view.pl?file=../data/20120413/F007793.dat&query=34025&hit=1&index=D3YWD1_MOUSE&px=1&section=5&ave_thresh=1&_ignoreionsscorebelow=20&report=0&_sigthreshold=0.05&_msresflags=1089&_msresflags2=2&percolate=-1&percolate_rt=0))  **1374 - 1388 886.4488 1770.8820 1770.8860 2 1 R.NIDRTDLQTITNDPR.L**  ([Ions score 33](http://10.139.25.109/mascot/cgi/peptide_view.pl?file=../data/20120413/F007793.dat&query=35319&hit=1&index=D3YWD1_MOUSE&px=1&section=5&ave_thresh=1&_ignoreionsscorebelow=20&report=0&_sigthreshold=0.05&_msresflags=1089&_msresflags2=2&percolate=-1&percolate_rt=0))  **1389 - 1394 367.7310 733.4475 733.4487 -2 0 R.LVFTVR.E**  ([Ions score 36](http://10.139.25.109/mascot/cgi/peptide_view.pl?file=../data/20120413/F007793.dat&query=609&hit=1&index=D3YWD1_MOUSE&px=1&section=5&ave_thresh=1&_ignoreionsscorebelow=20&report=0&_sigthreshold=0.05&_msresflags=1089&_msresflags2=2&percolate=-1&percolate_rt=0))  **1406 - 1432 916.8063 2747.3955 2747.3953 0 0 R.VMLSFGPSGATPQPPGVDLPSPSRPEK.K** ([Ions score 65](http://10.139.25.109/mascot/cgi/peptide_view.pl?file=../data/20120413/F007793.dat&query=46163&hit=1&index=D3YWD1_MOUSE&px=1&section=5&ave_thresh=1&_ignoreionsscorebelow=20&report=0&_sigthreshold=0.05&_msresflags=1089&_msresflags2=2&percolate=-1&percolate_rt=0))  **1450 - 1457 497.2539 992.4932 992.4927 1 0 R.DSFQEVLR.F**  ([Ions score 51](http://10.139.25.109/mascot/cgi/peptide_view.pl?file=../data/20120413/F007793.dat&query=8743&hit=1&index=D3YWD1_MOUSE&px=1&section=5&ave_thresh=1&_ignoreionsscorebelow=20&report=0&_sigthreshold=0.05&_msresflags=1089&_msresflags2=2&percolate=-1&percolate_rt=0))  **1458 - 1474 958.4502 1914.8858 1914.8847 1 0 R.FASEIVDTVYEDGDSIR.V**  ([Ions score 64](http://10.139.25.109/mascot/cgi/peptide_view.pl?file=../data/20120413/F007793.dat&query=38695&hit=1&index=D3YWD1_MOUSE&px=1&section=5&ave_thresh=1&_ignoreionsscorebelow=20&report=0&_sigthreshold=0.05&_msresflags=1089&_msresflags2=2&percolate=-1&percolate_rt=0))  **1475 - 1491 1000.4897 1998.9648 1998.9687 -2 0 R.VGLVQYNSDPTDEFFLR.D**  ([Ions score 78](http://10.139.25.109/mascot/cgi/peptide_view.pl?file=../data/20120413/F007793.dat&query=40255&hit=1&index=D3YWD1_MOUSE&px=1&section=5&ave_thresh=1&_ignoreionsscorebelow=20&report=0&_sigthreshold=0.05&_msresflags=1089&_msresflags2=2&percolate=-1&percolate_rt=0))  4. [PTGIS_MOUSE](http://10.139.25.109/mascot/cgi/protein_view.pl?file=../data/20120413/F007793.dat&hit=PTGIS_MOUSE&db_idx=1&px=1&ave_thresh=1&_ignoreionsscorebelow=20&report=0&_sigthreshold=0.05&_msresflags=1089&_msresflags2=2&percolate=-1&percolate_rt=0)    **Mass:** 57125    **Score:** 1278   **Matches:** 26(26)  **Sequences:** 19(19)  Prostacyclin synthase  Sequence Coverage: **47%**; Matched peptides shown in **Bold Red**  **1** MSWAALLGLL AVLLLLLLLL SRRRARRPGE PPLDLGSIPW LGHALEFGR**D AASFLTR**MKE K**HGDIFTVLV GGRYVTVLLD PHSYDTVVWE LR**TR**LDFHPY**  **101 AIFLMERIFD LQLPNFNPSE EK**ARMKPTLM HR**DLQALTEA MYTNLR**TVLL GDSTEAGSGW QETGLLEFSY NALLSAGYLT LYGVEASPRT HESQAQDR**VH**  **201 SADVFHTFRQ LDLLLPK**LAR GSLSAGDKDH ACSVKNRLWK LLSPARLASR ADR**SSWLESY LR**HLEEMGVS EEMQARALVL QLWATQGNMG PTAFWLLLFL  **301** LKNPEALAAV RAELK**HTVWQ AEQPVSQMTT LPQKILDSMP VLDSVLNETL RLTAAPFITR EVMADLALPM ADGR**EFSLRR GDR**LLLFPFL SPQKDPEIYT**  **401 EPEVFK**YNR**F LNPDGSEK**KD FYKDGKRLK**N YNMPWGAGHN QCLGKSYAIN SIK**QFVVLLL THFDLELGSE DTEVPEFDLS R**YGFGLMQPE EDVPIR**YRAR  **501** L  **Start - End Observed Mr(expt) Mr(calc) ppm Miss Sequence**  **50 - 57 440.7299 879.4452 879.4450 0 0 R.DAASFLTR.M**  ([Ions score 41](http://10.139.25.109/mascot/cgi/peptide_view.pl?file=../data/20120413/F007793.dat&query=4388&hit=1&index=PTGIS_MOUSE&px=1&section=5&ave_thresh=1&_ignoreionsscorebelow=20&report=0&_sigthreshold=0.05&_msresflags=1089&_msresflags2=2&percolate=-1&percolate_rt=0))  **62 - 73 635.8479 1269.6812 1269.6830 -1 0 K.HGDIFTVLVGGR.Y**  ([Ions score 79](http://10.139.25.109/mascot/cgi/peptide_view.pl?file=../data/20120413/F007793.dat&query=21757&hit=1&index=PTGIS_MOUSE&px=1&section=5&ave_thresh=1&_ignoreionsscorebelow=20&report=0&_sigthreshold=0.05&_msresflags=1089&_msresflags2=2&percolate=-1&percolate_rt=0))  **74 - 92 769.0651 2304.1735 2304.1790 -2 0 R.YVTVLLDPHSYDTVVWELR.T**  ([Ions score 46](http://10.139.25.109/mascot/cgi/peptide_view.pl?file=../data/20120413/F007793.dat&query=43340&hit=1&index=PTGIS_MOUSE&px=1&section=5&ave_thresh=1&_ignoreionsscorebelow=20&report=0&_sigthreshold=0.05&_msresflags=1089&_msresflags2=2&percolate=-1&percolate_rt=0))  **95 - 107 551.2822 1650.8247 1650.8228 1 0 R.LDFHPYAIFLMER.I**  ([Ions score 49](http://10.139.25.109/mascot/cgi/peptide_view.pl?file=../data/20120413/F007793.dat&query=32270&hit=1&index=PTGIS_MOUSE&px=1&section=5&ave_thresh=1&_ignoreionsscorebelow=20&report=0&_sigthreshold=0.05&_msresflags=1089&_msresflags2=2&percolate=-1&percolate_rt=0))  **95 - 107 826.4197 1650.8248 1650.8228 1 0 R.LDFHPYAIFLMER.I**  ([Ions score 52](http://10.139.25.109/mascot/cgi/peptide_view.pl?file=../data/20120413/F007793.dat&query=32271&hit=1&index=PTGIS_MOUSE&px=1&section=5&ave_thresh=1&_ignoreionsscorebelow=20&report=0&_sigthreshold=0.05&_msresflags=1089&_msresflags2=2&percolate=-1&percolate_rt=0))  **108 - 122 895.9547 1789.8949 1789.8886 3 0 R.IFDLQLPNFNPSEEK.A**  ([Ions score 61](http://10.139.25.109/mascot/cgi/peptide_view.pl?file=../data/20120413/F007793.dat&query=35825&hit=1&index=PTGIS_MOUSE&px=1&section=5&ave_thresh=1&_ignoreionsscorebelow=20&report=0&_sigthreshold=0.05&_msresflags=1089&_msresflags2=2&percolate=-1&percolate_rt=0))  **108 - 122 597.6371 1790.8877 1789.8887 -1 0 R.IFDLQLPNFNPSEEK.A**  ([Ions score 42](http://10.139.25.109/mascot/cgi/peptide_view.pl?file=../data/20120413/F007793.dat&query=35855&hit=1&index=PTGIS_MOUSE&px=1&section=5&ave_thresh=1&_ignoreionsscorebelow=20&report=0&_sigthreshold=0.05&_msresflags=1089&_msresflags2=2&percolate=-1&percolate_rt=0))  **133 - 146 819.9116 1637.8086 1637.8083 0 0 R.DLQALTEAMYTNLR.T**  ([Ions score 29](http://10.139.25.109/mascot/cgi/peptide_view.pl?file=../data/20120413/F007793.dat&query=31875&hit=1&index=PTGIS_MOUSE&px=1&section=5&ave_thresh=1&_ignoreionsscorebelow=20&report=0&_sigthreshold=0.05&_msresflags=1089&_msresflags2=2&percolate=-1&percolate_rt=0))  **199 - 209 329.6688 1314.6459 1314.6469 -1 0 R.VHSADVFHTFR.Q**  ([Ions score 24](http://10.139.25.109/mascot/cgi/peptide_view.pl?file=../data/20120413/F007793.dat&query=23545&hit=1&index=PTGIS_MOUSE&px=1&section=5&ave_thresh=1&_ignoreionsscorebelow=20&report=0&_sigthreshold=0.05&_msresflags=1089&_msresflags2=2&percolate=-1&percolate_rt=0))  **199 - 209 439.2228 1314.6467 1314.6469 0 0 R.VHSADVFHTFR.Q**  ([Ions score 43](http://10.139.25.109/mascot/cgi/peptide_view.pl?file=../data/20120413/F007793.dat&query=23546&hit=1&index=PTGIS_MOUSE&px=1&section=5&ave_thresh=1&_ignoreionsscorebelow=20&report=0&_sigthreshold=0.05&_msresflags=1089&_msresflags2=2&percolate=-1&percolate_rt=0))  **199 - 209 658.3312 1314.6479 1314.6469 1 0 R.VHSADVFHTFR.Q**  ([Ions score 71](http://10.139.25.109/mascot/cgi/peptide_view.pl?file=../data/20120413/F007793.dat&query=23547&hit=1&index=PTGIS_MOUSE&px=1&section=5&ave_thresh=1&_ignoreionsscorebelow=20&report=0&_sigthreshold=0.05&_msresflags=1089&_msresflags2=2&percolate=-1&percolate_rt=0))  **210 - 217 470.2973 938.5800 938.5800 0 0 R.QLDLLLPK.L**  ([Ions score 34](http://10.139.25.109/mascot/cgi/peptide_view.pl?file=../data/20120413/F007793.dat&query=6612&hit=1&index=PTGIS_MOUSE&px=1&section=5&ave_thresh=1&_ignoreionsscorebelow=20&report=0&_sigthreshold=0.05&_msresflags=1089&_msresflags2=2&percolate=-1&percolate_rt=0))  **254 - 262 570.7883 1139.5621 1139.5611 1 0 R.SSWLESYLR.H**  ([Ions score 63](http://10.139.25.109/mascot/cgi/peptide_view.pl?file=../data/20120413/F007793.dat&query=15390&hit=1&index=PTGIS_MOUSE&px=1&section=5&ave_thresh=1&_ignoreionsscorebelow=20&report=0&_sigthreshold=0.05&_msresflags=1089&_msresflags2=2&percolate=-1&percolate_rt=0))  **316 - 334 737.0411 2208.0999 2208.0997 0 0 K.HTVWQAEQPVSQMTTLPQK.I** ([Ions score 91](http://10.139.25.109/mascot/cgi/peptide_view.pl?file=../data/20120413/F007793.dat&query=42614&hit=1&index=PTGIS_MOUSE&px=1&section=5&ave_thresh=1&_ignoreionsscorebelow=20&report=0&_sigthreshold=0.05&_msresflags=1089&_msresflags2=2&percolate=-1&percolate_rt=0))  **335 - 351 958.0166 1914.0186 1914.0132 3 0 K.ILDSMPVLDSVLNETLR.L**  ([Ions score 53](http://10.139.25.109/mascot/cgi/peptide_view.pl?file=../data/20120413/F007793.dat&query=38691&hit=1&index=PTGIS_MOUSE&px=1&section=5&ave_thresh=1&_ignoreionsscorebelow=20&report=0&_sigthreshold=0.05&_msresflags=1089&_msresflags2=2&percolate=-1&percolate_rt=0))  **335 - 351 639.0116 1914.0114 1914.0132 1 0 K.ILDSMPVLDSVLNETLR.L**  ([Ions score 65](http://10.139.25.109/mascot/cgi/peptide_view.pl?file=../data/20120413/F007793.dat&query=38705&hit=1&index=PTGIS_MOUSE&px=1&section=5&ave_thresh=1&_ignoreionsscorebelow=20&report=0&_sigthreshold=0.05&_msresflags=1089&_msresflags2=2&percolate=-1&percolate_rt=0))  **335 - 351 644.3422 1930.0048 1930.0081 -2 0 K.ILDSMPVLDSVLNETLR.L**  Oxidation (M) ([Ions score 41](http://10.139.25.109/mascot/cgi/peptide_view.pl?file=../data/20120413/F007793.dat&query=38978&hit=1&index=PTGIS_MOUSE&px=1&section=5&ave_thresh=1&_ignoreionsscorebelow=20&report=0&_sigthreshold=0.05&_msresflags=1089&_msresflags2=2&percolate=-1&percolate_rt=0))  **352 - 360 495.2921 988.5695 988.5706 -1 0 R.LTAAPFITR.E**  ([Ions score 32](http://10.139.25.109/mascot/cgi/peptide_view.pl?file=../data/20120413/F007793.dat&query=8609&hit=1&index=PTGIS_MOUSE&px=1&section=5&ave_thresh=1&_ignoreionsscorebelow=20&report=0&_sigthreshold=0.05&_msresflags=1089&_msresflags2=2&percolate=-1&percolate_rt=0))  **361 - 374 744.8602 1487.7058 1487.7112 -4 0 R.EVMADLALPMADGR.E**  ([Ions score 38](http://10.139.25.109/mascot/cgi/peptide_view.pl?file=../data/20120413/F007793.dat&query=27688&hit=1&index=PTGIS_MOUSE&px=1&section=5&ave_thresh=1&_ignoreionsscorebelow=20&report=0&_sigthreshold=0.05&_msresflags=1089&_msresflags2=2&percolate=-1&percolate_rt=0))  **384 - 394 651.8955 1301.7765 1301.7747 1 0 R.LLLFPFLSPQK.D**  ([Ions score 39](http://10.139.25.109/mascot/cgi/peptide_view.pl?file=../data/20120413/F007793.dat&query=22988&hit=1&index=PTGIS_MOUSE&px=1&section=5&ave_thresh=1&_ignoreionsscorebelow=20&report=0&_sigthreshold=0.05&_msresflags=1089&_msresflags2=2&percolate=-1&percolate_rt=0))  **384 - 406 917.4950 2749.4616 2749.4619 0 1 R.LLLFPFLSPQKDPEIYTEPEVFK.Y** ([Ions score 72](http://10.139.25.109/mascot/cgi/peptide_view.pl?file=../data/20120413/F007793.dat&query=46177&hit=1&index=PTGIS_MOUSE&px=1&section=5&ave_thresh=1&_ignoreionsscorebelow=20&report=0&_sigthreshold=0.05&_msresflags=1089&_msresflags2=2&percolate=-1&percolate_rt=0))  **410 - 418 503.7449 1005.4753 1005.4767 -1 0 R.FLNPDGSEK.K**  ([Ions score 25](http://10.139.25.109/mascot/cgi/peptide_view.pl?file=../data/20120413/F007793.dat&query=9310&hit=1&index=PTGIS_MOUSE&px=1&section=5&ave_thresh=1&_ignoreionsscorebelow=20&report=0&_sigthreshold=0.05&_msresflags=1089&_msresflags2=2&percolate=-1&percolate_rt=0))  **430 - 445 616.2760 1845.8046 1845.8039 0 0 K.NYNMPWGAGHNQCLGK.S** ([Ions score 21](http://10.139.25.109/mascot/cgi/peptide_view.pl?file=../data/20120413/F007793.dat&query=37360&hit=1&index=PTGIS_MOUSE&px=1&section=5&ave_thresh=1&_ignoreionsscorebelow=20&report=0&_sigthreshold=0.05&_msresflags=1089&_msresflags2=2&percolate=-1&percolate_rt=0))  **446 - 453 448.2473 894.4801 894.4810 -1 0 K.SYAINSIK.Q**  ([Ions score 21](http://10.139.25.109/mascot/cgi/peptide_view.pl?file=../data/20120413/F007793.dat&query=4928&hit=1&index=PTGIS_MOUSE&px=1&section=5&ave_thresh=1&_ignoreionsscorebelow=20&report=0&_sigthreshold=0.05&_msresflags=1089&_msresflags2=2&percolate=-1&percolate_rt=0))  **482 - 496 875.9272 1749.8399 1749.8396 0 0 R.YGFGLMQPEEDVPIR.Y**  ([Ions score 79](http://10.139.25.109/mascot/cgi/peptide_view.pl?file=../data/20120413/F007793.dat&query=34736&hit=1&index=PTGIS_MOUSE&px=1&section=5&ave_thresh=1&_ignoreionsscorebelow=20&report=0&_sigthreshold=0.05&_msresflags=1089&_msresflags2=2&percolate=-1&percolate_rt=0))  **482 - 496 883.9224 1765.8302 1765.8345 -2 0 R.YGFGLMQPEEDVPIR.Y**  Oxidation (M) ([Ions score 67](http://10.139.25.109/mascot/cgi/peptide_view.pl?file=../data/20120413/F007793.dat&query=35156&hit=1&index=PTGIS_MOUSE&px=1&section=5&ave_thresh=1&_ignoreionsscorebelow=20&report=0&_sigthreshold=0.05&_msresflags=1089&_msresflags2=2&percolate=-1&percolate_rt=0))    5. [SDPR_MOUSE](http://10.139.25.109/mascot/cgi/protein_view.pl?file=../data/20120413/F007791.dat&hit=SDPR_MOUSE&db_idx=1&px=1&ave_thresh=1&_ignoreionsscorebelow=20&report=0&_sigthreshold=0.05&_msresflags=1089&_msresflags2=2&percolate=-1&percolate_rt=0)    **Mass:** 46792    **Score:** 394    **Matches:** 8(8)  **Sequences:** 7(7)  Serum deprivation-response protein  Sequence Coverage: **22%**; Matched peptides shown in **Bold Red**  **1** MGEDAAQAEK FQHPNTDMLQ EKPSSPSPMP SSTPSPSLNL GSTEEAIRDN SQVNAVTVHT LLDK**LVNMLD AVR**ENQHNME QRQINLEGSV KGIQNDLTKL  **101** SK**YQASTSNT VSK**LLEKSRK VSAHTRAVRE RLERQCVQVK RLENNHAQLL RRNHFK**VLIF QEESEIPASV FVKEPVPSAA EGKEELADEN K**SLEETLHNV  **201** DLSSDDELPR DEEALEDSAE EKMEESRAEK IKRSSLKKVD SLKKAFSR**QN IEKKMNK**LGT KIVSVERREK IKKSLTPNHQ KASSGKSSPF KVSPLSFGRK  **301** KVREGESSVE NETKLEDQMQ EDREEGSFTE GLSEASLPSG LMEGSAEDAE KSARRGNNSA VGSNADLTIE EDEEEEPVAL QQAQQVR**YES GYMLNSEEME**  **401 EPSEKQVQPA VLHVDQTA**  **Start - End Observed Mr(expt) Mr(calc) ppm Miss Sequence**  **65 - 73 515.7897 1029.5648 1029.5641 1 0 K.LVNMLDAVR.E**  ([Ions score 67](http://10.139.25.109/mascot/cgi/peptide_view.pl?file=../data/20120413/F007791.dat&query=5752&hit=1&index=SDPR_MOUSE&px=1&section=5&ave_thresh=1&_ignoreionsscorebelow=20&report=0&_sigthreshold=0.05&_msresflags=1089&_msresflags2=2&percolate=-1&percolate_rt=0))  **103 - 113 593.7824 1185.5503 1185.5513 -1 0 K.YQASTSNTVSK.L**  ([Ions score 20](http://10.139.25.109/mascot/cgi/peptide_view.pl?file=../data/20120413/F007791.dat&query=11335&hit=1&index=SDPR_MOUSE&px=1&section=5&ave_thresh=1&_ignoreionsscorebelow=20&report=0&_sigthreshold=0.05&_msresflags=1089&_msresflags2=2&percolate=-1&percolate_rt=0))  **157 - 173 968.0293 1934.0440 1934.0401 2 0 K.VLIFQEESEIPASVFVK.E**  ([Ions score 29](http://10.139.25.109/mascot/cgi/peptide_view.pl?file=../data/20120413/F007791.dat&query=37949&hit=1&index=SDPR_MOUSE&px=1&section=5&ave_thresh=1&_ignoreionsscorebelow=20&report=0&_sigthreshold=0.05&_msresflags=1089&_msresflags2=2&percolate=-1&percolate_rt=0))  **157 - 173 968.0276 1934.0396 1934.0401 0 0 K.VLIFQEESEIPASVFVK.E**  ([Ions score 39](http://10.139.25.109/mascot/cgi/peptide_view.pl?file=../data/20120413/F007791.dat&query=38006&hit=1&index=SDPR_MOUSE&px=1&section=5&ave_thresh=1&_ignoreionsscorebelow=20&report=0&_sigthreshold=0.05&_msresflags=1089&_msresflags2=2&percolate=-1&percolate_rt=0))  **174 - 191 638.3096 1911.9069 1911.9061 0 1 K.EPVPSAAEGKEELADENK.S**  ([Ions score 58](http://10.139.25.109/mascot/cgi/peptide_view.pl?file=../data/20120413/F007791.dat&query=37263&hit=1&index=SDPR_MOUSE&px=1&section=5&ave_thresh=1&_ignoreionsscorebelow=20&report=0&_sigthreshold=0.05&_msresflags=1089&_msresflags2=2&percolate=-1&percolate_rt=0))  **249 - 257 566.8115 1131.6074 1131.6070 0 2 R.QNIEKKMNK.L** ([Ions score 25](http://10.139.25.109/mascot/cgi/peptide_view.pl?file=../data/20120413/F007791.dat&query=9441&hit=1&index=SDPR_MOUSE&px=1&section=5&ave_thresh=1&_ignoreionsscorebelow=20&report=0&_sigthreshold=0.05&_msresflags=1089&_msresflags2=2&percolate=-1&percolate_rt=0))  **388 - 405 1076.4358 2150.8570 2150.8660 -4 0 R.YESGYMLNSEEMEEPSEK.Q**  ([Ions score 92](http://10.139.25.109/mascot/cgi/peptide_view.pl?file=../data/20120413/F007791.dat&query=43512&hit=1&index=SDPR_MOUSE&px=1&section=5&ave_thresh=1&_ignoreionsscorebelow=20&report=0&_sigthreshold=0.05&_msresflags=1089&_msresflags2=2&percolate=-1&percolate_rt=0))  **406 - 418 703.3746 1404.7346 1404.7361 -1 0 K.QVQPAVLHVDQTA.-**([Ions score 64](http://10.139.25.109/mascot/cgi/peptide_view.pl?file=../data/20120413/F007791.dat&query=19826&hit=1&index=SDPR_MOUSE&px=1&section=5&ave_thresh=1&_ignoreionsscorebelow=20&report=0&_sigthreshold=0.05&_msresflags=1089&_msresflags2=2&percolate=-1&percolate_rt=0))  6. [Q3UBU9_MOUSE](http://10.139.25.109/mascot/cgi/protein_view.pl?file=../data/20120413/F007791.dat&hit=Q3UBU9_MOUSE&db_idx=1&px=1&ave_thresh=1&_ignoreionsscorebelow=20&report=0&_sigthreshold=0.05&_msresflags=1089&_msresflags2=2&percolate=-1&percolate_rt=0)    **Mass:** 25189    **Score:** 430   **Matches:** 9(9)  **Sequences:** 7(7)  FK506 Binding Protein 3  Sequence Coverage: **33%**; Matched peptides shown in **Bold Red**  **1** MAAAVPQR**AW TVEQLR**SEQL PKKDIIK**FLQ DHGSDSFLAE HK**LLGNIKNV AK**TANKDHLV NAYNHLFESK** RFKGTETISK VSEQVKNVKL SDDKPKDSKS  **101** EETLDEGPPK YTKSILKKGD KTNFPKKGDV VHCWYTGTLP DGTVFDTNIQ TSSKKKKNAK PLSFKVGVGK VIR**GWDEALL TMSKGEK**AR**L EIEPEWAYGK**  **201** KGQPDAKIPP NTKLIFEVEL VDID  **Start - End Observed Mr(expt) Mr(calc) ppm Miss Sequence**  **9 - 16 501.7719 1001.5292 1001.5294 0 0 R.AWTVEQLR.S**  ([Ions score 44](http://10.139.25.109/mascot/cgi/peptide_view.pl?file=../data/20120413/F007791.dat&query=4949&hit=1&index=Q3UBU9_MOUSE&px=1&section=5&ave_thresh=1&_ignoreionsscorebelow=20&report=0&_sigthreshold=0.05&_msresflags=1089&_msresflags2=2&percolate=-1&percolate_rt=0))  **28 - 42 577.6091 1729.8056 1729.8060 0 0 K.FLQDHGSDSFLAEHK.L**  ([Ions score 59](http://10.139.25.109/mascot/cgi/peptide_view.pl?file=../data/20120413/F007791.dat&query=30795&hit=1&index=Q3UBU9_MOUSE&px=1&section=5&ave_thresh=1&_ignoreionsscorebelow=20&report=0&_sigthreshold=0.05&_msresflags=1089&_msresflags2=2&percolate=-1&percolate_rt=0))  **28 - 42 433.4589 1729.8063 1729.8060 0 0 K.FLQDHGSDSFLAEHK.L**  ([Ions score 44](http://10.139.25.109/mascot/cgi/peptide_view.pl?file=../data/20120413/F007791.dat&query=30798&hit=1&index=Q3UBU9_MOUSE&px=1&section=5&ave_thresh=1&_ignoreionsscorebelow=20&report=0&_sigthreshold=0.05&_msresflags=1089&_msresflags2=2&percolate=-1&percolate_rt=0))  **28 - 42 577.6095 1729.8067 1729.8060 0 0 K.FLQDHGSDSFLAEHK.L**  ([Ions score 50](http://10.139.25.109/mascot/cgi/peptide_view.pl?file=../data/20120413/F007791.dat&query=30799&hit=1&index=Q3UBU9_MOUSE&px=1&section=5&ave_thresh=1&_ignoreionsscorebelow=20&report=0&_sigthreshold=0.05&_msresflags=1089&_msresflags2=2&percolate=-1&percolate_rt=0))  **53 - 70 526.0175 2100.0407 2100.0388 1 1 K.TANKDHLVNAYNHLFESK.R**  ([Ions score 34](http://10.139.25.109/mascot/cgi/peptide_view.pl?file=../data/20120413/F007791.dat&query=42576&hit=1&index=Q3UBU9_MOUSE&px=1&section=5&ave_thresh=1&_ignoreionsscorebelow=20&report=0&_sigthreshold=0.05&_msresflags=1089&_msresflags2=2&percolate=-1&percolate_rt=0))  **158 - 165 452.7666 903.5187 903.5178 1 0 K.NAKPLSFK.V**  ([Ions score 23](http://10.139.25.109/mascot/cgi/peptide_view.pl?file=../data/20120413/F007793.dat&query=5237&hit=1&index=Q3UBU9_MOUSE&px=1&section=5&ave_thresh=1&_ignoreionsscorebelow=20&report=0&_sigthreshold=0.05&_msresflags=1089&_msresflags2=2&percolate=-1&percolate_rt=0))  **174 - 184 625.8075 1249.6004 1249.6013 -1 0 R.GWDEALLTMSK.G**  ([Ions score 76](http://10.139.25.109/mascot/cgi/peptide_view.pl?file=../data/20120413/F007791.dat&query=14395&hit=1&index=Q3UBU9_MOUSE&px=1&section=5&ave_thresh=1&_ignoreionsscorebelow=20&report=0&_sigthreshold=0.05&_msresflags=1089&_msresflags2=2&percolate=-1&percolate_rt=0))  **174 - 187 782.8860 1563.7575 1563.7603 -2 1 R.GWDEALLTMSKGEK.A**  ([Ions score 54](http://10.139.25.109/mascot/cgi/peptide_view.pl?file=../data/20120413/F007791.dat&query=24827&hit=1&index=Q3UBU9_MOUSE&px=1&section=5&ave_thresh=1&_ignoreionsscorebelow=20&report=0&_sigthreshold=0.05&_msresflags=1089&_msresflags2=2&percolate=-1&percolate_rt=0))  **190 - 200 667.8347 1333.6549 1333.6554 -0 0 R.LEIEPEWAYGK.K**  ([Ions score 46](http://10.139.25.109/mascot/cgi/peptide_view.pl?file=../data/20120413/F007791.dat&query=17877&hit=1&index=Q3UBU9_MOUSE&px=1&section=5&ave_thresh=1&_ignoreionsscorebelow=20&report=0&_sigthreshold=0.05&_msresflags=1089&_msresflags2=2&percolate=-1&percolate_rt=0))  7. [ADK_MOUSE](http://10.139.25.109/mascot/cgi/protein_view.pl?file=../data/20120413/F007792.dat&hit=ADK_MOUSE&db_idx=1&px=1&ave_thresh=1&_ignoreionsscorebelow=20&report=0&_sigthreshold=0.05&_msresflags=1089&_msresflags2=2&percolate=-1&percolate_rt=0)    **Mass:** 40466    **Score:** 453    **Matches:** 10(10)  **Sequences:** 9(9)  Adenosine kinase  Sequence Coverage: **36%;** Matched peptides shown in **Bold Red**  **1** MAAADEPKPK KLK**VEAPQAL SENVLFGMGN PLLDISAVVD KDFLDK**YSLK PNDQILAEDK HKELFDELVK KFKVEYHAGG STQNSMK**VAQ WLIQEPHKAA**  **101 TFFGCIGIDK FGEILK**RK**AA DAHVDAHYYE QNEQPTGTCA ACITGGNRSL VANLAAANCY K**KEKHLDLER **NWVLVEK**ARV YYIAGFFLTV SPESVLKVAR  **201** YAAENNRVFT LNLSAPFISQ FFKEALMDVM PYVDILFGNE TEAATFAREQ GFETKDIKEI AKKAQALPKV NSKRQR**TVIF TQGR**DDTIVA AENDVTAFPV  **301** LDQNQEEIID TNGAGDAFVG GFLSQLVSDK PLTECIR**AGH YAASVIIR**RT GCTFPEKPDF H  **Start - End Observed Mr(expt) Mr(calc) ppm Miss Sequence**  **14 - 46 1182.2800 3543.8166 3543.8171 0 1 K.VEAPQALSENVLFGMGNPLLDISAVVDKDFLDK.Y** ([Ions score 95](http://10.139.25.109/mascot/cgi/peptide_view.pl?file=../data/20120413/F007792.dat&query=45230&hit=1&index=ADK_MOUSE&px=1&section=5&ave_thresh=1&_ignoreionsscorebelow=20&report=0&_sigthreshold=0.05&_msresflags=1089&_msresflags2=2&percolate=-1&percolate_rt=0))  **88 - 98 450.2505 1347.7296 1347.7299 0 0 K.VAQWLIQEPHK.A**  ([Ions score 34](http://10.139.25.109/mascot/cgi/peptide_view.pl?file=../data/20120413/F007792.dat&query=14482&hit=1&index=ADK_MOUSE&px=1&section=5&ave_thresh=1&_ignoreionsscorebelow=20&report=0&_sigthreshold=0.05&_msresflags=1089&_msresflags2=2&percolate=-1&percolate_rt=0))  **99 - 110 650.3237 1298.6329 1298.6329 0 0 K.AATFFGCIGIDK.F**  ([Ions score 65](http://10.139.25.109/mascot/cgi/peptide_view.pl?file=../data/20120413/F007792.dat&query=13186&hit=1&index=ADK_MOUSE&px=1&section=5&ave_thresh=1&_ignoreionsscorebelow=20&report=0&_sigthreshold=0.05&_msresflags=1089&_msresflags2=2&percolate=-1&percolate_rt=0))  **99 - 116 663.0163 1986.0271 1986.0285 -1 1 K.AATFFGCIGIDKFGEILK.R** ([Ions score 21](http://10.139.25.109/mascot/cgi/peptide_view.pl?file=../data/20120413/F007792.dat&query=31005&hit=1&index=ADK_MOUSE&px=1&section=5&ave_thresh=1&_ignoreionsscorebelow=20&report=0&_sigthreshold=0.05&_msresflags=1089&_msresflags2=2&percolate=-1&percolate_rt=0))  **119 - 148 820.1070 3276.3967 3276.3949 1 0 K.AADAHVDAHYYEQNEQPTGTCAACITGGNR.S** ([Ions score 38](http://10.139.25.109/mascot/cgi/peptide_view.pl?file=../data/20120413/F007792.dat&query=44002&hit=1&index=ADK_MOUSE&px=1&section=5&ave_thresh=1&_ignoreionsscorebelow=20&report=0&_sigthreshold=0.05&_msresflags=1089&_msresflags2=2&percolate=-1&percolate_rt=0))  **149 - 161 697.8590 1393.7033 1393.7023 1 0 R.SLVANLAAANCYK.K**  ([Ions score 99](http://10.139.25.109/mascot/cgi/peptide_view.pl?file=../data/20120413/F007792.dat&query=15380&hit=1&index=ADK_MOUSE&px=1&section=5&ave_thresh=1&_ignoreionsscorebelow=20&report=0&_sigthreshold=0.05&_msresflags=1089&_msresflags2=2&percolate=-1&percolate_rt=0))  **171 - 177 444.2513 886.4881 886.4912 -4 0 R.NWVLVEK.A**  ([Ions score 20](http://10.139.25.109/mascot/cgi/peptide_view.pl?file=../data/20120413/F007792.dat&query=1732&hit=1&index=ADK_MOUSE&px=1&section=5&ave_thresh=1&_ignoreionsscorebelow=20&report=0&_sigthreshold=0.05&_msresflags=1089&_msresflags2=2&percolate=-1&percolate_rt=0))  **277 - 284 461.2607 920.5069 920.5080 -1 0 R.TVIFTQGR.D**  ([Ions score 20](http://10.139.25.109/mascot/cgi/peptide_view.pl?file=../data/20120413/F007792.dat&query=2344&hit=1&index=ADK_MOUSE&px=1&section=5&ave_thresh=1&_ignoreionsscorebelow=20&report=0&_sigthreshold=0.05&_msresflags=1089&_msresflags2=2&percolate=-1&percolate_rt=0))  **338 - 348 386.5520 1156.6343 1156.6353 -1 0 R.AGHYAASVIIR.R**  ([Ions score 36](http://10.139.25.109/mascot/cgi/peptide_view.pl?file=../data/20120413/F007792.dat&query=8422&hit=1&index=ADK_MOUSE&px=1&section=5&ave_thresh=1&_ignoreionsscorebelow=20&report=0&_sigthreshold=0.05&_msresflags=1089&_msresflags2=2&percolate=-1&percolate_rt=0))  **338 - 348 579.3258 1156.6360 1156.6353 0 0 R.AGHYAASVIIR.R**  ([Ions score 25](http://10.139.25.109/mascot/cgi/peptide_view.pl?file=../data/20120413/F007792.dat&query=8425&hit=1&index=ADK_MOUSE&px=1&section=5&ave_thresh=1&_ignoreionsscorebelow=20&report=0&_sigthreshold=0.05&_msresflags=1089&_msresflags2=2&percolate=-1&percolate_rt=0))  8. [PGS1_MOUSE](http://10.139.25.109/mascot/cgi/protein_view.pl?file=../data/20120413/F007793.dat&hit=PGS1_MOUSE&db_idx=1&px=1&ave_thresh=1&_ignoreionsscorebelow=20&report=0&_sigthreshold=0.05&_msresflags=1089&_msresflags2=2&percolate=-1&percolate_rt=0)    **Mass:** 42069    **Score:** 794   **Matches:** 15(15)  **Sequences:** 11(11)  Biglycan  Sequence Coverage: **35%**; Matched peptides shown in **Bold Red**  **1** MCPLWLLTLL LALSQALPFE QKGFWDFTLD DGLLMMNDEE ASGSDTTSGV PDLDSVTPTF SAMCPFGCHC HLR**VVQCSDL GLK**TVPK**EIS PDTTLLDLQN**  **101 NDISELRKDD FKGLQHLYAL VLVNNK**ISKI HEKAFSPLRK LQKLYISK**NH LVEIPPNLPS SLVELR**IHDN RIRKVPK**GVF SGLR**NMNCIE MGGNPLENSG  **201** FEPGAFDGLK LNYLRISEAK LTGIPKDLPE TLNELHLDHN K**IQAIELEDL LR**YSKLYR**LG LGHNQIRMIE NGSLSFLPTL R**ELHLDNNKL SR**VPAGLPDL**  **301 K**LLQVVYLHS NNITK**VGIND FCPMGFGVK**R AYYNGISLFN NPVPYWEVQP ATFRCVTDRL AIQFGNYKK    **Start - End Observed Mr(expt) Mr(calc) ppm Miss Sequence**  **74 - 83 559.7966 1117.5786 1117.5802 -1 0 R.VVQCSDLGLK.T**  ([Ions score 63](http://10.139.25.109/mascot/cgi/peptide_view.pl?file=../data/20120413/F007793.dat&query=14242&hit=1&index=PGS1_MOUSE&px=1&section=5&ave_thresh=1&_ignoreionsscorebelow=20&report=0&_sigthreshold=0.05&_msresflags=1089&_msresflags2=2&percolate=-1&percolate_rt=0))  **88 - 108 805.4186 2413.2324 2413.2336 0 1 K.EISPDTTLLDLQNNDISELRK.D** ([Ions score 41](http://10.139.25.109/mascot/cgi/peptide_view.pl?file=../data/20120413/F007793.dat&query=44038&hit=1&index=PGS1_MOUSE&px=1&section=5&ave_thresh=1&_ignoreionsscorebelow=20&report=0&_sigthreshold=0.05&_msresflags=1089&_msresflags2=2&percolate=-1&percolate_rt=0))  **109 - 126 696.3813 2086.1205 2086.1211 0 1 K.DDFKGLQHLYALVLVNNK.I**  ([Ions score 44](http://10.139.25.109/mascot/cgi/peptide_view.pl?file=../data/20120413/F007793.dat&query=41491&hit=1&index=PGS1_MOUSE&px=1&section=5&ave_thresh=1&_ignoreionsscorebelow=20&report=0&_sigthreshold=0.05&_msresflags=1089&_msresflags2=2&percolate=-1&percolate_rt=0))  **113 - 126 527.9742 1580.9007 1580.9038 -2 0 K.GLQHLYALVLVNNK.I**  ([Ions score 34](http://10.139.25.109/mascot/cgi/peptide_view.pl?file=../data/20120413/F007793.dat&query=30274&hit=1&index=PGS1_MOUSE&px=1&section=5&ave_thresh=1&_ignoreionsscorebelow=20&report=0&_sigthreshold=0.05&_msresflags=1089&_msresflags2=2&percolate=-1&percolate_rt=0))  **149 - 166 1014.0682 2026.1208 2026.1211 0 0 K.NHLVEIPPNLPSSLVELR.I**  ([Ions score 73](http://10.139.25.109/mascot/cgi/peptide_view.pl?file=../data/20120413/F007793.dat&query=40687&hit=1&index=PGS1_MOUSE&px=1&section=5&ave_thresh=1&_ignoreionsscorebelow=20&report=0&_sigthreshold=0.05&_msresflags=1089&_msresflags2=2&percolate=-1&percolate_rt=0))  **149 - 166 676.3815 2026.1211 2026.1211 0 0 K.NHLVEIPPNLPSSLVELR.I**  ([Ions score 73](http://10.139.25.109/mascot/cgi/peptide_view.pl?file=../data/20120413/F007793.dat&query=40688&hit=1&index=PGS1_MOUSE&px=1&section=5&ave_thresh=1&_ignoreionsscorebelow=20&report=0&_sigthreshold=0.05&_msresflags=1089&_msresflags2=2&percolate=-1&percolate_rt=0))  **178 - 184 368.2108 734.4071 734.4075 -1 0 K.GVFSGLR.N**  ([Ions score 38](http://10.139.25.109/mascot/cgi/peptide_view.pl?file=../data/20120413/F007793.dat&query=615&hit=1&index=PGS1_MOUSE&px=1&section=5&ave_thresh=1&_ignoreionsscorebelow=20&report=0&_sigthreshold=0.05&_msresflags=1089&_msresflags2=2&percolate=-1&percolate_rt=0))  **242 - 252 656.8762 1311.7378 1311.7398 -2 0 K.IQAIELEDLLR.Y**  ([Ions score 91](http://10.139.25.109/mascot/cgi/peptide_view.pl?file=../data/20120413/F007793.dat&query=23380&hit=1&index=PGS1_MOUSE&px=1&section=5&ave_thresh=1&_ignoreionsscorebelow=20&report=0&_sigthreshold=0.05&_msresflags=1089&_msresflags2=2&percolate=-1&percolate_rt=0))  **259 - 267 504.2897 1006.5648 1006.5672 -2 0 R.LGLGHNQIR.M**  ([Ions score 26](http://10.139.25.109/mascot/cgi/peptide_view.pl?file=../data/20120413/F007793.dat&query=9379&hit=1&index=PGS1_MOUSE&px=1&section=5&ave_thresh=1&_ignoreionsscorebelow=20&report=0&_sigthreshold=0.05&_msresflags=1089&_msresflags2=2&percolate=-1&percolate_rt=0))  **259 - 267 336.5297 1006.5672 1006.5672 0 0 R.LGLGHNQIR.M**  ([Ions score 28](http://10.139.25.109/mascot/cgi/peptide_view.pl?file=../data/20120413/F007793.dat&query=9381&hit=1&index=PGS1_MOUSE&px=1&section=5&ave_thresh=1&_ignoreionsscorebelow=20&report=0&_sigthreshold=0.05&_msresflags=1089&_msresflags2=2&percolate=-1&percolate_rt=0))  **259 - 267 504.2914 1006.5681 1006.5672 1 0 R.LGLGHNQIR.M**  ([Ions score 54](http://10.139.25.109/mascot/cgi/peptide_view.pl?file=../data/20120413/F007793.dat&query=9383&hit=1&index=PGS1_MOUSE&px=1&section=5&ave_thresh=1&_ignoreionsscorebelow=20&report=0&_sigthreshold=0.05&_msresflags=1089&_msresflags2=2&percolate=-1&percolate_rt=0))  **268 - 281 789.9142 1577.8138 1577.8123 1 0 R.MIENGSLSFLPTLR.E**  Deamidated (NQ) ([Ions score 70](http://10.139.25.109/mascot/cgi/peptide_view.pl?file=../data/20120413/F007793.dat&query=30190&hit=1&index=PGS1_MOUSE&px=1&section=5&ave_thresh=1&_ignoreionsscorebelow=20&report=0&_sigthreshold=0.05&_msresflags=1089&_msresflags2=2&percolate=-1&percolate_rt=0))  **293 - 301 455.2738 908.5330 908.5331 0 0 R.VPAGLPDLK.L**  ([Ions score 43](http://10.139.25.109/mascot/cgi/peptide_view.pl?file=../data/20120413/F007793.dat&query=5450&hit=1&index=PGS1_MOUSE&px=1&section=5&ave_thresh=1&_ignoreionsscorebelow=20&report=0&_sigthreshold=0.05&_msresflags=1089&_msresflags2=2&percolate=-1&percolate_rt=0))  **316 - 329 770.8693 1539.7240 1539.7214 2 0 K.VGINDFCPMGFGVK.R**  ([Ions score 63](http://10.139.25.109/mascot/cgi/peptide_view.pl?file=../data/20120413/F007793.dat&query=28995&hit=1&index=PGS1_MOUSE&px=1&section=5&ave_thresh=1&_ignoreionsscorebelow=20&report=0&_sigthreshold=0.05&_msresflags=1089&_msresflags2=2&percolate=-1&percolate_rt=0))  **316 - 329 778.8640 1555.7135 1555.7164 -2 0 K.VGINDFCPMGFGVK.R**  Oxidation (M) ([Ions score 53](http://10.139.25.109/mascot/cgi/peptide_view.pl?file=../data/20120413/F007793.dat&query=29482&hit=1&index=PGS1_MOUSE&px=1&section=5&ave_thresh=1&_ignoreionsscorebelow=20&report=0&_sigthreshold=0.05&_msresflags=1089&_msresflags2=2&percolate=-1&percolate_rt=0))  9. [CD248_MOUSE](http://10.139.25.109/mascot/cgi/protein_view.pl?file=../data/20120413/F007793.dat&hit=CD248_MOUSE&db_idx=1&px=1&ave_thresh=1&_ignoreionsscorebelow=20&report=0&_sigthreshold=0.05&_msresflags=1089&_msresflags2=2&percolate=-1&percolate_rt=0)    **Mass:** 83756    **Score:** 108     **Matches:** 4(4)  **Sequences:** 3(3)  Endosialin  Sequence Coverage: **3%**; Matched peptides shown in **Bold Red**  **1** MLLRLLLAWV AAVPALGQVP WTPEPRAACG PSSCYALFPR RR**TFLEAWR**A CR**ELGGNLAT PR**TPEEAQRV DSLVGVGPAN GLLWIGLQRQ ARQCQPQRPL  **101** RGFIWTTGDQ DTAFTNWAQP ATEGPCPAQR CAALEASGEH RWLEGSCTLA VDGYLCQFGF EGACPALPLE VGQAGPAVYT TPFNLVSSEF EWLPFGSVAA  **201** VQCQAGRGAS LLCVKQPSGG VGWSQTGPLC PGTGCGPDNG GCEHECVEEV DGAVSCRCSE GFRLAADGHS CEDPCAQAPC EQQCEPGGPQ GYSCHCRLGF  **301** RPAEDDPHRC VDTDECQIAG VCQQMCVNYV GGFECYCSEG HELEADGISC SPAGAMGAQA SQDLRDELLD DGEEGEDEEE PWEDFDGTWT EEQGILWLAP  **401** THPPDFGLPY RPNFPQDGEP QRLHLEPTWP PPLSAPRGPY HSSVVSATRP MVISATRPTL PSAHKTSVIS ATRPPLSPVH PPAMAPATPP AVFSEHQIPK  **501** IKANYPDLPF GHKPGITSAT HPARSPPYQP PIISTNYPQV FPPHQAPMSP DTHTITYLPP VPPHLDPGDT TSKAHQHPLL PDAPGIRTQA PQLSVSALQP  **601** PLPTNSRSSV HETPVPAANQ PPAFPSSPLP PQRPTNQTSS ISPTHSYSRA PLVPREGVPS PKSVPQLPSV PSTAAPTALA ESGLAGQSQR DDRWLLVALL  **701** VPTCVFLVVL LALGIVYCTR CGSHAPNKRI TDCYRWVTHA GNKSSTEPMP PR**GSLTGVQT CR**TSV  **Start - End Observed Mr(expt) Mr(calc) ppm Miss Sequence**  **43 - 49 461.7434 921.4723 921.4708 2 0 R.TFLEAWR.A**  ([Ions score 29](http://10.139.25.109/mascot/cgi/peptide_view.pl?file=../data/20120413/F007793.dat&query=5944&hit=1&index=CD248_MOUSE&px=1&section=5&ave_thresh=1&_ignoreionsscorebelow=20&report=0&_sigthreshold=0.05&_msresflags=1089&_msresflags2=2&percolate=-1&percolate_rt=0))  **53 - 62 514.2814 1026.5482 1026.5458 2 0 R.ELGGNLATPR.T**  ([Ions score 40](http://10.139.25.109/mascot/cgi/peptide_view.pl?file=../data/20120413/F007793.dat&query=10127&hit=1&index=CD248_MOUSE&px=1&section=5&ave_thresh=1&_ignoreionsscorebelow=20&report=0&_sigthreshold=0.05&_msresflags=1089&_msresflags2=2&percolate=-1&percolate_rt=0))  **753 - 762 539.7670 1077.5194 1077.5237 -4 0 R.GSLTGVQTCR.T**  ([Ions score 39](http://10.139.25.109/mascot/cgi/peptide_view.pl?file=../data/20120413/F007793.dat&query=12367&hit=1&index=CD248_MOUSE&px=1&section=5&ave_thresh=1&_ignoreionsscorebelow=20&report=0&_sigthreshold=0.05&_msresflags=1089&_msresflags2=2&percolate=-1&percolate_rt=0))  10. [CO6A1_MOUSE](http://10.139.25.109/mascot/cgi/protein_view.pl?file=../data/20120413/F007793.dat&hit=CO6A1_MOUSE&db_idx=1&px=1&ave_thresh=1&_ignoreionsscorebelow=20&report=0&_sigthreshold=0.05&_msresflags=1089&_msresflags2=2&percolate=-1&percolate_rt=0)    **Mass:** 109562   **Score:** 1307   **Matches:** 26(26) **Sequences:** 18(18)  Collagen alpha-1(VI) chain  Sequence Coverage: **20%**; Matched peptides shown in **Bold Red**  **1** MRLAHALLPL LLQACWVATQ DIQGSKAIAF QDCPVDLFFV LDTSESVALR **LKPYGALVDK VK**SFTKR**FID NLR**DRYYRCD RNLVWNAGAL HYSDEVEIIR  **101** GLTRMPSGRD ELKASVDAVK YFGKGTYTDC AIKK**GLEELL IGGSHLKENK** YLIVVTDGHP LEGYKEPCGG LEDAVNEAKH LGIK**VFSVAI TPDHLEPR**LS  **201** IIATDHTYRR NFTAADWGHS R**DAEEVISQT IDTIVDMIKN NVEQVCCSFE CQAAR**GPPGP RGDPGYEGER GKPGLPGEKG EAGDPGRPGD LGPVGYQGMK  **301** GEKGSRGEKG SRGPKGYKGE KGKRGIDGVD GMKGETGYPG LPGCKGSPGF DGIQGPPGPK GDAGAFGMKG EKGEAGADGE AGRPGNSGSP GDEGDPGEPG  **401** PPGEKGEAGD EGNAGPDGAP GERGGPGERG PRGTPGVRGP R**GDPGEAGPQ GDQGR**EGPVG IPGDSGEAGP IGPKGYRGDE GPPGPEGLRG APGPVGPPGD  **501** PGLMGERGED GPPGNGTEGF PGFPGYPGNR GPPGLNGTKG YPGLKGDEGE VGDPGEDNND ISPRGVKGAK GYRGPEGPQG PPGHVGPPGP DECEILDIIM  **601** KMCSCCECTC GPIDILFVLD SSESIGLQNF EIAKDFIIKV IDR**LSKDELV K**FEPGQSHAG VVQYSHNQMQ EHVDMRSPNV RNAQDFKEAV KKLQWMAGGT  **701** FTGEALQYTR DRLLPPTQNN R**IALVITDGR** SDTQR**DTTPL SVLCGADIQV VSVGIK**DVFG FVAGSDQLNV ISCQGLSQGR PGISLVKENY AELLDDGFLK  **801** NITAQICIDK KCPDYTCPIT FSSPADITIL LDSSASVGSH NFETTKVFAK RLAERFLSAG R**ADPSQDVRV AVVQYSGQGQ QQPGR**AALQF LQNYTVLASS  **901** VDSMDFINDA TDVNDALSYV TRFYREASSG ATKKR**VLLFS DGNSQGATAE AIEK**AVQEAQ RAGIEIFVVV VGPQVNEPHI RVLVTGK**TAE YDVAFGER**HL  **1001** FR**VPNYQALL RGVLYQTVSR** KVALG  **Start - End Observed Mr(expt) Mr(calc) ppm Miss Sequence**  **51 - 60 368.5533 1102.6382 1102.6386 0 0 R.LKPYGALVDK.V**  ([Ions score 32](http://10.139.25.109/mascot/cgi/peptide_view.pl?file=../data/20120413/F007793.dat&query=13509&hit=1&index=CO6A1_MOUSE&px=1&section=5&ave_thresh=1&_ignoreionsscorebelow=20&report=0&_sigthreshold=0.05&_msresflags=1089&_msresflags2=2&percolate=-1&percolate_rt=0))  **51 - 60 552.3268 1102.6390 1102.6386 0 0 R.LKPYGALVDK.V**  ([Ions score 21](http://10.139.25.109/mascot/cgi/peptide_view.pl?file=../data/20120413/F007793.dat&query=13510&hit=1&index=CO6A1_MOUSE&px=1&section=5&ave_thresh=1&_ignoreionsscorebelow=20&report=0&_sigthreshold=0.05&_msresflags=1089&_msresflags2=2&percolate=-1&percolate_rt=0))  **51 - 62 333.4573 1329.8001 1329.8020 -1 1 R.LKPYGALVDKVK.S**  ([Ions score 25](http://10.139.25.109/mascot/cgi/peptide_view.pl?file=../data/20120413/F007793.dat&query=24048&hit=1&index=CO6A1_MOUSE&px=1&section=5&ave_thresh=1&_ignoreionsscorebelow=20&report=0&_sigthreshold=0.05&_msresflags=1089&_msresflags2=2&percolate=-1&percolate_rt=0))  **51 - 62 444.2749 1329.8029 1329.8020 1 1 R.LKPYGALVDKVK.S**  ([Ions score 34](http://10.139.25.109/mascot/cgi/peptide_view.pl?file=../data/20120413/F007793.dat&query=24049&hit=1&index=CO6A1_MOUSE&px=1&section=5&ave_thresh=1&_ignoreionsscorebelow=20&report=0&_sigthreshold=0.05&_msresflags=1089&_msresflags2=2&percolate=-1&percolate_rt=0))  **68 - 73 389.2167 776.4189 776.4181 1 0 R.FIDNLR.D**  ([Ions score 21](http://10.139.25.109/mascot/cgi/peptide_view.pl?file=../data/20120413/F007793.dat&query=1531&hit=1&index=CO6A1_MOUSE&px=1&section=5&ave_thresh=1&_ignoreionsscorebelow=20&report=0&_sigthreshold=0.05&_msresflags=1089&_msresflags2=2&percolate=-1&percolate_rt=0))  **135 - 147 683.3884 1364.7622 1364.7663 -3 0 K.GLEELLIGGSHLK.E**  ([Ions score 91](http://10.139.25.109/mascot/cgi/peptide_view.pl?file=../data/20120413/F007793.dat&query=24913&hit=1&index=CO6A1_MOUSE&px=1&section=5&ave_thresh=1&_ignoreionsscorebelow=20&report=0&_sigthreshold=0.05&_msresflags=1089&_msresflags2=2&percolate=-1&percolate_rt=0))  **135 - 147 455.9304 1364.7694 1364.7663 2 0 K.GLEELLIGGSHLK.E**  ([Ions score 55](http://10.139.25.109/mascot/cgi/peptide_view.pl?file=../data/20120413/F007793.dat&query=24914&hit=1&index=CO6A1_MOUSE&px=1&section=5&ave_thresh=1&_ignoreionsscorebelow=20&report=0&_sigthreshold=0.05&_msresflags=1089&_msresflags2=2&percolate=-1&percolate_rt=0))  **135 - 150 579.6563 1735.9469 1735.9468 0 1 K.GLEELLIGGSHLKENK.Y**  ([Ions score 45](http://10.139.25.109/mascot/cgi/peptide_view.pl?file=../data/20120413/F007793.dat&query=34415&hit=1&index=CO6A1_MOUSE&px=1&section=5&ave_thresh=1&_ignoreionsscorebelow=20&report=0&_sigthreshold=0.05&_msresflags=1089&_msresflags2=2&percolate=-1&percolate_rt=0))  **135 - 150 434.9946 1735.9492 1735.9468 1 1 K.GLEELLIGGSHLKENK.Y**  ([Ions score 25](http://10.139.25.109/mascot/cgi/peptide_view.pl?file=../data/20120413/F007793.dat&query=34416&hit=1&index=CO6A1_MOUSE&px=1&section=5&ave_thresh=1&_ignoreionsscorebelow=20&report=0&_sigthreshold=0.05&_msresflags=1089&_msresflags2=2&percolate=-1&percolate_rt=0))  **185 - 198 527.6191 1579.8356 1579.8358 0 0 K.VFSVAITPDHLEPR.L**  ([Ions score 37](http://10.139.25.109/mascot/cgi/peptide_view.pl?file=../data/20120413/F007793.dat&query=30240&hit=1&index=CO6A1_MOUSE&px=1&section=5&ave_thresh=1&_ignoreionsscorebelow=20&report=0&_sigthreshold=0.05&_msresflags=1089&_msresflags2=2&percolate=-1&percolate_rt=0))  **185 - 198 790.9263 1579.8381 1579.8358 1 0 K.VFSVAITPDHLEPR.L**  ([Ions score 51](http://10.139.25.109/mascot/cgi/peptide_view.pl?file=../data/20120413/F007793.dat&query=30242&hit=1&index=CO6A1_MOUSE&px=1&section=5&ave_thresh=1&_ignoreionsscorebelow=20&report=0&_sigthreshold=0.05&_msresflags=1089&_msresflags2=2&percolate=-1&percolate_rt=0))  **222 - 239 674.0103 2019.0075 2019.0082 0 0 R.DAEEVISQTIDTIVDMIK.N**  ([Ions score 39](http://10.139.25.109/mascot/cgi/peptide_view.pl?file=../data/20120413/F007793.dat&query=40564&hit=1&index=CO6A1_MOUSE&px=1&section=5&ave_thresh=1&_ignoreionsscorebelow=20&report=0&_sigthreshold=0.05&_msresflags=1089&_msresflags2=2&percolate=-1&percolate_rt=0))  **222 - 239 1018.5089 2035.0032 2035.0031 0 0 R.DAEEVISQTIDTIVDMIK.N**  Oxidation (M) ([Ions score 41](http://10.139.25.109/mascot/cgi/peptide_view.pl?file=../data/20120413/F007793.dat&query=40781&hit=1&index=CO6A1_MOUSE&px=1&section=5&ave_thresh=1&_ignoreionsscorebelow=20&report=0&_sigthreshold=0.05&_msresflags=1089&_msresflags2=2&percolate=-1&percolate_rt=0))  **222 - 239 679.3423 2035.0035 2035.0031 0 0 R.DAEEVISQTIDTIVDMIK.N**  Oxidation (M) ([Ions score 53](http://10.139.25.109/mascot/cgi/peptide_view.pl?file=../data/20120413/F007793.dat&query=40789&hit=1&index=CO6A1_MOUSE&px=1&section=5&ave_thresh=1&_ignoreionsscorebelow=20&report=0&_sigthreshold=0.05&_msresflags=1089&_msresflags2=2&percolate=-1&percolate_rt=0))  **240 - 255 986.4082 1970.8008 1970.8033 -1 0 K.NNVEQVCCSFECQAAR.G**  ([Ions score 92](http://10.139.25.109/mascot/cgi/peptide_view.pl?file=../data/20120413/F007793.dat&query=39820&hit=1&index=CO6A1_MOUSE&px=1&section=5&ave_thresh=1&_ignoreionsscorebelow=20&report=0&_sigthreshold=0.05&_msresflags=1089&_msresflags2=2&percolate=-1&percolate_rt=0))  **442 - 455 670.7952 1339.5759 1339.5753 0 0 R.GDPGEAGPQGDQGR.E**  ([Ions score 82](http://10.139.25.109/mascot/cgi/peptide_view.pl?file=../data/20120413/F007793.dat&query=24277&hit=1&index=CO6A1_MOUSE&px=1&section=5&ave_thresh=1&_ignoreionsscorebelow=20&report=0&_sigthreshold=0.05&_msresflags=1089&_msresflags2=2&percolate=-1&percolate_rt=0))  **644 - 651 466.2754 930.5362 930.5386 -3 1 R.LSKDELVK.F**  ([Ions score 27](http://10.139.25.109/mascot/cgi/peptide_view.pl?file=../data/20120413/F007793.dat&query=6318&hit=1&index=CO6A1_MOUSE&px=1&section=5&ave_thresh=1&_ignoreionsscorebelow=20&report=0&_sigthreshold=0.05&_msresflags=1089&_msresflags2=2&percolate=-1&percolate_rt=0))  **722 - 730 479.2899 956.5652 956.5655 0 0 R.IALVITDGR.S**  ([Ions score 51](http://10.139.25.109/mascot/cgi/peptide_view.pl?file=../data/20120413/F007793.dat&query=7315&hit=1&index=CO6A1_MOUSE&px=1&section=5&ave_thresh=1&_ignoreionsscorebelow=20&report=0&_sigthreshold=0.05&_msresflags=1089&_msresflags2=2&percolate=-1&percolate_rt=0))  **736 - 756 1086.5827 2171.1498 2171.1508 0 0 R.DTTPLSVLCGADIQVVSVGIK.D** ([Ions score 32](http://10.139.25.109/mascot/cgi/peptide_view.pl?file=../data/20120413/F007793.dat&query=42336&hit=1&index=CO6A1_MOUSE&px=1&section=5&ave_thresh=1&_ignoreionsscorebelow=20&report=0&_sigthreshold=0.05&_msresflags=1089&_msresflags2=2&percolate=-1&percolate_rt=0))  **862 - 869 444.2136 886.4127 886.4145 -2 0 R.ADPSQDVR.V**  ([Ions score 22](http://10.139.25.109/mascot/cgi/peptide_view.pl?file=../data/20120413/F007793.dat&query=4633&hit=1&index=CO6A1_MOUSE&px=1&section=5&ave_thresh=1&_ignoreionsscorebelow=20&report=0&_sigthreshold=0.05&_msresflags=1089&_msresflags2=2&percolate=-1&percolate_rt=0))  **870 - 885 567.9600 1700.8582 1700.8595 -1 0 R.VAVVQYSGQGQQQPGR.A**  ([Ions score 45](http://10.139.25.109/mascot/cgi/peptide_view.pl?file=../data/20120413/F007793.dat&query=33516&hit=1&index=CO6A1_MOUSE&px=1&section=5&ave_thresh=1&_ignoreionsscorebelow=20&report=0&_sigthreshold=0.05&_msresflags=1089&_msresflags2=2&percolate=-1&percolate_rt=0))  **870 - 885 851.4373 1700.8601 1700.8595 0 0 R.VAVVQYSGQGQQQPGR.A**  ([Ions score 119](http://10.139.25.109/mascot/cgi/peptide_view.pl?file=../data/20120413/F007793.dat&query=33517&hit=1&index=CO6A1_MOUSE&px=1&section=5&ave_thresh=1&_ignoreionsscorebelow=20&report=0&_sigthreshold=0.05&_msresflags=1089&_msresflags2=2&percolate=-1&percolate_rt=0))  **936 - 954 975.4953 1948.9760 1948.9742 1 0 R.VLLFSDGNSQGATAEAIEK.A**  ([Ions score 113](http://10.139.25.109/mascot/cgi/peptide_view.pl?file=../data/20120413/F007793.dat&query=39338&hit=1&index=CO6A1_MOUSE&px=1&section=5&ave_thresh=1&_ignoreionsscorebelow=20&report=0&_sigthreshold=0.05&_msresflags=1089&_msresflags2=2&percolate=-1&percolate_rt=0))  **988 - 998 629.2926 1256.5706 1256.5673 3 0 K.TAEYDVAFGER.H**  ([Ions score 37](http://10.139.25.109/mascot/cgi/peptide_view.pl?file=../data/20120413/F007793.dat&query=21138&hit=1&index=CO6A1_MOUSE&px=1&section=5&ave_thresh=1&_ignoreionsscorebelow=20&report=0&_sigthreshold=0.05&_msresflags=1089&_msresflags2=2&percolate=-1&percolate_rt=0))  **1003 - 1011 537.3098 1072.6051 1072.6029 2 0 R.VPNYQALLR.G**  ([Ions score 56](http://10.139.25.109/mascot/cgi/peptide_view.pl?file=../data/20120413/F007793.dat&query=12164&hit=1&index=CO6A1_MOUSE&px=1&section=5&ave_thresh=1&_ignoreionsscorebelow=20&report=0&_sigthreshold=0.05&_msresflags=1089&_msresflags2=2&percolate=-1&percolate_rt=0))  **1012 - 1020 511.7847 1021.5548 1021.5556 -1 0 R.GVLYQTVSR.K**  ([Ions score 61](http://10.139.25.109/mascot/cgi/peptide_view.pl?file=../data/20120413/F007793.dat&query=9947&hit=1&index=CO6A1_MOUSE&px=1&section=5&ave_thresh=1&_ignoreionsscorebelow=20&report=0&_sigthreshold=0.05&_msresflags=1089&_msresflags2=2&percolate=-1&percolate_rt=0))  11. [GAS2_MOUSE](http://10.139.25.109/mascot/cgi/protein_view.pl?file=../data/20120413/F007793.dat&hit=GAS2_MOUSE&db_idx=1&px=1&ave_thresh=1&_ignoreionsscorebelow=20&report=0&_sigthreshold=0.05&_msresflags=1089&_msresflags2=2&percolate=-1&percolate_rt=0)    **Mass:** 35505    **Score:** 1021   **Matches:** 21(21)  **Sequences:** 16(16)  Growth arrest-specific protein 2  Sequence Coverage: **64%**; Matched peptides shown in **Bold Red**  **1** MMCTALSPKV R**SGPGLSDMH QYSQWLASRH EANLLPMKED LALWLTNLLG KEITAETFME KLDNGALLCQ LAATVQEKFK ESMDANKPAK** TLPLKKIPCK  **101** **ASAPSGSFFA RDNTANFLSW CR**DLGVDETC LFESEGLVLH KQPR**EVCLCL LELGR**IAAR**Y GVEPPGLIKL EKEIEQEETL SAPSPSPSPS SK**SSGKK**STG**  **201 NLLDDAVKR**I SEDPPCKCPT K**FCVER**LSQG RYRVGEKILF IRMLHNKHVM VR**VGGGWETF AGYLLK**HDPC R**MLQISR**VDG KTSPVQSK**SP TLKDMNPDNY**  **301 LVVSATYK**AK KEIK  **Start - End Observed Mr(expt) Mr(calc) ppm Miss Sequence**  **12 - 29 673.9830 2018.9256 2018.9269 1 0 R.SGPGLSDMHQYSQWLASR.H**  ([Ions score 68](http://10.139.25.109/mascot/cgi/peptide_view.pl?file=../data/20120413/F007793.dat&query=40559&hit=1&index=GAS2_MOUSE&px=1&section=5&ave_thresh=1&_ignoreionsscorebelow=20&report=0&_sigthreshold=0.05&_msresflags=1089&_msresflags2=2&percolate=-1&percolate_rt=0))  **30 - 51 634.5964 2534.3543 2534.3333 6 1 R.HEANLLPMKEDLALWLTNLLGK.E** Oxidation (M) ([Ions score 42](http://10.139.25.109/mascot/cgi/peptide_view.pl?file=../data/20120413/F007793.dat&query=44843&hit=1&index=GAS2_MOUSE&px=1&section=5&ave_thresh=1&_ignoreionsscorebelow=20&report=0&_sigthreshold=0.05&_msresflags=1089&_msresflags2=2&percolate=-1&percolate_rt=0))  **52 - 61 599.7859 1197.5572 1197.5587 -1 0 K.EITAETFMEK.L**  ([Ions score 28](http://10.139.25.109/mascot/cgi/peptide_view.pl?file=../data/20120413/F007793.dat&query=18199&hit=1&index=GAS2_MOUSE&px=1&section=5&ave_thresh=1&_ignoreionsscorebelow=20&report=0&_sigthreshold=0.05&_msresflags=1089&_msresflags2=2&percolate=-1&percolate_rt=0))  **52 - 61 607.7847 1213.5549 1213.5536 1 0 K.EITAETFMEK.L**  Oxidation (M) ([Ions score 35](http://10.139.25.109/mascot/cgi/peptide_view.pl?file=../data/20120413/F007793.dat&query=19094&hit=1&index=GAS2_MOUSE&px=1&section=5&ave_thresh=1&_ignoreionsscorebelow=20&report=0&_sigthreshold=0.05&_msresflags=1089&_msresflags2=2&percolate=-1&percolate_rt=0))  **62 - 78 615.3234 1842.9485 1842.9509 -1 0 K.LDNGALLCQLAATVQEK.F**  ([Ions score 43](http://10.139.25.109/mascot/cgi/peptide_view.pl?file=../data/20120413/F007793.dat&query=37258&hit=1&index=GAS2_MOUSE&px=1&section=5&ave_thresh=1&_ignoreionsscorebelow=20&report=0&_sigthreshold=0.05&_msresflags=1089&_msresflags2=2&percolate=-1&percolate_rt=0))  **62 - 78 922.4840 1842.9535 1842.9509 1 0 K.LDNGALLCQLAATVQEK.F**  ([Ions score 87](http://10.139.25.109/mascot/cgi/peptide_view.pl?file=../data/20120413/F007793.dat&query=37259&hit=1&index=GAS2_MOUSE&px=1&section=5&ave_thresh=1&_ignoreionsscorebelow=20&report=0&_sigthreshold=0.05&_msresflags=1089&_msresflags2=2&percolate=-1&percolate_rt=0))  **79 - 90 461.2302 1380.6688 1380.6707 -1 1 K.FKESMDANKPAK.T**  Oxidation (M) ([Ions score 49](http://10.139.25.109/mascot/cgi/peptide_view.pl?file=../data/20120413/F007793.dat&query=25216&hit=1&index=GAS2_MOUSE&px=1&section=5&ave_thresh=1&_ignoreionsscorebelow=20&report=0&_sigthreshold=0.05&_msresflags=1089&_msresflags2=2&percolate=-1&percolate_rt=0))  **79 - 90 691.3428 1380.6710 1380.6707 0 1 K.FKESMDANKPAK.T**  Oxidation (M) ([Ions score 77](http://10.139.25.109/mascot/cgi/peptide_view.pl?file=../data/20120413/F007793.dat&query=25217&hit=1&index=GAS2_MOUSE&px=1&section=5&ave_thresh=1&_ignoreionsscorebelow=20&report=0&_sigthreshold=0.05&_msresflags=1089&_msresflags2=2&percolate=-1&percolate_rt=0))  **79 - 90 346.1752 1380.6717 1380.6707 1 1 K.FKESMDANKPAK.T**  Oxidation (M) ([Ions score 22](http://10.139.25.109/mascot/cgi/peptide_view.pl?file=../data/20120413/F007793.dat&query=25218&hit=1&index=GAS2_MOUSE&px=1&section=5&ave_thresh=1&_ignoreionsscorebelow=20&report=0&_sigthreshold=0.05&_msresflags=1089&_msresflags2=2&percolate=-1&percolate_rt=0))  **101 - 111 549.2722 1096.5298 1096.5302 0 0 K.ASAPSGSFFAR.D**  ([Ions score 47](http://10.139.25.109/mascot/cgi/peptide_view.pl?file=../data/20120413/F007793.dat&query=13222&hit=1&index=GAS2_MOUSE&px=1&section=5&ave_thresh=1&_ignoreionsscorebelow=20&report=0&_sigthreshold=0.05&_msresflags=1089&_msresflags2=2&percolate=-1&percolate_rt=0))  **112 - 122 692.3067 1382.5988 1382.6037 -4 0 R.DNTANFLSWCR.D**  ([Ions score 68](http://10.139.25.109/mascot/cgi/peptide_view.pl?file=../data/20120413/F007793.dat&query=25278&hit=1&index=GAS2_MOUSE&px=1&section=5&ave_thresh=1&_ignoreionsscorebelow=20&report=0&_sigthreshold=0.05&_msresflags=1089&_msresflags2=2&percolate=-1&percolate_rt=0))  **145 - 155 681.3497 1360.6848 1360.6843 0 0 R.EVCLCLLELGR.I**  ([Ions score 54](http://10.139.25.109/mascot/cgi/peptide_view.pl?file=../data/20120413/F007793.dat&query=24803&hit=1&index=GAS2_MOUSE&px=1&section=5&ave_thresh=1&_ignoreionsscorebelow=20&report=0&_sigthreshold=0.05&_msresflags=1089&_msresflags2=2&percolate=-1&percolate_rt=0))  **160 - 169 536.8041 1071.5936 1071.5964 -3 0 R.YGVEPPGLIK.L**  ([Ions score 32](http://10.139.25.109/mascot/cgi/peptide_view.pl?file=../data/20120413/F007793.dat&query=12099&hit=1&index=GAS2_MOUSE&px=1&section=5&ave_thresh=1&_ignoreionsscorebelow=20&report=0&_sigthreshold=0.05&_msresflags=1089&_msresflags2=2&percolate=-1&percolate_rt=0))  **160 - 172 481.6127 1441.8162 1441.8180 -1 1 R.YGVEPPGLIKLEK.E**  ([Ions score 21](http://10.139.25.109/mascot/cgi/peptide_view.pl?file=../data/20120413/F007793.dat&query=26640&hit=1&index=GAS2_MOUSE&px=1&section=5&ave_thresh=1&_ignoreionsscorebelow=20&report=0&_sigthreshold=0.05&_msresflags=1089&_msresflags2=2&percolate=-1&percolate_rt=0))  **173 - 192 1050.5027 2098.9898 2098.9906 0 0 K.EIEQEETLSAPSPSPSPSSK.S**([Ions score 58](http://10.139.25.109/mascot/cgi/peptide_view.pl?file=../data/20120413/F007793.dat&query=41650&hit=1&index=GAS2_MOUSE&px=1&section=5&ave_thresh=1&_ignoreionsscorebelow=20&report=0&_sigthreshold=0.05&_msresflags=1089&_msresflags2=2&percolate=-1&percolate_rt=0))  **198 - 209 644.8431 1287.6716 1287.6783 -5 1 K.STGNLLDDAVKR.I**  ([Ions score 77](http://10.139.25.109/mascot/cgi/peptide_view.pl?file=../data/20120413/F007793.dat&query=22495&hit=1&index=GAS2_MOUSE&px=1&section=5&ave_thresh=1&_ignoreionsscorebelow=20&report=0&_sigthreshold=0.05&_msresflags=1089&_msresflags2=2&percolate=-1&percolate_rt=0))  **198 - 209 430.2332 1287.6776 1287.6783 -1 1 K.STGNLLDDAVKR.I**  ([Ions score 41](http://10.139.25.109/mascot/cgi/peptide_view.pl?file=../data/20120413/F007793.dat&query=22499&hit=1&index=GAS2_MOUSE&px=1&section=5&ave_thresh=1&_ignoreionsscorebelow=20&report=0&_sigthreshold=0.05&_msresflags=1089&_msresflags2=2&percolate=-1&percolate_rt=0))  **222 - 226 355.6679 709.3213 709.3217 -1 0 K.FCVER.L**  ([Ions score 24](http://10.139.25.109/mascot/cgi/peptide_view.pl?file=../data/20120413/F007793.dat&query=168&hit=1&index=GAS2_MOUSE&px=1&section=5&ave_thresh=1&_ignoreionsscorebelow=20&report=0&_sigthreshold=0.05&_msresflags=1089&_msresflags2=2&percolate=-1&percolate_rt=0))  **253 - 266 749.3886 1496.7627 1496.7664 -2 0 R.VGGGWETFAGYLLK.H**  ([Ions score 95](http://10.139.25.109/mascot/cgi/peptide_view.pl?file=../data/20120413/F007793.dat&query=27914&hit=1&index=GAS2_MOUSE&px=1&section=5&ave_thresh=1&_ignoreionsscorebelow=20&report=0&_sigthreshold=0.05&_msresflags=1089&_msresflags2=2&percolate=-1&percolate_rt=0))  **272 - 277 382.2104 762.4062 762.4058 0 0 R.MLQISR.V**  Oxidation (M) ([Ions score 27](http://10.139.25.109/mascot/cgi/peptide_view.pl?file=../data/20120413/F007793.dat&query=1206&hit=1&index=GAS2_MOUSE&px=1&section=5&ave_thresh=1&_ignoreionsscorebelow=20&report=0&_sigthreshold=0.05&_msresflags=1089&_msresflags2=2&percolate=-1&percolate_rt=0))  **289 - 308 758.0424 2271.1038 2271.0860 7 1 K.SPTLKDMNPDNYLVVSATYK.A**  Oxidation (M) ([Ions score 26](http://10.139.25.109/mascot/cgi/peptide_view.pl?file=../data/20120413/F007793.dat&query=43088&hit=1&index=GAS2_MOUSE&px=1&section=5&ave_thresh=1&_ignoreionsscorebelow=20&report=0&_sigthreshold=0.05&_msresflags=1089&_msresflags2=2&percolate=-1&percolate_rt=0))  12. [TOP2B_MOUSE](http://10.139.25.109/mascot/cgi/protein_view.pl?file=../data/20120413/F007791.dat&hit=TOP2B_MOUSE&db_idx=1&px=1&ave_thresh=1&_ignoreionsscorebelow=20&report=0&_sigthreshold=0.05&_msresflags=1089&_msresflags2=2&percolate=-1&percolate_rt=0)    **Mass:** 182707   **Score:** 501    **Matches:** 9(9)  **Sequences:** 9(9)  DNA topoisomerase 2-beta  Sequence Coverage: **7%**; Matched peptides shown in **Bold Red**  **1** MAK**SSLAGSD GALTWVNNAT K**KEELETANK NDSTKKLSVE RVYQKKTQLE HILLRPDTYI GSVEPLTQLM WVYDEDVGMN CREVTFVPGL YK**IFDEILVN**  **101 AADNK**QRDKN MTCIKVSIDP ESNIISIWNN GKGIPVVEHK VEK**VYVPALI FGQLLTSSNY DDDEKK**VTGG RNGYGAKLCN IFSTKFTVET ACKEYKHSFK  **201** QTWMNNMMKT SEAKIKHFDG EDYTCITFQP DLSKFKMEKL DKDIVALMTR RAYDLAGSCK GVKVMFNGKK LPVNGFRSYV DLYVKDKLDE TGVALKVIHE  **301** LANERWDVCL TLSEKGFQQI SFVNSIATTK GGRHVDYVVD QVVSKLIEVV KKKNKAGVSV KPFQVKNHIW VFINCLIENP TFDSQTKENM TLQPKSFGSK  **401** CQLSEKFFK**A ASNCGIVESI LNWVK**FKAQT QLNKKCSSVK YSKIKGIPKL DDANDAGGKH SLECTLILTE GDSAK**SLAVS GLGVIGR**DRY GVFPLRGKIL  **501** NVREASHKQI MENAEINNII KIVGLQYKKS YDDAESLKTL RYGKIMIMTD QDQDGSHIKG LLINFIHHNW PSLLKHGFLE EFITPIVKAS KNKQELSFYS  **601** IPEFDEWKKH IENQKAWKIK YYKGLGTSTA KEAKEYFADM ERHRILFRYA GPEDDAAITL AFSKKKIDDR KEWLTNFMED RRQRRLHGLP EQFLYGTATK  **701** **HLTYNDFINK** ELILFSNSDN ERSIPSLVDG FKPGQRKVLF TCFKRNDKRE VKVAQLAGSV AEMSAYHHGE QALMMTIVNL AQNFVGSNNI NLLQPIGQFG  **801** TRLHGGKDAA SPR**YIFTMLS SLAR**LLFPAV DDNLLKFLYD DNQRVEPEWY IPIIPMVLIN GAEGIGTGWA CKLPNYDARE IVNNVRRMLE GLDPHPMLPN  **901** YKNFKGTIQE LGQNQYAVSG EIFVVDRNTV EITELPVRTW TQVYKEQVLE PMLNGTDKTP ALISDYKEYH TDTTVKFVVK MTEEKLAQAE AAGLHKVFKL  **1001** QTTLTCNSMV LFDHMGCLKK YETVQDILKE FFDLRLSYYG LRKEWLVGML GAESTKLNNQ ARFILEKIQG KITIENRSKK **DLIQMLVQR**G YESDPVKAWK  **1101** EAQEKAAEEE DSQNQHDDSS SDSGTPSGPD FNYILNMSLW SLTKEKVEEL IKQRDTKGRE VNDLKRKSPS DLWKEDLAAF VEELDKVEAQ EREDILAGMS  **1201** GKAIKGKVGK PKVKKLQLEE TMPSPYGRRI VPEITAMKAD ASRKLLKKKK GDPDTTVVKV EFDEEFSGTP AEGTGEETLT PSAPVNKGPK PKREKKEPGT  **1301** RVRKTPTSTG KTNAKKVKKR NPWSDDESKS ESDLEEAEPV VIPRDSLLRR AAAERPKYTF DFSEEEDDDA AAADDSNDLE ELKVKASPIT NDGEDEFVPS  **1401** DGLDKDEYAF SSGKSKATPE KSSNDKK**SQD FGNLFSFPSY SQK**SEDDSAK FDSNEEDTAS VFAPSFGLKQ TDKLPSKTVA AKKGKPPSDT APKAKRAPKQ  **1501** KKIVETINSD SDSEFGIPKK TTTPKGKGRG AKKRKASGSE NEGDYNPGRK PSKTASKKPK KTSFDQDSDV DIFPSDFTSE PPALPRTGRA RKEVKYFAES  **1601** DEEEDVDFAM FN  **Start - End Observed Mr(expt) Mr(calc) ppm Miss Sequence**  **4 - 21 896.4477 1790.8798 1790.8799 0 0 K.SSLAGSDGALTWVNNATK.K**  ([Ions score 28](http://10.139.25.109/mascot/cgi/peptide_view.pl?file=../data/20120413/F007791.dat&query=33177&hit=1&index=TOP2B_MOUSE&px=1&section=5&ave_thresh=1&_ignoreionsscorebelow=20&report=0&_sigthreshold=0.05&_msresflags=1089&_msresflags2=2&percolate=-1&percolate_rt=0))  **93 - 105 731.3814 1460.7483 1460.7511 -2 0 K.IFDEILVNAADNK.Q**  ([Ions score 81](http://10.139.25.109/mascot/cgi/peptide_view.pl?file=../data/20120413/F007791.dat&query=21431&hit=1&index=TOP2B_MOUSE&px=1&section=5&ave_thresh=1&_ignoreionsscorebelow=20&report=0&_sigthreshold=0.05&_msresflags=1089&_msresflags2=2&percolate=-1&percolate_rt=0))  **144 - 166 872.4463 2614.3155 2614.3166 0 1 K.VYVPALIFGQLLTSSNYDDDEKK.V** ([Ions score 23](http://10.139.25.109/mascot/cgi/peptide_view.pl?file=../data/20120413/F007791.dat&query=49556&hit=1&index=TOP2B_MOUSE&px=1&section=5&ave_thresh=1&_ignoreionsscorebelow=20&report=0&_sigthreshold=0.05&_msresflags=1089&_msresflags2=2&percolate=-1&percolate_rt=0))  **410 - 425 880.9563 1759.8980 1759.8927 3 0 K.AASNCGIVESILNWVK.F**  ([Ions score 81](http://10.139.25.109/mascot/cgi/peptide_view.pl?file=../data/20120413/F007791.dat&query=31918&hit=1&index=TOP2B_MOUSE&px=1&section=5&ave_thresh=1&_ignoreionsscorebelow=20&report=0&_sigthreshold=0.05&_msresflags=1089&_msresflags2=2&percolate=-1&percolate_rt=0))  **476 - 487 564.8391 1127.6637 1127.6663 -2 0 K.SLAVSGLGVIGR.D**  ([Ions score 71](http://10.139.25.109/mascot/cgi/peptide_view.pl?file=../data/20120413/F007791.dat&query=9204&hit=1&index=TOP2B_MOUSE&px=1&section=5&ave_thresh=1&_ignoreionsscorebelow=20&report=0&_sigthreshold=0.05&_msresflags=1089&_msresflags2=2&percolate=-1&percolate_rt=0))  **701 - 710 632.8237 1263.6328 1263.6248 6 0 K.HLTYNDFINK.E**  ([Ions score 46](http://10.139.25.109/mascot/cgi/peptide_view.pl?file=../data/20120413/F007791.dat&query=15095&hit=1&index=TOP2B_MOUSE&px=1&section=5&ave_thresh=1&_ignoreionsscorebelow=20&report=0&_sigthreshold=0.05&_msresflags=1089&_msresflags2=2&percolate=-1&percolate_rt=0))  **814 - 824 651.3492 1300.6839 1300.6849 -1 0 R.YIFTMLSSLAR.L**  ([Ions score 56](http://10.139.25.109/mascot/cgi/peptide_view.pl?file=../data/20120413/F007791.dat&query=16652&hit=1&index=TOP2B_MOUSE&px=1&section=5&ave_thresh=1&_ignoreionsscorebelow=20&report=0&_sigthreshold=0.05&_msresflags=1089&_msresflags2=2&percolate=-1&percolate_rt=0))  **1081 - 1089 558.8068 1115.5990 1115.6009 -2 0 K.DLIQMLVQR.G**  ([Ions score 26](http://10.139.25.109/mascot/cgi/peptide_view.pl?file=../data/20120413/F007791.dat&query=8765&hit=1&index=TOP2B_MOUSE&px=1&section=5&ave_thresh=1&_ignoreionsscorebelow=20&report=0&_sigthreshold=0.05&_msresflags=1089&_msresflags2=2&percolate=-1&percolate_rt=0))  **1428 - 1443 926.4304 1850.8462 1850.8475 -1 0 K.SQDFGNLFSFPSYSQK.S**  ([Ions score 89](http://10.139.25.109/mascot/cgi/peptide_view.pl?file=../data/20120413/F007791.dat&query=35284&hit=1&index=TOP2B_MOUSE&px=1&section=5&ave_thresh=1&_ignoreionsscorebelow=20&report=0&_sigthreshold=0.05&_msresflags=1089&_msresflags2=2&percolate=-1&percolate_rt=0))  13. [FLII_MOUSE](http://10.139.25.109/mascot/cgi/protein_view.pl?file=../data/20120413/F007792.dat&hit=FLII_MOUSE&db_idx=1&px=1&ave_thresh=1&_ignoreionsscorebelow=20&report=0&_sigthreshold=0.05&_msresflags=1089&_msresflags2=2&percolate=-1&percolate_rt=0)    **Mass:** 146137   **Score:** 247    **Matches:** 5(5)  **Sequences:** 5(5)  Protein flightless-1 homolog  Sequence Coverage: **5%**; Matched peptides shown in **Bold Red**  **1** MEATGVLPFV RGVDLSGNDF KGGYFPENVK AMTSLRWLKL NRTGLCYLPE ELAALQKLEH LSVSHNHLTT LHGELSSLPS LRAIVARANS LKNSGVPDDI  **101** FKLDDLSVLD LSHNQLTECP RELENAKNML VLNLSHNGID SIPNQLFINL TDLLYLDLSE NRLESLPPQM RRLVHLQTLV LNGNPLLHAQ LRQLPAMMAL  **201** QTLHLRNTQR TQSNLPTSLE GLSNLSDVDL SCNDLTRVPE CLYTLPSLRR **LNLSSNQIAE LSLCIDQWVH LETLNLSR**NQ LTSLPSAICK LTKLKKLYLN  **301** SNKLDFDGLP SGIGKLTSLE EFMAANNNLE LIPESLCRCP KLKKLVLNKN R**LVTLPEAIH FLTEIQVLDV R**ENPSLVMPP KPADRTAEWY NIDFSLQNQL  **401** RLAGASPATV AAAAAVGSGS KDPLARKMRL RRRKDSAQDV QAKQVLKGMS DVAQEKNKNQ EESIDARAPG GKVR**RWDQGL EKPR**LDYSEF FTEDVGQLPG  **501** LTIWQIENFV PVLVEEAFHG KFYEADCYIV LKTFLDDSGS LNWEIYYWIG GEATLDKKAC SAIHAVNLRN YLGAECRTVR EEMGDESEEF LQVFDNDISY  **601** IEGGTASGFY TVEDTHYVTR MYRVYGKKNI KLEPVPLKGS SLDPRFVFLL DQGLDIYVWR GAQATLSNTT KAR**LFAEKIN K**NERKGKAEI TLLVQGQEPP  **701** GFWDVLGGEP SEIKNHVPDD FWPPQPKLYK VGLGLGYLEL PQINYKLSVE HKKRPKVELM PGMR**LLQSLL DTR**CVYILDC WSDVFIWLGR KSPRLVRAAA  **801** LKLGQELCGM LHRPRHTVVS RSLEGTEAQV FKAKFKNWDD VLTVDYTRNA EAVLQGQGLS GKVKRDTEKT DQMKADLTAL FLPRQPPMPL AEAEQLMEEW  **901** NEDLDGMEGF VLEGRKFTRL PEEEFGHFYT QDCYVFLCRY WVPVEYEEEE KTEDKEGKAS AEAREGEEAA AEAEEKQPEE DFQCIVYFWQ GREASNMGWL  **1001** TFTFSLQKKF ESLFPGKLEV VRMTQQQENP KFLSHFKRKF IIHRGKRKVT QGTLQPTLYQ IRTNGSALCT RCIQINTDSS LLNSEFCFIL KVPFESEDNQ  **1101** GIVYAWVGRA SDPDEAKLAE DILNTMFDAS YSKQVINEGE EPENFFWVGI GAQKPYDDDA EYMKHTRLFR CSNEKGYFAV TEKCSDFCQD DLADDDIMLL  **1201** DNGQEVYMWV GTQTSQVEIK LSLKACQVYI QHTRSKEHER PRRLRLVRKG NEQRAFTRCF HAWSTFRQAP A    **Start - End Observed Mr(expt) Mr(calc) ppm Miss Sequence**  **251 - 278 1089.5675 3265.6791 3265.6765 1 0 R.LNLSSNQIAELSLCIDQWVHLETLNLSR.N** ([Ions score 71](http://10.139.25.109/mascot/cgi/peptide_view.pl?file=../data/20120413/F007792.dat&query=43916&hit=1&index=FLII_MOUSE&px=1&section=5&ave_thresh=1&_ignoreionsscorebelow=20&report=0&_sigthreshold=0.05&_msresflags=1089&_msresflags2=2&percolate=-1&percolate_rt=0))  **352 - 371 769.4421 2305.3029 2305.3046 1 0 R.LVTLPEAIHFLTEIQVLDVR.E** ([Ions score 63](http://10.139.25.109/mascot/cgi/peptide_view.pl?file=../data/20120413/F007792.dat&query=36038&hit=1&index=FLII_MOUSE&px=1&section=5&ave_thresh=1&_ignoreionsscorebelow=20&report=0&_sigthreshold=0.05&_msresflags=1089&_msresflags2=2&percolate=-1&percolate_rt=0))  **475 - 484 428.8992 1283.6757 1283.6734 2 1 R.RWDQGLEKPR.L**  ([Ions score 42](http://10.139.25.109/mascot/cgi/peptide_view.pl?file=../data/20120413/F007792.dat&query=12704&hit=1&index=FLII_MOUSE&px=1&section=5&ave_thresh=1&_ignoreionsscorebelow=20&report=0&_sigthreshold=0.05&_msresflags=1089&_msresflags2=2&percolate=-1&percolate_rt=0))  **674 - 681 481.7869 961.5593 961.5596 0 1 R.LFAEKINK.N**  ([Ions score 21](http://10.139.25.109/mascot/cgi/peptide_view.pl?file=../data/20120413/F007792.dat&query=3170&hit=1&index=FLII_MOUSE&px=1&section=5&ave_thresh=1&_ignoreionsscorebelow=20&report=0&_sigthreshold=0.05&_msresflags=1089&_msresflags2=2&percolate=-1&percolate_rt=0))  **765 - 773 529.8156 1057.6165 1057.6131 3 0 R.LLQSLLDTR.C**  ([Ions score 50](http://10.139.25.109/mascot/cgi/peptide_view.pl?file=../data/20120413/F007792.dat&query=5371&hit=1&index=FLII_MOUSE&px=1&section=5&ave_thresh=1&_ignoreionsscorebelow=20&report=0&_sigthreshold=0.05&_msresflags=1089&_msresflags2=2&percolate=-1&percolate_rt=0))  14. [CO6A2_MOUSE](http://10.139.25.109/mascot/cgi/protein_view.pl?file=../data/20120413/F007793.dat&hit=CO6A2_MOUSE&db_idx=1&px=1&ave_thresh=1&_ignoreionsscorebelow=20&report=0&_sigthreshold=0.05&_msresflags=1089&_msresflags2=2&percolate=-1&percolate_rt=0)    **Mass:** 111406   **Score:** 573    **Matches:** 12(12)  **Sequences:** 11(11)  Collagen alpha-2(VI) chain  Sequence Coverage: **13%;** Matched peptides shown in **Bold Red**  **1** MTTIKMLQGP LSVLLIGGLL GVLHAQQQEA ISPQEQEAVS PDISTTERNN NCPEKADCPV NVYFVLDTSE SVAMQSPTDS LLYHMQQFVP QFISQLQNEF  **101** YLDQVALSWR YGGLHFSDQV EVFSPPGSDR ASFTKSLQGI RSFRRGTFTD CALANMTQQI RQHVGKGVVN FAVVITDGHV TGSPCGGIKM QAERAREEGI  **201** R**LFAVAPNR**N LNEQGLR**DIA NSPHELYR**NN YATMRPDSTE IDQDTINRII KVMKHEAYGE CYKVSCLEIP GPHGPKGYR**G QKGAKGNMGE PGEPGQK**GRQ  **301** GDPGIEGPIG FPGPKGVPGF KGEKGEFGSD GRKGAPGLAG KNGTDGQKGK LGRIGPPGCK GDPGSRGPDG YPGEAGSPGE RGDQGAKGDS GRPGRRGPPG  **401** DPGDKGSKGY QGNNGAPGSP GVKGGKGGPG PRGPKGEPGR RGDPGTKGGP GSDGPKGEKG DPGPEGPRGL AGEVGSKGAK GDRGLPGPR**G PQGALGEPGK**  **501** QGSRGDPGDA GPRGDSGQPG PKGDPGRPGF SYPGPRGTPG EK**GEPGPPGP EGGR**GDFGLK GTPGRKGDKG EPADPGPPGE PGPRGPRGIP GPEGEPGPPG  **601** DPGLTECDVM TYVRETCGCC DCEKRCGALD VVFVIDSSES IGYTNFTLEK **NFVINVVNR**L GAIAKDPKSE TGTR**VGVVQY SHEGTFEAIR** LDDERVNSLS  **701** SFKEAVKNLE WIAGGTWTPS ALK**FAYNQLI K**ESRRQKTRV FAVVITDGRH DPRDDDLNLR ALCDRDVTVT AIGIGDMFHE THESENLYSI ACDKPQQVRN  **801** MTLFSDLVAE KFIDDMEDVL CPDPQIVCPE LPCQTELYVA QCTQRPVDIV FLLDGSER**LG EQNFHK**VRRF VEDVSRRLTL ARRDDDPLNA RMALLQYGSQ  **901** NQQQVAFPLT YNVTTIHEAL ER**ATYLNSFS HVGTGIVHAI NNVVR**GARGG ARRHAELSFV FLTDGVTGND SLEESVHSMR KQNVVPTVVA VGGDVDMDVL  **1001** TKISLGDRAA IFR**EKDFDSL AQPSFFDR**FI RWIC  **Start - End Observed Mr(expt) Mr(calc) ppm Miss Sequence**  **202 - 209 444.2580 886.5015 886.5025 -1 0 R.LFAVAPNR.N**  ([Ions score 47](http://10.139.25.109/mascot/cgi/peptide_view.pl?file=../data/20120413/F007793.dat&query=4658&hit=1&index=CO6A2_MOUSE&px=1&section=5&ave_thresh=1&_ignoreionsscorebelow=20&report=0&_sigthreshold=0.05&_msresflags=1089&_msresflags2=2&percolate=-1&percolate_rt=0))  **218 - 228 657.8249 1313.6353 1313.6364 -1 0 R.DIANSPHELYR.N**  ([Ions score 62](http://10.139.25.109/mascot/cgi/peptide_view.pl?file=../data/20120413/F007793.dat&query=23449&hit=1&index=CO6A2_MOUSE&px=1&section=5&ave_thresh=1&_ignoreionsscorebelow=20&report=0&_sigthreshold=0.05&_msresflags=1089&_msresflags2=2&percolate=-1&percolate_rt=0))  **280 - 297 443.2228 1768.8623 1768.8526 5 2 R.GQKGAKGNMGEPGEPGQK.G**  ([Ions score 20](http://10.139.25.109/mascot/cgi/peptide_view.pl?file=../data/20120413/F007793.dat&query=35227&hit=1&index=CO6A2_MOUSE&px=1&section=5&ave_thresh=1&_ignoreionsscorebelow=20&report=0&_sigthreshold=0.05&_msresflags=1089&_msresflags2=2&percolate=-1&percolate_rt=0))  **490 - 500 505.7668 1009.5191 1009.5193 -0 0 R.GPQGALGEPGK.Q**  ([Ions score 71](http://10.139.25.109/mascot/cgi/peptide_view.pl?file=../data/20120413/F007793.dat&query=9466&hit=1&index=CO6A2_MOUSE&px=1&section=5&ave_thresh=1&_ignoreionsscorebelow=20&report=0&_sigthreshold=0.05&_msresflags=1089&_msresflags2=2&percolate=-1&percolate_rt=0))  **543 - 554 553.7598 1105.5051 1105.5152 -9 0 K.GEPGPPGPEGGR.G**  ([Ions score 22](http://10.139.25.109/mascot/cgi/peptide_view.pl?file=../data/20120413/F007793.dat&query=13614&hit=1&index=CO6A2_MOUSE&px=1&section=5&ave_thresh=1&_ignoreionsscorebelow=20&report=0&_sigthreshold=0.05&_msresflags=1089&_msresflags2=2&percolate=-1&percolate_rt=0))  **651 - 659 537.8070 1073.5993 1073.5982 1 0 K.NFVINVVNR.L**  ([Ions score 50](http://10.139.25.109/mascot/cgi/peptide_view.pl?file=../data/20120413/F007793.dat&query=12233&hit=1&index=CO6A2_MOUSE&px=1&section=5&ave_thresh=1&_ignoreionsscorebelow=20&report=0&_sigthreshold=0.05&_msresflags=1089&_msresflags2=2&percolate=-1&percolate_rt=0))  **675 - 690 597.9730 1790.8956 1790.8951 0 0 R.VGVVQYSHEGTFEAIR.L**  [Ions score 46](http://10.139.25.109/mascot/cgi/peptide_view.pl?file=../data/20120413/F007793.dat&query=35893&hit=1&index=CO6A2_MOUSE&px=1&section=5&ave_thresh=1&_ignoreionsscorebelow=20&report=0&_sigthreshold=0.05&_msresflags=1089&_msresflags2=2&percolate=-1&percolate_rt=0))  **724 - 731 498.7793 995.5440 995.5440 0 0 K.FAYNQLIK.E**  ([Ions score 35](http://10.139.25.109/mascot/cgi/peptide_view.pl?file=../data/20120413/F007793.dat&query=8855&hit=1&index=CO6A2_MOUSE&px=1&section=5&ave_thresh=1&_ignoreionsscorebelow=20&report=0&_sigthreshold=0.05&_msresflags=1089&_msresflags2=2&percolate=-1&percolate_rt=0))  **859 - 866 486.7488 971.4830 971.4825 1 0 R.LGEQNFHK.V**  ([Ions score 35](http://10.139.25.109/mascot/cgi/peptide_view.pl?file=../data/20120413/F007793.dat&query=7867&hit=1&index=CO6A2_MOUSE&px=1&section=5&ave_thresh=1&_ignoreionsscorebelow=20&report=0&_sigthreshold=0.05&_msresflags=1089&_msresflags2=2&percolate=-1&percolate_rt=0))  **923 - 945 618.0808 2468.2919 2468.2924 0 0 R.ATYLNSFSHVGTGIVHAINNVVR.G** ([Ions score 32](http://10.139.25.109/mascot/cgi/peptide_view.pl?file=../data/20120413/F007793.dat&query=44364&hit=1&index=CO6A2_MOUSE&px=1&section=5&ave_thresh=1&_ignoreionsscorebelow=20&report=0&_sigthreshold=0.05&_msresflags=1089&_msresflags2=2&percolate=-1&percolate_rt=0))  **923 - 945 823.7721 2469.2929 2468.2924 0 0 R.ATYLNSFSHVGTGIVHAINNVVR.G** ([Ions score 98](http://10.139.25.109/mascot/cgi/peptide_view.pl?file=../data/20120413/F007793.dat&query=44365&hit=1&index=CO6A2_MOUSE&px=1&section=5&ave_thresh=1&_ignoreionsscorebelow=20&report=0&_sigthreshold=0.05&_msresflags=1089&_msresflags2=2&percolate=-1&percolate_rt=0))  **1014 - 1028 901.4235 1801.8314 1800.8319 0 1 R.EKDFDSLAQPSFFDR.F** ([Ions score 55](http://10.139.25.109/mascot/cgi/peptide_view.pl?file=../data/20120413/F007793.dat&query=36167&hit=1&index=CO6A2_MOUSE&px=1&section=5&ave_thresh=1&_ignoreionsscorebelow=20&report=0&_sigthreshold=0.05&_msresflags=1089&_msresflags2=2&percolate=-1&percolate_rt=0))  15. [YAP1_MOUSE](http://10.139.25.109/mascot/cgi/protein_view.pl?file=../data/20120413/F007792.dat&hit=YAP1_MOUSE&db_idx=1&px=1&ave_thresh=1&_ignoreionsscorebelow=20&report=0&_sigthreshold=0.05&_msresflags=1089&_msresflags2=2&percolate=-1&percolate_rt=0)    **Mass:** 52408    **Score:** 237    **Matches:** 6(6)  **Sequences:** 6(6)  Yorkie homolog  Sequence Coverage: **24%**; Matched peptides shown in **Bold Red**  **1** MEPAQQPPPQ PAPQGPAPPS VSPAGTPAAP PAPPAGHQVV HVRGDSETDL EALFNAVMNP KTANVPQTVP MRLRKLPDSF FKPPEPKSHS R**QASTDAGTA**  **101 GALTPQHVRA HSSPASLQLG AVSPGTLTAS GVVSGPAAAP AAQHLR**QSSF EIPDDVPLPA GWEMAKTSSG QR**YFLNHNDQ TTTWQDPR**KA MLSQLNVPAP  **201** ASPAVPQTLM NSASGPLPDG WEQAMTQDGE VYYINHKNKT TSWLDPRLDP RFAMNQRITQ SAPVKQPPPL APQSPQGGVL GGGSSNQQQQ IQLQQLQMEK  **301** ERLRLKQQEL FRQAIRNINP STANAPKCQE LALR**SQLPTL EQDGGTPNAV SSPGMSQELR** TMTTNSSDPF LNSGTYHSR**D ESTDSGLSMS SYSIPR**TPDD  **401** FLNSVDEMDT GDTISQSTLP SQQSRFPDYL EALPGTNVDL GTLEGDAMNI EGEELMPSLQ EALSSEILDV ESVLAATK**LD KESFLTWL**  **Start - End Observed Mr(expt) Mr(calc) ppm Miss Sequence**  **92 - 109 882.4404 1762.8663 1762.8598 4 0 R.QASTDAGTAGALTPQHVR.A**  Gln->pyro-Glu ([Ions score 94](http://10.139.25.109/mascot/cgi/peptide_view.pl?file=../data/20120413/F007792.dat&query=24676&hit=1&index=YAP1_MOUSE&px=1&section=5&ave_thresh=1&_ignoreionsscorebelow=20&report=0&_sigthreshold=0.05&_msresflags=1089&_msresflags2=2&percolate=-1&percolate_rt=0))  **110 - 146 859.4594 3433.8063 3433.8066 0 0 R.AHSSPASLQLGAVSPGTLTASGVVSGPAAAPAAQHLR.Q** ([Ions score 96](http://10.139.25.109/mascot/cgi/peptide_view.pl?file=../data/20120413/F007792.dat&query=44847&hit=1&index=YAP1_MOUSE&px=1&section=5&ave_thresh=1&_ignoreionsscorebelow=20&report=0&_sigthreshold=0.05&_msresflags=1089&_msresflags2=2&percolate=-1&percolate_rt=0))  **173 - 188 679.3143 2034.9195 2034.9184 1 0 R.YFLNHNDQTTTWQDPR.K**  ([Ions score 22](http://10.139.25.109/mascot/cgi/peptide_view.pl?file=../data/20120413/F007792.dat&query=32056&hit=1&index=YAP1_MOUSE&px=1&section=5&ave_thresh=1&_ignoreionsscorebelow=20&report=0&_sigthreshold=0.05&_msresflags=1089&_msresflags2=2&percolate=-1&percolate_rt=0))  **335 - 360 900.4373 2698.2885 2698.2868 1 0 R.SQLPTLEQDGGTPNAVSSPGMSQELR.T**  ([Ions score 64](http://10.139.25.109/mascot/cgi/peptide_view.pl?file=../data/20120413/F007792.dat&query=39952&hit=1&index=YAP1_MOUSE&px=1&section=5&ave_thresh=1&_ignoreionsscorebelow=20&report=0&_sigthreshold=0.05&_msresflags=1089&_msresflags2=2&percolate=-1&percolate_rt=0))  **380 - 396 916.4053 1830.7960 1830.7942 1 0 R.DESTDSGLSMSSYSIPR.T** ([Ions score 74](http://10.139.25.109/mascot/cgi/peptide_view.pl?file=../data/20120413/F007791.dat&query=34629&hit=1&index=YAP1_MOUSE&px=1&section=5&ave_thresh=1&_ignoreionsscorebelow=20&report=0&_sigthreshold=0.05&_msresflags=1089&_msresflags2=2&percolate=-1&percolate_rt=0))  **479 - 488 626.3349 1250.6552 1250.6547 0 1 K.LDKESFLTWL.-**  ([Ions score 43](http://10.139.25.109/mascot/cgi/peptide_view.pl?file=../data/20120413/F007791.dat&query=14485&hit=1&index=YAP1_MOUSE&px=1&section=5&ave_thresh=1&_ignoreionsscorebelow=20&report=0&_sigthreshold=0.05&_msresflags=1089&_msresflags2=2&percolate=-1&percolate_rt=0))  16. [HYEP_MOUSE](http://10.139.25.109/mascot/cgi/protein_view.pl?file=../data/20120413/F007793.dat&hit=HYEP_MOUSE&db_idx=1&px=1&ave_thresh=1&_ignoreionsscorebelow=20&report=0&_sigthreshold=0.05&_msresflags=1089&_msresflags2=2&percolate=-1&percolate_rt=0)    **Mass:** 52714    **Score:** 2047   **Matches:** 45(45)  **Sequences:** 28(28)  Epoxide hydrolase 1  Sequence Coverage: **56%**; Matched peptides shown in **Bold Red**  **1** MWLELILASV LGFVIYWFVS RDKEETLPLE DGWWGPGSKP SAK**EDESIRP FKVETSDEEI KDLHQR**IDRF RASPPLEGSR **FHYGFNSSYL KKVVSFWR**NE  **101** FDWRK**QVEIL NQYPHFKTKI EGLDIHFIHV KPPQLPSGR**T PKPLLMVHGW PGSFYEFYK**I IPLLTDPK**TH GLSDEHVFEV ICPSIPGYGF SEASSKK**GLN**  **201 SVATAR**IFYK LMSRLGFQKF YIQGGDWGSL ICTNIAQMVP NHVK**GLHLNM SFISRNIYSL TPLLGQR**FGR FLGYTEK**DLE LLYPFKEKVF YNIMRESGYL**  **301 HIQATKPDTV GCALNDSPVG LAAYILEKFS TWTKSEYREL EDGGLER**KFS LEDLLTNIMI YWTTGTIVSS QR**FYKENLGQ GVMVHR**HEGM K**VFVPTGYSA**  **401 FPSEILHAPE K**WVKVKYPK**L ISYSYMERGG HFAAFEEPKL LAQDIRKFVS LAELQ**  **Start - End Observed Mr(expt) Mr(calc) ppm Miss Sequence**  **44 - 52 560.7847 1119.5548 1119.5560 -1 0 K.EDESIRPFK.V**  ([Ions score 30](http://10.139.25.109/mascot/cgi/peptide_view.pl?file=../data/20120413/F007793.dat&query=14321&hit=1&index=HYEP_MOUSE&px=1&section=5&ave_thresh=1&_ignoreionsscorebelow=20&report=0&_sigthreshold=0.05&_msresflags=1089&_msresflags2=2&percolate=-1&percolate_rt=0))  **44 - 66 700.8491 2799.3651 2799.3675 1 2 K.EDESIRPFKVETSDEEIKDLHQR.I**  ([Ions score 52](http://10.139.25.109/mascot/cgi/peptide_view.pl?file=../data/20120413/F007793.dat&query=46406&hit=1&index=HYEP_MOUSE&px=1&section=5&ave_thresh=1&_ignoreionsscorebelow=20&report=0&_sigthreshold=0.05&_msresflags=1089&_msresflags2=2&percolate=-1&percolate_rt=0))  **44 - 66 934.1299 2800.3663 2799.3675 0 2 K.EDESIRPFKVETSDEEIKDLHQR.I**  ([Ions score 64](http://10.139.25.109/mascot/cgi/peptide_view.pl?file=../data/20120413/F007793.dat&query=46407&hit=1&index=HYEP_MOUSE&px=1&section=5&ave_thresh=1&_ignoreionsscorebelow=20&report=0&_sigthreshold=0.05&_msresflags=1089&_msresflags2=2&percolate=-1&percolate_rt=0))  **53 - 66 849.9139 1697.8132 1697.8220 -5 1 K.VETSDEEIKDLHQR.I**  ([Ions score 101](http://10.139.25.109/mascot/cgi/peptide_view.pl?file=../data/20120413/F007793.dat&query=33461&hit=1&index=HYEP_MOUSE&px=1&section=5&ave_thresh=1&_ignoreionsscorebelow=20&report=0&_sigthreshold=0.05&_msresflags=1089&_msresflags2=2&percolate=-1&percolate_rt=0))  **53 - 66 566.9471 1697.8196 1697.8220 -1 1 K.VETSDEEIKDLHQR.I**  ([Ions score 61](http://10.139.25.109/mascot/cgi/peptide_view.pl?file=../data/20120413/F007793.dat&query=33462&hit=1&index=HYEP_MOUSE&px=1&section=5&ave_thresh=1&_ignoreionsscorebelow=20&report=0&_sigthreshold=0.05&_msresflags=1089&_msresflags2=2&percolate=-1&percolate_rt=0))  **81 - 91 681.8273 1361.6400 1361.6404 0 0 R.FHYGFNSSYLK.K**  ([Ions score 67](http://10.139.25.109/mascot/cgi/peptide_view.pl?file=../data/20120413/F007793.dat&query=24824&hit=1&index=HYEP_MOUSE&px=1&section=5&ave_thresh=1&_ignoreionsscorebelow=20&report=0&_sigthreshold=0.05&_msresflags=1089&_msresflags2=2&percolate=-1&percolate_rt=0))  **81 - 91 454.8879 1361.6417 1361.6404 1 0 R.FHYGFNSSYLK.K**  ([Ions score 24](http://10.139.25.109/mascot/cgi/peptide_view.pl?file=../data/20120413/F007793.dat&query=24825&hit=1&index=HYEP_MOUSE&px=1&section=5&ave_thresh=1&_ignoreionsscorebelow=20&report=0&_sigthreshold=0.05&_msresflags=1089&_msresflags2=2&percolate=-1&percolate_rt=0))  **92 - 98 461.2690 920.5235 920.5232 0 1 K.KVVSFWR.N**  ([Ions score 32](http://10.139.25.109/mascot/cgi/peptide_view.pl?file=../data/20120413/F007793.dat&query=5924&hit=1&index=HYEP_MOUSE&px=1&section=5&ave_thresh=1&_ignoreionsscorebelow=20&report=0&_sigthreshold=0.05&_msresflags=1089&_msresflags2=2&percolate=-1&percolate_rt=0))  **93 - 98 397.2217 792.4287 792.4283 1 0 K.VVSFWR.N**  ([Ions score 29](http://10.139.25.109/mascot/cgi/peptide_view.pl?file=../data/20120413/F007793.dat&query=1921&hit=1&index=HYEP_MOUSE&px=1&section=5&ave_thresh=1&_ignoreionsscorebelow=20&report=0&_sigthreshold=0.05&_msresflags=1089&_msresflags2=2&percolate=-1&percolate_rt=0))  **106 - 117 505.9366 1514.7879 1514.7881 0 0 K.QVEILNQYPHFK.T**  ([Ions score 27](http://10.139.25.109/mascot/cgi/peptide_view.pl?file=../data/20120413/F007793.dat&query=28306&hit=1&index=HYEP_MOUSE&px=1&section=5&ave_thresh=1&_ignoreionsscorebelow=20&report=0&_sigthreshold=0.05&_msresflags=1089&_msresflags2=2&percolate=-1&percolate_rt=0))  **106 - 117 758.4022 1514.7898 1514.7881 1 0 K.QVEILNQYPHFK.T**  ([Ions score 66](http://10.139.25.109/mascot/cgi/peptide_view.pl?file=../data/20120413/F007793.dat&query=28309&hit=1&index=HYEP_MOUSE&px=1&section=5&ave_thresh=1&_ignoreionsscorebelow=20&report=0&_sigthreshold=0.05&_msresflags=1089&_msresflags2=2&percolate=-1&percolate_rt=0))  **118 - 139 828.1347 2481.3807 2481.3856 2 1 K.TKIEGLDIHFIHVKPPQLPSGR.T** ([Ions score 93](http://10.139.25.109/mascot/cgi/peptide_view.pl?file=../data/20120413/F007793.dat&query=44460&hit=1&index=HYEP_MOUSE&px=1&section=5&ave_thresh=1&_ignoreionsscorebelow=20&report=0&_sigthreshold=0.05&_msresflags=1089&_msresflags2=2&percolate=-1&percolate_rt=0))  **118 - 139 621.3516 2481.3751 2481.3856 4 1 K.TKIEGLDIHFIHVKPPQLPSGR.T**  ([Ions score 49](http://10.139.25.109/mascot/cgi/peptide_view.pl?file=../data/20120413/F007793.dat&query=44462&hit=1&index=HYEP_MOUSE&px=1&section=5&ave_thresh=1&_ignoreionsscorebelow=20&report=0&_sigthreshold=0.05&_msresflags=1089&_msresflags2=2&percolate=-1&percolate_rt=0))  **120 - 139 564.0685 2252.2427 2252.2430 0 0 K.IEGLDIHFIHVKPPQLPSGR.T** ([Ions score 39](http://10.139.25.109/mascot/cgi/peptide_view.pl?file=../data/20120413/F007793.dat&query=42986&hit=1&index=HYEP_MOUSE&px=1&section=5&ave_thresh=1&_ignoreionsscorebelow=20&report=0&_sigthreshold=0.05&_msresflags=1089&_msresflags2=2&percolate=-1&percolate_rt=0))  **160 - 168 505.3181 1008.6216 1008.6219 0 0 K.IIPLLTDPK.T**  ([Ions score 39](http://10.139.25.109/mascot/cgi/peptide_view.pl?file=../data/20120413/F007793.dat&query=9442&hit=1&index=HYEP_MOUSE&px=1&section=5&ave_thresh=1&_ignoreionsscorebelow=20&report=0&_sigthreshold=0.05&_msresflags=1089&_msresflags2=2&percolate=-1&percolate_rt=0))  **198 - 206 444.7483 887.4820 887.4825 -1 0 K.GLNSVATAR.I**  ([Ions score 52](http://10.139.25.109/mascot/cgi/peptide_view.pl?file=../data/20120413/F007793.dat&query=4699&hit=1&index=HYEP_MOUSE&px=1&section=5&ave_thresh=1&_ignoreionsscorebelow=20&report=0&_sigthreshold=0.05&_msresflags=1089&_msresflags2=2&percolate=-1&percolate_rt=0))  **245 - 255 645.8339 1289.6532 1289.6550 -1 0 K.GLHLNMSFISR.N**  Oxidation (M) ([Ions score 62](http://10.139.25.109/mascot/cgi/peptide_view.pl?file=../data/20120413/F007793.dat&query=22550&hit=1&index=HYEP_MOUSE&px=1&section=5&ave_thresh=1&_ignoreionsscorebelow=20&report=0&_sigthreshold=0.05&_msresflags=1089&_msresflags2=2&percolate=-1&percolate_rt=0))  **256 - 267 687.8907 1373.7668 1373.7667 0 0 R.NIYSLTPLLGQR.F**  ([Ions score 57](http://10.139.25.109/mascot/cgi/peptide_view.pl?file=../data/20120413/F007793.dat&query=25073&hit=1&index=HYEP_MOUSE&px=1&section=5&ave_thresh=1&_ignoreionsscorebelow=20&report=0&_sigthreshold=0.05&_msresflags=1089&_msresflags2=2&percolate=-1&percolate_rt=0))  **256 - 267 458.9298 1373.7676 1373.7667 1 0 R.NIYSLTPLLGQR.F**  ([Ions score 32](http://10.139.25.109/mascot/cgi/peptide_view.pl?file=../data/20120413/F007793.dat&query=25075&hit=1&index=HYEP_MOUSE&px=1&section=5&ave_thresh=1&_ignoreionsscorebelow=20&report=0&_sigthreshold=0.05&_msresflags=1089&_msresflags2=2&percolate=-1&percolate_rt=0))  **256 - 267 687.8920 1373.7695 1373.7667 2 0 R.NIYSLTPLLGQR.F**  ([Ions score 53](http://10.139.25.109/mascot/cgi/peptide_view.pl?file=../data/20120413/F007793.dat&query=25078&hit=1&index=HYEP_MOUSE&px=1&section=5&ave_thresh=1&_ignoreionsscorebelow=20&report=0&_sigthreshold=0.05&_msresflags=1089&_msresflags2=2&percolate=-1&percolate_rt=0))  **278 - 286 569.3124 1136.6103 1136.6117 -1 0 K.DLELLYPFK.E**  ([Ions score 47](http://10.139.25.109/mascot/cgi/peptide_view.pl?file=../data/20120413/F007793.dat&query=15266&hit=1&index=HYEP_MOUSE&px=1&section=5&ave_thresh=1&_ignoreionsscorebelow=20&report=0&_sigthreshold=0.05&_msresflags=1089&_msresflags2=2&percolate=-1&percolate_rt=0))  **278 - 288 697.8807 1393.7468 1393.7493 -2 1 K.DLELLYPFKEK.V**  ([Ions score 50](http://10.139.25.109/mascot/cgi/peptide_view.pl?file=../data/20120413/F007793.dat&query=25587&hit=1&index=HYEP_MOUSE&px=1&section=5&ave_thresh=1&_ignoreionsscorebelow=20&report=0&_sigthreshold=0.05&_msresflags=1089&_msresflags2=2&percolate=-1&percolate_rt=0))  **278 - 288 465.5903 1393.7491 1393.7493 0 1 K.DLELLYPFKEK.V**  ([Ions score 21](http://10.139.25.109/mascot/cgi/peptide_view.pl?file=../data/20120413/F007793.dat&query=25588&hit=1&index=HYEP_MOUSE&px=1&section=5&ave_thresh=1&_ignoreionsscorebelow=20&report=0&_sigthreshold=0.05&_msresflags=1089&_msresflags2=2&percolate=-1&percolate_rt=0))  **289 - 295 471.7462 941.4777 941.4793 -2 0 K.VFYNIMR.E**  ([Ions score 51](http://10.139.25.109/mascot/cgi/peptide_view.pl?file=../data/20120413/F007793.dat&query=6714&hit=1&index=HYEP_MOUSE&px=1&section=5&ave_thresh=1&_ignoreionsscorebelow=20&report=0&_sigthreshold=0.05&_msresflags=1089&_msresflags2=2&percolate=-1&percolate_rt=0))  **289 - 295 479.7447 957.4748 957.4742 1 0 K.VFYNIMR.E**  Oxidation (M) ([Ions score 46](http://10.139.25.109/mascot/cgi/peptide_view.pl?file=../data/20120413/F007793.dat&query=7330&hit=1&index=HYEP_MOUSE&px=1&section=5&ave_thresh=1&_ignoreionsscorebelow=20&report=0&_sigthreshold=0.05&_msresflags=1089&_msresflags2=2&percolate=-1&percolate_rt=0))  **296 - 328 883.4518 3529.7759 3529.7763 0 0 R.ESGYLHIQATKPDTVGCALNDSPVGLAAYILEK.F** ([Ions score 28](http://10.139.25.109/mascot/cgi/peptide_view.pl?file=../data/20120413/F007793.dat&query=48298&hit=1&index=HYEP_MOUSE&px=1&section=5&ave_thresh=1&_ignoreionsscorebelow=20&report=0&_sigthreshold=0.05&_msresflags=1089&_msresflags2=2&percolate=-1&percolate_rt=0))  **329 - 334 385.1978 768.3811 768.3806 1 0 K.FSTWTK.S**  ([Ions score 20](http://10.139.25.109/mascot/cgi/peptide_view.pl?file=../data/20120413/F007793.dat&query=1339&hit=1&index=HYEP_MOUSE&px=1&section=5&ave_thresh=1&_ignoreionsscorebelow=20&report=0&_sigthreshold=0.05&_msresflags=1089&_msresflags2=2&percolate=-1&percolate_rt=0))  **335 - 347 518.2458 1551.7155 1551.7165 -1 1 K.SEYRELEDGGLER.K**  ([Ions score 46](http://10.139.25.109/mascot/cgi/peptide_view.pl?file=../data/20120413/F007793.dat&query=29377&hit=1&index=HYEP_MOUSE&px=1&section=5&ave_thresh=1&_ignoreionsscorebelow=20&report=0&_sigthreshold=0.05&_msresflags=1089&_msresflags2=2&percolate=-1&percolate_rt=0))  **339 - 347 509.2458 1016.4770 1016.4774 0 0 R.ELEDGGLER.K**  ([Ions score 40](http://10.139.25.109/mascot/cgi/peptide_view.pl?file=../data/20120413/F007793.dat&query=9714&hit=1&index=HYEP_MOUSE&px=1&section=5&ave_thresh=1&_ignoreionsscorebelow=20&report=0&_sigthreshold=0.05&_msresflags=1089&_msresflags2=2&percolate=-1&percolate_rt=0))  **373 - 386 839.4292 1676.8438 1676.8457 -1 1 R.FYKENLGQGVMVHR.H**  ([Ions score 50](http://10.139.25.109/mascot/cgi/peptide_view.pl?file=../data/20120413/F007793.dat&query=32982&hit=1&index=HYEP_MOUSE&px=1&section=5&ave_thresh=1&_ignoreionsscorebelow=20&report=0&_sigthreshold=0.05&_msresflags=1089&_msresflags2=2&percolate=-1&percolate_rt=0))  **373 - 386 559.9554 1676.8445 1676.8457 -1 1 R.FYKENLGQGVMVHR.H**  ([Ions score 39](http://10.139.25.109/mascot/cgi/peptide_view.pl?file=../data/20120413/F007793.dat&query=32983&hit=1&index=HYEP_MOUSE&px=1&section=5&ave_thresh=1&_ignoreionsscorebelow=20&report=0&_sigthreshold=0.05&_msresflags=1089&_msresflags2=2&percolate=-1&percolate_rt=0))  **373 - 386 424.2172 1692.8399 1692.8406 0 1 R.FYKENLGQGVMVHR.H**  Oxidation (M) ([Ions score 27](http://10.139.25.109/mascot/cgi/peptide_view.pl?file=../data/20120413/F007793.dat&query=33304&hit=1&index=HYEP_MOUSE&px=1&section=5&ave_thresh=1&_ignoreionsscorebelow=20&report=0&_sigthreshold=0.05&_msresflags=1089&_msresflags2=2&percolate=-1&percolate_rt=0))  **373 - 386 565.2873 1692.8400 1692.8406 0 1 R.FYKENLGQGVMVHR.H**  Oxidation (M) ([Ions score 37](http://10.139.25.109/mascot/cgi/peptide_view.pl?file=../data/20120413/F007793.dat&query=33305&hit=1&index=HYEP_MOUSE&px=1&section=5&ave_thresh=1&_ignoreionsscorebelow=20&report=0&_sigthreshold=0.05&_msresflags=1089&_msresflags2=2&percolate=-1&percolate_rt=0))  **373 - 386 847.4278 1693.8400 1692.8406 0 1 R.FYKENLGQGVMVHR.H**  Oxidation (M) ([Ions score 50](http://10.139.25.109/mascot/cgi/peptide_view.pl?file=../data/20120413/F007793.dat&query=33332&hit=1&index=HYEP_MOUSE&px=1&section=5&ave_thresh=1&_ignoreionsscorebelow=20&report=0&_sigthreshold=0.05&_msresflags=1089&_msresflags2=2&percolate=-1&percolate_rt=0))  **392 - 411 730.3803 2188.1189 2188.1205 -1 0 K.VFVPTGYSAFPSEILHAPEK.W**  ([Ions score 24](http://10.139.25.109/mascot/cgi/peptide_view.pl?file=../data/20120413/F007793.dat&query=42470&hit=1&index=HYEP_MOUSE&px=1&section=5&ave_thresh=1&_ignoreionsscorebelow=20&report=0&_sigthreshold=0.05&_msresflags=1089&_msresflags2=2&percolate=-1&percolate_rt=0))  **420 - 428 581.2838 1160.5529 1160.5536 -1 0 K.LISYSYMER.G**  ([Ions score 68](http://10.139.25.109/mascot/cgi/peptide_view.pl?file=../data/20120413/F007793.dat&query=16399&hit=1&index=HYEP_MOUSE&px=1&section=5&ave_thresh=1&_ignoreionsscorebelow=20&report=0&_sigthreshold=0.05&_msresflags=1089&_msresflags2=2&percolate=-1&percolate_rt=0))  **420 - 428 589.2822 1176.5498 1176.5485 1 0 K.LISYSYMER.G**  Oxidation (M) ([Ions score 43](http://10.139.25.109/mascot/cgi/peptide_view.pl?file=../data/20120413/F007793.dat&query=17131&hit=1&index=HYEP_MOUSE&px=1&section=5&ave_thresh=1&_ignoreionsscorebelow=20&report=0&_sigthreshold=0.05&_msresflags=1089&_msresflags2=2&percolate=-1&percolate_rt=0))  **429 - 439 595.2847 1188.5549 1188.5564 -1 0 R.GGHFAAFEEPK.L**  ([Ions score 51](http://10.139.25.109/mascot/cgi/peptide_view.pl?file=../data/20120413/F007793.dat&query=17765&hit=1&index=HYEP_MOUSE&px=1&section=5&ave_thresh=1&_ignoreionsscorebelow=20&report=0&_sigthreshold=0.05&_msresflags=1089&_msresflags2=2&percolate=-1&percolate_rt=0))  **429 - 439 397.1927 1188.5563 1188.5564 0 0 R.GGHFAAFEEPK.L**  ([Ions score 31](http://10.139.25.109/mascot/cgi/peptide_view.pl?file=../data/20120413/F007793.dat&query=17769&hit=1&index=HYEP_MOUSE&px=1&section=5&ave_thresh=1&_ignoreionsscorebelow=20&report=0&_sigthreshold=0.05&_msresflags=1089&_msresflags2=2&percolate=-1&percolate_rt=0))  **429 - 439 595.2856 1188.5567 1188.5564 0 0 R.GGHFAAFEEPK.L**  ([Ions score 56](http://10.139.25.109/mascot/cgi/peptide_view.pl?file=../data/20120413/F007793.dat&query=17770&hit=1&index=HYEP_MOUSE&px=1&section=5&ave_thresh=1&_ignoreionsscorebelow=20&report=0&_sigthreshold=0.05&_msresflags=1089&_msresflags2=2&percolate=-1&percolate_rt=0))  **440 - 446 414.7501 827.4856 827.4865 -1 0 K.LLAQDIR.K**  ([Ions score 46](http://10.139.25.109/mascot/cgi/peptide_view.pl?file=../data/20120413/F007793.dat&query=2812&hit=1&index=HYEP_MOUSE&px=1&section=5&ave_thresh=1&_ignoreionsscorebelow=20&report=0&_sigthreshold=0.05&_msresflags=1089&_msresflags2=2&percolate=-1&percolate_rt=0))  **440 - 447 319.5341 955.5804 955.5814 -1 1 K.LLAQDIRK.F**  ([Ions score 20](http://10.139.25.109/mascot/cgi/peptide_view.pl?file=../data/20120413/F007793.dat&query=7266&hit=1&index=HYEP_MOUSE&px=1&section=5&ave_thresh=1&_ignoreionsscorebelow=20&report=0&_sigthreshold=0.05&_msresflags=1089&_msresflags2=2&percolate=-1&percolate_rt=0))  **440 - 447 478.7981 955.5816 955.5814 0 1 K.LLAQDIRK.F**  ([Ions score 50](http://10.139.25.109/mascot/cgi/peptide_view.pl?file=../data/20120413/F007793.dat&query=7268&hit=1&index=HYEP_MOUSE&px=1&section=5&ave_thresh=1&_ignoreionsscorebelow=20&report=0&_sigthreshold=0.05&_msresflags=1089&_msresflags2=2&percolate=-1&percolate_rt=0))  **447 - 455 517.7980 1033.5814 1033.5808 1 1 R.KFVSLAELQ.-**  ([Ions score 36](http://10.139.25.109/mascot/cgi/peptide_view.pl?file=../data/20120413/F007793.dat&query=10528&hit=1&index=HYEP_MOUSE&px=1&section=5&ave_thresh=1&_ignoreionsscorebelow=20&report=0&_sigthreshold=0.05&_msresflags=1089&_msresflags2=2&percolate=-1&percolate_rt=0))  **448 - 455 453.7505 905.4864 905.4858 1 0 K.FVSLAELQ.-**  ([Ions score 41](http://10.139.25.109/mascot/cgi/peptide_view.pl?file=../data/20120413/F007793.dat&query=5311&hit=1&index=HYEP_MOUSE&px=1&section=5&ave_thresh=1&_ignoreionsscorebelow=20&report=0&_sigthreshold=0.05&_msresflags=1089&_msresflags2=2&percolate=-1&percolate_rt=0))  17. [DYN1_MOUSE](http://10.139.25.109/mascot/cgi/protein_view.pl?file=../data/20120413/F007793.dat&hit=DYN1_MOUSE&db_idx=1&px=1&ave_thresh=1&_ignoreionsscorebelow=20&report=0&_sigthreshold=0.05&_msresflags=1089&_msresflags2=2&percolate=-1&percolate_rt=0)    **Mass:** 98140    **Score:** 1548   **Matches:** 31(31)  **Sequences:** 27(27)  Dynamin-1  Sequence Coverage: **36%**; Matched peptides shown in **Bold Red**  **1** MGNR**GMEDLI PLVNR**LQDAF SAIGQNADLD LPQIAVVGGQ SAGK**SSVLEN FVGRDFLPR**G SGIVTR**RPLV LQLVNSTTEY AEFLHCK**GKK FTDFEEVRLE  **101** IEAETDRVTG TNK**GISPVPI NLRVYSPHVL NLTLVDLPGM TKVPVGDQPP DIEFQIRDML MQFVTKENCL ILAVSPANSD LANSDALK**IA KEVDPQGQRT  **201** IGVITK**LDLM DEGTDARDVL ENKLLPLRRG YIGVVNR**SQK DIDGKKDITA ALAAERK**FFL SHPSYR**HLAD R**MGTPYLQKV LNQQLTNHIR** DTLPGLRNK**L**  **301 QSQLLSIEK**E VDEYKNFRPD DPARKTK**ALL QMVQQFAVDF EKR**IEGSGDQ IDTYELSGGA RINRIFHER**F PFELVK**MEFD EKELRREISY AIKNIHGIR**T**  **401 GLFTPDMAFE TIVK**KQVKKI REPCLKCVDM VISELISTVR QCTKKLQQYP RLREEMER**IV TTHIR**EREGR TKEQVMLLID IELAYMNTNH EDFIGFANAQ  **501** QRSNQMNKKK TSGNQDEILV IRKGWLTINN IGIMKGGSKE YWFVLTAENL SWYKDDEEKE KK**YMLSVDNL K**LRDVEKGFM SSK**HIFALFN TEQR**NVYKDY  **601** R**QLELACETQ EEVDSWK**ASF LRAGVYPERV GDKEKASETE ENGSDSFMHS MDPQLERQVE TIR**NLVDSYM AIVNK**TVRDL MPK**TIMHLMI NNTK**EFIFSE  **701** LLANLYSCGD QNTLMEESAE QAQRRDEMLR MYHALKEALS IIGDINTTTV STPMPPPVDD SWLQVQSVPA GRRSPTSSPT PQRRAPAVPP ARPGSRGPAP  **801** GPPPAGSALG GAPPVPSRPG ASPDPFGPPP QVPSRPNR**AP PGVPSLGAWR LNSPQGKHEN R**AGKARL  **Start - End Observed Mr(expt) Mr(calc) ppm Miss Sequence**  **5 - 15 636.8367 1271.6589 1271.6544 4 0 R.GMEDLIPLVNR.L**  Oxidation (M) ([Ions score 44](http://10.139.25.109/mascot/cgi/peptide_view.pl?file=../data/20120413/F007793.dat&query=21826&hit=1&index=DYN1_MOUSE&px=1&section=5&ave_thresh=1&_ignoreionsscorebelow=20&report=0&_sigthreshold=0.05&_msresflags=1089&_msresflags2=2&percolate=-1&percolate_rt=0))  **45 - 54 554.2929 1106.5713 1106.5720 -1 0 K.SSVLENFVGR.D**  ([Ions score 65](http://10.139.25.109/mascot/cgi/peptide_view.pl?file=../data/20120413/F007793.dat&query=13682&hit=1&index=DYN1_MOUSE&px=1&section=5&ave_thresh=1&_ignoreionsscorebelow=20&report=0&_sigthreshold=0.05&_msresflags=1089&_msresflags2=2&percolate=-1&percolate_rt=0))  **45 - 59 579.3088 1734.9045 1734.9053 0 1 K.SSVLENFVGRDFLPR.G**  ([Ions score 53](http://10.139.25.109/mascot/cgi/peptide_view.pl?file=../data/20120413/F007793.dat&query=34376&hit=1&index=DYN1_MOUSE&px=1&section=5&ave_thresh=1&_ignoreionsscorebelow=20&report=0&_sigthreshold=0.05&_msresflags=1089&_msresflags2=2&percolate=-1&percolate_rt=0))  **67 - 87 630.3335 2517.3027 2517.3050 1 0 R.RPLVLQLVNSTTEYAEFLHCK.G**  ([Ions score 40](http://10.139.25.109/mascot/cgi/peptide_view.pl?file=../data/20120413/F007793.dat&query=44734&hit=1&index=DYN1_MOUSE&px=1&section=5&ave_thresh=1&_ignoreionsscorebelow=20&report=0&_sigthreshold=0.05&_msresflags=1089&_msresflags2=2&percolate=-1&percolate_rt=0))  **67 - 87 840.1093 2517.3045 2517.3050 0 0 R.RPLVLQLVNSTTEYAEFLHCK.G**  ([Ions score 81](http://10.139.25.109/mascot/cgi/peptide_view.pl?file=../data/20120413/F007793.dat&query=44735&hit=1&index=DYN1_MOUSE&px=1&section=5&ave_thresh=1&_ignoreionsscorebelow=20&report=0&_sigthreshold=0.05&_msresflags=1089&_msresflags2=2&percolate=-1&percolate_rt=0))  **114 - 123 533.3251 1064.6357 1064.6342 1 0 K.GISPVPINLR.V**  ([Ions score 40](http://10.139.25.109/mascot/cgi/peptide_view.pl?file=../data/20120413/F007793.dat&query=11825&hit=1&index=DYN1_MOUSE&px=1&section=5&ave_thresh=1&_ignoreionsscorebelow=20&report=0&_sigthreshold=0.05&_msresflags=1089&_msresflags2=2&percolate=-1&percolate_rt=0))  **124 - 142 705.0512 2112.1318 2112.1289 1 0 R.VYSPHVLNLTLVDLPGMTK.V**  Oxidation (M) ([Ions score 44](http://10.139.25.109/mascot/cgi/peptide_view.pl?file=../data/20120413/F007793.dat&query=41800&hit=1&index=DYN1_MOUSE&px=1&section=5&ave_thresh=1&_ignoreionsscorebelow=20&report=0&_sigthreshold=0.05&_msresflags=1089&_msresflags2=2&percolate=-1&percolate_rt=0))  **124 - 142 1057.0717 2112.1278 2112.1289 1 0 R.VYSPHVLNLTLVDLPGMTK.V**  Oxidation (M) ([Ions score 47](http://10.139.25.109/mascot/cgi/peptide_view.pl?file=../data/20120413/F007793.dat&query=41808&hit=1&index=DYN1_MOUSE&px=1&section=5&ave_thresh=1&_ignoreionsscorebelow=20&report=0&_sigthreshold=0.05&_msresflags=1089&_msresflags2=2&percolate=-1&percolate_rt=0))  **143 - 157 855.4448 1708.8751 1708.8784 -2 0 K.VPVGDQPPDIEFQIR.D**  ([Ions score 63](http://10.139.25.109/mascot/cgi/peptide_view.pl?file=../data/20120413/F007793.dat&query=33726&hit=1&index=DYN1_MOUSE&px=1&section=5&ave_thresh=1&_ignoreionsscorebelow=20&report=0&_sigthreshold=0.05&_msresflags=1089&_msresflags2=2&percolate=-1&percolate_rt=0))  **158 - 166 572.7712 1143.5278 1143.5304 -2 0 R.DMLMQFVTK.E**  2 Oxidation (M) ([Ions score 46](http://10.139.25.109/mascot/cgi/peptide_view.pl?file=../data/20120413/F007793.dat&query=15627&hit=1&index=DYN1_MOUSE&px=1&section=5&ave_thresh=1&_ignoreionsscorebelow=20&report=0&_sigthreshold=0.05&_msresflags=1089&_msresflags2=2&percolate=-1&percolate_rt=0))  **167 - 188 1158.0821 2315.1486 2314.1475 0 0 K.ENCLILAVSPANSDLANSDALK.I** ([Ions score 67](http://10.139.25.109/mascot/cgi/peptide_view.pl?file=../data/20120413/F007793.dat&query=43394&hit=1&index=DYN1_MOUSE&px=1&section=5&ave_thresh=1&_ignoreionsscorebelow=20&report=0&_sigthreshold=0.05&_msresflags=1089&_msresflags2=2&percolate=-1&percolate_rt=0))  **207 - 223 650.6428 1948.9050 1948.8815 10 1 K.LDLMDEGTDARDVLENK.L**  Oxidation (M) ([Ions score 57](http://10.139.25.109/mascot/cgi/peptide_view.pl?file=../data/20120413/F007793.dat&query=39344&hit=1&index=DYN1_MOUSE&px=1&section=5&ave_thresh=1&_ignoreionsscorebelow=20&report=0&_sigthreshold=0.05&_msresflags=1089&_msresflags2=2&percolate=-1&percolate_rt=0))  **224 - 229 384.2662 766.5178 766.5177 0 1 K.LLPLRR.G**  ([Ions score 24](http://10.139.25.109/mascot/cgi/peptide_view.pl?file=../data/20120413/F007793.dat&query=1310&hit=1&index=DYN1_MOUSE&px=1&section=5&ave_thresh=1&_ignoreionsscorebelow=20&report=0&_sigthreshold=0.05&_msresflags=1089&_msresflags2=2&percolate=-1&percolate_rt=0))  **230 - 237 439.2482 876.4819 876.4818 0 0 R.GYIGVVNR.S**  ([Ions score 37](http://10.139.25.109/mascot/cgi/peptide_view.pl?file=../data/20120413/F007793.dat&query=4315&hit=1&index=DYN1_MOUSE&px=1&section=5&ave_thresh=1&_ignoreionsscorebelow=20&report=0&_sigthreshold=0.05&_msresflags=1089&_msresflags2=2&percolate=-1&percolate_rt=0))  **258 - 266 577.2939 1152.5732 1152.5716 1 0 K.FFLSHPSYR.H**  ([Ions score 37](http://10.139.25.109/mascot/cgi/peptide_view.pl?file=../data/20120413/F007793.dat&query=16072&hit=1&index=DYN1_MOUSE&px=1&section=5&ave_thresh=1&_ignoreionsscorebelow=20&report=0&_sigthreshold=0.05&_msresflags=1089&_msresflags2=2&percolate=-1&percolate_rt=0))  **272 - 279 477.2414 952.4683 952.4688 -1 0 R.MGTPYLQK.V**  Oxidation (M) ([Ions score 37](http://10.139.25.109/mascot/cgi/peptide_view.pl?file=../data/20120413/F007793.dat&query=7148&hit=1&index=DYN1_MOUSE&px=1&section=5&ave_thresh=1&_ignoreionsscorebelow=20&report=0&_sigthreshold=0.05&_msresflags=1089&_msresflags2=2&percolate=-1&percolate_rt=0))  **280 - 290 668.3780 1334.7414 1334.7419 0 0 K.VLNQQLTNHIR.D**  ([Ions score 71](http://10.139.25.109/mascot/cgi/peptide_view.pl?file=../data/20120413/F007793.dat&query=24163&hit=1&index=DYN1_MOUSE&px=1&section=5&ave_thresh=1&_ignoreionsscorebelow=20&report=0&_sigthreshold=0.05&_msresflags=1089&_msresflags2=2&percolate=-1&percolate_rt=0))  **280 - 290 445.9214 1334.7423 1334.7419 0 0 K.VLNQQLTNHIR.D**  ([Ions score 38](http://10.139.25.109/mascot/cgi/peptide_view.pl?file=../data/20120413/F007793.dat&query=24165&hit=1&index=DYN1_MOUSE&px=1&section=5&ave_thresh=1&_ignoreionsscorebelow=20&report=0&_sigthreshold=0.05&_msresflags=1089&_msresflags2=2&percolate=-1&percolate_rt=0))  **300 - 309 579.8413 1157.6681 1157.6656 2 0 K.LQSQLLSIEK.E**  ([Ions score 50](http://10.139.25.109/mascot/cgi/peptide_view.pl?file=../data/20120413/F007793.dat&query=16310&hit=1&index=DYN1_MOUSE&px=1&section=5&ave_thresh=1&_ignoreionsscorebelow=20&report=0&_sigthreshold=0.05&_msresflags=1089&_msresflags2=2&percolate=-1&percolate_rt=0))  **328 - 342 891.9565 1781.8974 1781.8789 9 0 K.ALLQMVQQFAVDFEK.R**  Oxidation (M) ([Ions score 70](http://10.139.25.109/mascot/cgi/peptide_view.pl?file=../data/20120413/F007793.dat&query=35581&hit=1&index=DYN1_MOUSE&px=1&section=5&ave_thresh=1&_ignoreionsscorebelow=20&report=0&_sigthreshold=0.05&_msresflags=1089&_msresflags2=2&percolate=-1&percolate_rt=0))  **328 - 343 647.0104 1938.0093 1938.0033 3 1 K.ALLQMVQQFAVDFEKR.I**  Oxidation (M) ([Ions score 69](http://10.139.25.109/mascot/cgi/peptide_view.pl?file=../data/20120413/F007793.dat&query=39107&hit=1&index=DYN1_MOUSE&px=1&section=5&ave_thresh=1&_ignoreionsscorebelow=20&report=0&_sigthreshold=0.05&_msresflags=1089&_msresflags2=2&percolate=-1&percolate_rt=0))  **370 - 376 440.2523 878.4901 878.4902 0 0 R.FPFELVK.M**  ([Ions score 24](http://10.139.25.109/mascot/cgi/peptide_view.pl?file=../data/20120413/F007793.dat&query=4370&hit=1&index=DYN1_MOUSE&px=1&section=5&ave_thresh=1&_ignoreionsscorebelow=20&report=0&_sigthreshold=0.05&_msresflags=1089&_msresflags2=2&percolate=-1&percolate_rt=0))  **400 - 414 843.4253 1684.8361 1684.8382 -1 0 R.TGLFTPDMAFETIVK.K**  Oxidation (M) ([Ions score 56](http://10.139.25.109/mascot/cgi/peptide_view.pl?file=../data/20120413/F007793.dat&query=33132&hit=1&index=DYN1_MOUSE&px=1&section=5&ave_thresh=1&_ignoreionsscorebelow=20&report=0&_sigthreshold=0.05&_msresflags=1089&_msresflags2=2&percolate=-1&percolate_rt=0))  **459 - 465 420.2585 838.5025 838.5025 0 0 R.IVTTHIR.E**  ([Ions score 26](http://10.139.25.109/mascot/cgi/peptide_view.pl?file=../data/20120413/F007793.dat&query=3148&hit=1&index=DYN1_MOUSE&px=1&section=5&ave_thresh=1&_ignoreionsscorebelow=20&report=0&_sigthreshold=0.05&_msresflags=1089&_msresflags2=2&percolate=-1&percolate_rt=0))  **563 - 571 549.7794 1097.5443 1097.5427 1 0 K.YMLSVDNLK.L**  Oxidation (M) ([Ions score 49](http://10.139.25.109/mascot/cgi/peptide_view.pl?file=../data/20120413/F007793.dat&query=13263&hit=1&index=DYN1_MOUSE&px=1&section=5&ave_thresh=1&_ignoreionsscorebelow=20&report=0&_sigthreshold=0.05&_msresflags=1089&_msresflags2=2&percolate=-1&percolate_rt=0))  **584 - 594 688.3578 1374.7011 1374.7044 -2 0 K.HIFALFNTEQR.N**  ([Ions score 76](http://10.139.25.109/mascot/cgi/peptide_view.pl?file=../data/20120413/F007793.dat&query=25084&hit=1&index=DYN1_MOUSE&px=1&section=5&ave_thresh=1&_ignoreionsscorebelow=20&report=0&_sigthreshold=0.05&_msresflags=1089&_msresflags2=2&percolate=-1&percolate_rt=0))  **602 - 617 983.4393 1964.8640 1964.8673 -2 0 R.QLELACETQEEVDSWK.A** ([Ions score 89](http://10.139.25.109/mascot/cgi/peptide_view.pl?file=../data/20120413/F007793.dat&query=39677&hit=1&index=DYN1_MOUSE&px=1&section=5&ave_thresh=1&_ignoreionsscorebelow=20&report=0&_sigthreshold=0.05&_msresflags=1089&_msresflags2=2&percolate=-1&percolate_rt=0))  **664 - 675 691.8534 1381.6922 1381.6911 1 0 R.NLVDSYMAIVNK.T**  Oxidation (M) ([Ions score 53](http://10.139.25.109/mascot/cgi/peptide_view.pl?file=../data/20120413/F007793.dat&query=25257&hit=1&index=DYN1_MOUSE&px=1&section=5&ave_thresh=1&_ignoreionsscorebelow=20&report=0&_sigthreshold=0.05&_msresflags=1089&_msresflags2=2&percolate=-1&percolate_rt=0))  **684 - 694 674.3408 1346.6671 1346.6686 -1 0 K.TIMHLMINNTK.E**  2 Oxidation (M) ([Ions score 52](http://10.139.25.109/mascot/cgi/peptide_view.pl?file=../data/20120413/F007793.dat&query=24461&hit=1&index=DYN1_MOUSE&px=1&section=5&ave_thresh=1&_ignoreionsscorebelow=20&report=0&_sigthreshold=0.05&_msresflags=1089&_msresflags2=2&percolate=-1&percolate_rt=0))  **684 - 694 449.8964 1346.6673 1346.6686 -1 0 K.TIMHLMINNTK.E**  2 Oxidation (M) ([Ions score 22](http://10.139.25.109/mascot/cgi/peptide_view.pl?file=../data/20120413/F007793.dat&query=24462&hit=1&index=DYN1_MOUSE&px=1&section=5&ave_thresh=1&_ignoreionsscorebelow=20&report=0&_sigthreshold=0.05&_msresflags=1089&_msresflags2=2&percolate=-1&percolate_rt=0))  **839 - 861 823.4332 2467.2779 2467.2832 -2 2 R.APPGVPSLGAWRLNSPQGKHENR.A**  ([Ions score 21](http://10.139.25.109/mascot/cgi/peptide_view.pl?file=../data/20120413/F007793.dat&query=44348&hit=1&index=DYN1_MOUSE&px=1&section=5&ave_thresh=1&_ignoreionsscorebelow=20&report=0&_sigthreshold=0.05&_msresflags=1089&_msresflags2=2&percolate=-1&percolate_rt=0))  18. [TSP1_MOUSE](http://10.139.25.109/mascot/cgi/protein_view.pl?file=../data/20120413/F007791.dat&hit=TSP1_MOUSE&db_idx=1&px=1&ave_thresh=1&_ignoreionsscorebelow=20&report=0&_sigthreshold=0.05&_msresflags=1089&_msresflags2=2&percolate=-1&percolate_rt=0)    **Mass:** 133555   **Score:** 574   **Matches:** 9(9)  **Sequences:** 9(9)  Thrombospondin-1  Sequence Coverage: **10%**; Matched peptides shown in **Bold Red**  **1** MELLRGLGVL FLLHMCGSNR IPESGGDNGV FDIFELIGGA RRGPGRRLVK GQDLSSPAFR **IENANLIPAV PDDKFQDLLD AVWADK**GFIF LASLRQMKKT  **101** RGTLLAVERK DNTGQIFSVV SNGK**AGTLDL SLSLPGK**QQV VSVEEALLAT GQWKSITLFV QEDRAQLYID CDKMESAELD VPIQSIFTRD LASVARLRVA  **201** KGDVNDNFQG VLQNVR**FVFG TTPEDILR**NK GCSSSTNVLL TLDNNVVNGS SPAIRTNYIG HKTKDLQAIC GLSCDELSSM VLELKGLR**TI VTTLQDSIR**K  **301** VTEENRELVS ELKRPPLCFH NGVQYKNNEE WTVDSCTECH CQNSVTICKK VSCPIMPCSN ATVPDGECCP RCWPSDSADD GWSPWSEWTS CSATCGNGIQ  **401** QRGRSCDSLN NRCEGSSVQT RTCHIQECDK RFKQDGGWSH WSPWSSCSVT CGDGVITRIR LCNSPSPQMN GKPCEGEARE TKACKKDACP INGGWGPWSP  **501** WDICSVTCGG GVQRRSR**LCN NPTPQFGGKD CVGDVTENQV CNK**QDCPIDG CLSNPCFAGA K**CTSYPDGSW KCGACPPGYS GNGIQCK**DVD ECKEVPDACF  **601** NHNGEHRCKN TDPGYNCLPC PPRFTGSQPF GRGVEHAMAN KQVCKPRNPC TDGTHDCNKN AKCNYLGHYS DPMYRCECKP GYAGNGIICG EDTDLDGWPN  **701** ENLVCVANAT YHCKKDNCPN LPNSGQEDYD KDGIGDACDD DDDNDKIPDD RDNCPFHYNP AQYDYDRDDV GDRCDNCPYN HNPDQADTDK NGEGDACAVD  **801** IDGDGILNER **DNCQYVYNVD QR**DTDMDGVG DQCDNCPLEH NPDQLDSDSD LIGDTCDNNQ DIDEDGHQNN LDNCPYVPNA NQADHDKDGK GDACDHDDDN  **901** DGIPDDRDNC RLVPNPDQKD SDGDGRGDAC KDDFDHDNVP DIDDICPENF DISETDFRRF QMIPLDPKGT SQNDPNWVVR HQGKELVQTV NCDPGLAVGY  **1001** DEFNAVDFSG TFFINTERDD DYAGFVFGYQ SSSRFYVVMW KQVTQSYWDT NPTRAQGYSG LSVKVVNSTT GPGEHLRNAL WHTGNTPGQV RTLWHDPRHI  **1101** GWKDFTAYRW RLSHRPKTGY IRVVMYEGKK IMADSGPIYD KTYAGGRLGL FVFSQEMVFF SDMKYECRDS  **Start - End Observed Mr(expt) Mr(calc) ppm Miss Sequence**  **61 - 86 970.8356 2909.4834 2909.4811 1 1 R.IENANLIPAVPDDKFQDLLDAVWADK.G** ([Ions score 99](http://10.139.25.109/mascot/cgi/peptide_view.pl?file=../data/20120413/F007791.dat&query=52414&hit=1&index=TSP1_MOUSE&px=1&section=5&ave_thresh=1&_ignoreionsscorebelow=20&report=0&_sigthreshold=0.05&_msresflags=1089&_msresflags2=2&percolate=-1&percolate_rt=0))  **125 - 137 636.3640 1270.7124 1270.7133 1 0 K.AGTLDLSLSLPGK.Q**  ([Ions score 76](http://10.139.25.109/mascot/cgi/peptide_view.pl?file=../data/20120413/F007791.dat&query=15429&hit=1&index=TSP1_MOUSE&px=1&section=5&ave_thresh=1&_ignoreionsscorebelow=20&report=0&_sigthreshold=0.05&_msresflags=1089&_msresflags2=2&percolate=-1&percolate_rt=0))  **217 - 228 697.8699 1393.7252 1393.7242 1 0 R.FVFGTTPEDILR.N**  ([Ions score 45](http://10.139.25.109/mascot/cgi/peptide_view.pl?file=../data/20120413/F007791.dat&query=19503&hit=1&index=TSP1_MOUSE&px=1&section=5&ave_thresh=1&_ignoreionsscorebelow=20&report=0&_sigthreshold=0.05&_msresflags=1089&_msresflags2=2&percolate=-1&percolate_rt=0))  **289 - 299 623.8541 1245.6926 1245.6929 0 0 R.TIVTTLQDSIR.K**  ([Ions score 65](http://10.139.25.109/mascot/cgi/peptide_view.pl?file=../data/20120413/F007791.dat&query=14276&hit=1&index=TSP1_MOUSE&px=1&section=5&ave_thresh=1&_ignoreionsscorebelow=20&report=0&_sigthreshold=0.05&_msresflags=1089&_msresflags2=2&percolate=-1&percolate_rt=0))  **518 - 529 666.8214 1331.6281 1331.6292 -1 0 R.LCNNPTPQFGGK.D**  ([Ions score 35](http://10.139.25.109/mascot/cgi/peptide_view.pl?file=../data/20120413/F007791.dat&query=17812&hit=1&index=TSP1_MOUSE&px=1&section=5&ave_thresh=1&_ignoreionsscorebelow=20&report=0&_sigthreshold=0.05&_msresflags=1089&_msresflags2=2&percolate=-1&percolate_rt=0))  **530 - 543 819.3477 1636.6808 1636.6821 -1 0 K.DCVGDVTENQVCNK.Q**  ([Ions score 110](http://10.139.25.109/mascot/cgi/peptide_view.pl?file=../data/20120413/F007791.dat&query=27509&hit=1&index=TSP1_MOUSE&px=1&section=5&ave_thresh=1&_ignoreionsscorebelow=20&report=0&_sigthreshold=0.05&_msresflags=1089&_msresflags2=2&percolate=-1&percolate_rt=0))  **562 - 571 600.7539 1199.4931 1199.4917 1 0 K.CTSYPDGSWK.C**  ([Ions score 35](http://10.139.25.109/mascot/cgi/peptide_view.pl?file=../data/20120413/F007791.dat&query=12108&hit=1&index=TSP1_MOUSE&px=1&section=5&ave_thresh=1&_ignoreionsscorebelow=20&report=0&_sigthreshold=0.05&_msresflags=1089&_msresflags2=2&percolate=-1&percolate_rt=0))  **572 - 587 863.3497 1724.6838 1724.6836 0 0 K.CGACPPGYSGNGIQCK.D** ([Ions score 43](http://10.139.25.109/mascot/cgi/peptide_view.pl?file=../data/20120413/F007791.dat&query=30615&hit=1&index=TSP1_MOUSE&px=1&section=5&ave_thresh=1&_ignoreionsscorebelow=20&report=0&_sigthreshold=0.05&_msresflags=1089&_msresflags2=2&percolate=-1&percolate_rt=0))  **811 - 822 787.3391 1572.6637 1572.6627 1 0 R.DNCQYVYNVDQR.D**  ([Ions score 66](http://10.139.25.109/mascot/cgi/peptide_view.pl?file=../data/20120413/F007791.dat&query=25178&hit=1&index=TSP1_MOUSE&px=1&section=5&ave_thresh=1&_ignoreionsscorebelow=20&report=0&_sigthreshold=0.05&_msresflags=1089&_msresflags2=2&percolate=-1&percolate_rt=0))  19. [FKBP4_MOUSE](http://10.139.25.109/mascot/cgi/protein_view.pl?file=../data/20120413/F007793.dat&hit=FKBP4_MOUSE&db_idx=1&px=1&ave_thresh=1&_ignoreionsscorebelow=20&report=0&_sigthreshold=0.05&_msresflags=1089&_msresflags2=2&percolate=-1&percolate_rt=0)    **Mass:** 51939    **Score:** 290    **Matches:** 6(6)  **Sequences:** 6(6)  Peptidyl-prolyl cis-trans isomerase FKBP4  Sequence Coverage: **14%;** Matched peptides shown in **Bold Red**  **1** MTAEEMKAAE NGAQSAPLPL EGVDISPKQD EGVLKVIKRE GTGTETPMIG DRVFVHYTGW LLDGTKFDSS LDRKDKFSFD LGKGEVIK**AW DIAVATMK**VG  **101** EVCHITCKPE YAYGAAGSPP KIPPNATLVF EVELFEFKGE DLTEEEDGGI IRRIRTRGEG YARPNDGAMV EVALEGYHKD RLFDQRELCF EVGEGESLDL  **201** PCGLEEAIQR MEKGEHSIVY LKPSYAFGSV GKERFQIPPH AELRYEVRLK SFEKAKESWE MSSAEKLEQS NIVKERGTAY FKEGKYKQAL LQYKKIVSWL  **301** EYESSFSGEE MQKVHALRLA SHLNLAMCHL K**LQAFSAAIE SCNKALELDS NNEK**GLFR**RG EAHLAVNDFD LAR**ADFQK**VL QLYPSNK**AAK TQLAVCQQRT  **401** RRQLAREKK**L YANMFER**LAE EEHKVKAEVA AGDHPTDAEM KGERNNVAEN QSRVETEA  **Start - End Observed Mr(expt) Mr(calc) ppm Miss Sequence**  **89 - 98 553.2914 1104.5682 1104.5638 4 0 K.AWDIAVATMK.V**  ([Ions score 24](http://10.139.25.109/mascot/cgi/peptide_view.pl?file=../data/20120413/F007793.dat&query=13583&hit=1&index=FKBP4_MOUSE&px=1&section=5&ave_thresh=1&_ignoreionsscorebelow=20&report=0&_sigthreshold=0.05&_msresflags=1089&_msresflags2=2&percolate=-1&percolate_rt=0))  **332 - 344 719.8557 1437.6969 1437.6922 3 0 K.LQAFSAAIESCNK.A**  ([Ions score 84](http://10.139.25.109/mascot/cgi/peptide_view.pl?file=../data/20120413/F007793.dat&query=26548&hit=1&index=FKBP4_MOUSE&px=1&section=5&ave_thresh=1&_ignoreionsscorebelow=20&report=0&_sigthreshold=0.05&_msresflags=1089&_msresflags2=2&percolate=-1&percolate_rt=0))  **345 - 354 566.7777 1131.5409 1131.5407 0 0 K.ALELDSNNEK.G**  ([Ions score 56](http://10.139.25.109/mascot/cgi/peptide_view.pl?file=../data/20120413/F007793.dat&query=14990&hit=1&index=FKBP4_MOUSE&px=1&section=5&ave_thresh=1&_ignoreionsscorebelow=20&report=0&_sigthreshold=0.05&_msresflags=1089&_msresflags2=2&percolate=-1&percolate_rt=0))  **359 - 373 561.9570 1682.8493 1682.8488 0 1 R.RGEAHLAVNDFDLAR.A**  ([Ions score 48](http://10.139.25.109/mascot/cgi/peptide_view.pl?file=../data/20120413/F007793.dat&query=33079&hit=1&index=FKBP4_MOUSE&px=1&section=5&ave_thresh=1&_ignoreionsscorebelow=20&report=0&_sigthreshold=0.05&_msresflags=1089&_msresflags2=2&percolate=-1&percolate_rt=0))  **379 - 387 531.3034 1060.5923 1060.5917 1 0 K.VLQLYPSNK.A**  ([Ions score 47](http://10.139.25.109/mascot/cgi/peptide_view.pl?file=../data/20120413/F007793.dat&query=11656&hit=1&index=FKBP4_MOUSE&px=1&section=5&ave_thresh=1&_ignoreionsscorebelow=20&report=0&_sigthreshold=0.05&_msresflags=1089&_msresflags2=2&percolate=-1&percolate_rt=0))  **410 - 417 522.2531 1042.4917 1042.4906 1 0 K.LYANMFER.L**  ([Ions score 31](http://10.139.25.109/mascot/cgi/peptide_view.pl?file=../data/20120413/F007793.dat&query=10870&hit=1&index=FKBP4_MOUSE&px=1&section=5&ave_thresh=1&_ignoreionsscorebelow=20&report=0&_sigthreshold=0.05&_msresflags=1089&_msresflags2=2&percolate=-1&percolate_rt=0))  20. [NRP1_MOUSE](http://10.139.25.109/mascot/cgi/protein_view.pl?file=../data/20120413/F007793.dat&hit=NRP1_MOUSE&db_idx=1&px=1&ave_thresh=1&_ignoreionsscorebelow=20&report=0&_sigthreshold=0.05&_msresflags=1089&_msresflags2=2&percolate=-1&percolate_rt=0)    **Mass:** 104189   **Score:** 545    **Matches:** 12(12)  **Sequences:** 12(12)  Neuropilin-1 OS=Mus musculus  Sequence Coverage: **18%**; Matched peptides shown in **Bold Red**  **1** MERGLPLLCA TLALALALAG AFRSDKCGGT IKIENPGYLT SPGYPHSYHP SEK**CEWLIQA PEPYQR**IMIN FNPHFDLEDR DCKYDYVEVI DGENEGGRLW  **101** GKFCGK**IAPS PVVSSGPFLF IK**FVSDYETH GAGFSIRYEI FKRGPECSQN YTAPTGVIK**S PGFPEKYPNS LECTYIIFAP K**MSEIILEFE SFDLEQDSNP  **201** PGGMFCRYDR LEIWDGFPEV GPHIGRYCGQ KTPGRIRSSS GVLSMVFYTD SAIAKEGFSA NYSVLQSSIS EDFKCMEALG MESGEIHSDQ ITASSQYGTN  **301** WSVERSRLNY PENGWTPGED SYKEWIQVDL GLLR**FVTAVG TQGAISK**ETK KKYYVKTYRV DISSNGEDWI SLKEGNK**AII FQGNTNPTDV VLGVFSKPLI**  **401 TR**FVR**IKPVS WETGISMRFE VYGCK**ITDYP CSGMLGMVSG LISDSQITAS NQADRNWMPE NIRLVTSRTG WALPPSPHPY TNEWLQVDLG DEKIVR**GVII**  **501 QGGK**HRENKV FMRKFKIAYS NNGSDWKTIM DDSKRKAKSF EGNNNYDTPE LR**TFSPLSTR** FIRIYPERAT HSGLGLRMEL LGCEVEAPTA GPTTPNGNPV  **601** DECDDDQANC HSGTGDDFQL TGGTTVLATE KPTIIDSTIQ SEFPTYGFNC EFGWGSHKTF CHWEHDSHAQ LRWSVLTSK**T GPIQDHTGDG NFIYSQADEN**  **701 QK**GKVARLVS PVVYSQSSAH CMTFWYHMSG SHVGTLRVKL RYQKPEEYDQ LVWMVVGHQG DHWKEGRVLL HKSLKLYQVI FEGEIGKGNL GGIAVDDISI  **801** NNHISQEDCA KPTDLDK**KNT EIK**IDETGST PGYEGEGEGD KNISRKPGNV LKTLDPILIT IIAMSALGVL LGAVCGVVLY CACWHNGMSE R**NLSALENYN**  **901 FELVDGVK**LK KDKLNPQSNY SEA  **Start - End Observed Mr(expt) Mr(calc) ppm Miss Sequence**  **54 - 66 845.4062 1688.7978 1688.7981 0 0 K.CEWLIQAPEPYQR.I**  ([Ions score 43](http://10.139.25.109/mascot/cgi/peptide_view.pl?file=../data/20120413/F007793.dat&query=33205&hit=1&index=NRP1_MOUSE&px=1&section=5&ave_thresh=1&_ignoreionsscorebelow=20&report=0&_sigthreshold=0.05&_msresflags=1089&_msresflags2=2&percolate=-1&percolate_rt=0))  **107 - 122 829.9806 1657.9467 1657.9443 1 0 K.IAPSPVVSSGPFLFIK.F**  ([Ions score 81](http://10.139.25.109/mascot/cgi/peptide_view.pl?file=../data/20120413/F007793.dat&query=32545&hit=1&index=NRP1_MOUSE&px=1&section=5&ave_thresh=1&_ignoreionsscorebelow=20&report=0&_sigthreshold=0.05&_msresflags=1089&_msresflags2=2&percolate=-1&percolate_rt=0))  **160 - 181 853.4268 2557.2570 2557.2563 0 1 K.SPGFPEKYPNSLECTYIIFAPK.M** ([Ions score 49](http://10.139.25.109/mascot/cgi/peptide_view.pl?file=../data/20120413/F007793.dat&query=45031&hit=1&index=NRP1_MOUSE&px=1&section=5&ave_thresh=1&_ignoreionsscorebelow=20&report=0&_sigthreshold=0.05&_msresflags=1089&_msresflags2=2&percolate=-1&percolate_rt=0))  **335 - 347 639.8546 1277.6945 1277.6980 -3 0 R.FVTAVGTQGAISK.E**  ([Ions score 89](http://10.139.25.109/mascot/cgi/peptide_view.pl?file=../data/20120413/F007793.dat&query=22125&hit=1&index=NRP1_MOUSE&px=1&section=5&ave_thresh=1&_ignoreionsscorebelow=20&report=0&_sigthreshold=0.05&_msresflags=1089&_msresflags2=2&percolate=-1&percolate_rt=0))  **378 - 402 900.8415 2699.5011 2699.5010 0 0 K.AIIFQGNTNPTDVVLGVFSKPLITR.F** ([Ions score 59](http://10.139.25.109/mascot/cgi/peptide_view.pl?file=../data/20120413/F007793.dat&query=45956&hit=1&index=NRP1_MOUSE&px=1&section=5&ave_thresh=1&_ignoreionsscorebelow=20&report=0&_sigthreshold=0.05&_msresflags=1089&_msresflags2=2&percolate=-1&percolate_rt=0))  **406 - 418 501.9377 1502.7912 1502.7915 0 0 R.IKPVSWETGISMR.F**  ([Ions score 36](http://10.139.25.109/mascot/cgi/peptide_view.pl?file=../data/20120413/F007793.dat&query=28035&hit=1&index=NRP1_MOUSE&px=1&section=5&ave_thresh=1&_ignoreionsscorebelow=20&report=0&_sigthreshold=0.05&_msresflags=1089&_msresflags2=2&percolate=-1&percolate_rt=0))  **419 - 425 451.7067 901.3988 901.4004 -2 0 R.FEVYGCK.I**  ([Ions score 21](http://10.139.25.109/mascot/cgi/peptide_view.pl?file=../data/20120413/F007793.dat&query=5143&hit=1&index=NRP1_MOUSE&px=1&section=5&ave_thresh=1&_ignoreionsscorebelow=20&report=0&_sigthreshold=0.05&_msresflags=1089&_msresflags2=2&percolate=-1&percolate_rt=0))  **497 - 504 386.7335 771.4525 771.4491 4 0 R.GVIIQGGK.H**  Deamidated (NQ) ([Ions score 23](http://10.139.25.109/mascot/cgi/peptide_view.pl?file=../data/20120413/F007793.dat&query=1416&hit=1&index=NRP1_MOUSE&px=1&section=5&ave_thresh=1&_ignoreionsscorebelow=20&report=0&_sigthreshold=0.05&_msresflags=1089&_msresflags2=2&percolate=-1&percolate_rt=0))  **553 - 560 454.7446 907.4746 907.4763 -2 0 R.TFSPLSTR.F**  ([Ions score 24](http://10.139.25.109/mascot/cgi/peptide_view.pl?file=../data/20120413/F007793.dat&query=5381&hit=1&index=NRP1_MOUSE&px=1&section=5&ave_thresh=1&_ignoreionsscorebelow=20&report=0&_sigthreshold=0.05&_msresflags=1089&_msresflags2=2&percolate=-1&percolate_rt=0))  **680 - 702 845.7174 2534.1288 2534.1310 1 0 K.TGPIQDHTGDGNFIYSQADENQK.G**  ([Ions score 61](http://10.139.25.109/mascot/cgi/peptide_view.pl?file=../data/20120413/F007793.dat&query=44835&hit=1&index=NRP1_MOUSE&px=1&section=5&ave_thresh=1&_ignoreionsscorebelow=20&report=0&_sigthreshold=0.05&_msresflags=1089&_msresflags2=2&percolate=-1&percolate_rt=0))  **818 - 823 366.7163 731.4170 731.4177 1 1 K.KNTEIK.I**  ([Ions score 26](http://10.139.25.109/mascot/cgi/peptide_view.pl?file=../data/20120413/F007793.dat&query=560&hit=1&index=NRP1_MOUSE&px=1&section=5&ave_thresh=1&_ignoreionsscorebelow=20&report=0&_sigthreshold=0.05&_msresflags=1089&_msresflags2=2&percolate=-1&percolate_rt=0))  **892 - 908 962.9869 1923.9593 1923.9578 1 0 R.NLSALENYNFELVDGVK.L**  ([Ions score 33](http://10.139.25.109/mascot/cgi/peptide_view.pl?file=../data/20120413/F007793.dat&query=38875&hit=1&index=NRP1_MOUSE&px=1&section=5&ave_thresh=1&_ignoreionsscorebelow=20&report=0&_sigthreshold=0.05&_msresflags=1089&_msresflags2=2&percolate=-1&percolate_rt=0))  21. [TCOF_MOUSE](http://10.139.25.109/mascot/cgi/protein_view.pl?file=../data/20120413/F007791.dat&hit=TCOF_MOUSE&db_idx=1&px=1&ave_thresh=1&_ignoreionsscorebelow=20&report=0&_sigthreshold=0.05&_msresflags=1089&_msresflags2=2&percolate=-1&percolate_rt=0)    **Mass:** 135035   **Score:** 204    **Matches:** 5(5)  **Sequences:** 5(5)  Treacle protein  Sequence Coverage: **5%**; Matched peptides shown in **Bold Red**  **1** MAEARKRREL LPLIYHHLLQ AGYVRAAREV KEQSGQK**SFL TQPVTLLDIY THWQQTSELG QK**QKAEDDET LQAKKSRVSD PVSSSESSDQ EKEEEAATER  **101** AKATPRPTPV NSATAALPSK VKEKGKTKTA NKTVNSVSHP GSGKTVVHLL SGKSPKKSAE PLANTVLASE TEEEGNAQAL GPTAKSGTVS AGQGSSSSED  **201** SSISSDETDV EVKSPAKPAQ AKASAAPAKD PPARTAPGPT KLGNVAPTPA KPARAAAAAA AAAVAAAAAA AAEESESSEE DSDSEDEAPA GLPSQVKASG  **301** KGPHVRADSV SAKGISGKGP ILATPGKTGP AATQAKAERP EKDSETSSED DSDSEDEMPV TVNTPQARTS GKSPRARGTS APAKESSQKG APAVTPGKAR  **401** PVAAQAGKPE AKSSEESESD SGETPAAATL TTSPAKVKPL GKSSQVRPVS TVTPGSSGKG ANLPCPGKVG SAALRVQMVK KEDVSESSSA ELDSDGPGSP  **501** AKAKASLALP QKVRPVATQV KTDRGKGHSG SSEESSDSEE EAAPAASAAQ AKPALEKQMK ASSRKGTPAS ATGASTSSHC KAGAVTSSAS LSSPALAKGT  **601** QRSDVDSSSE SESEGAAPST PRVQGKSGGK GLQGKAALGQ GVAPVHTQKT GPSVKAMAQE DSESLEEDSS SEEEDETPAQ ATPLGRLPQA KANPPPTKTP  **701** PASASGKAVA APTKGKPPVP NSTVSARGQR SVPAAGKAGA PATQAQKGPV AGTGEDSESS SKEESDSEEE TPAQIKPVGK TSQVRAASAP AKESPKKGAH  **801** PGTPGKTGSS ATQAQPGKTE DSDSSSEESD SDTEMPSAQA IKSPPVSVNR NSSPAVPAPT PEGVQAVNTT KKASGTTAQS SSSESEDGDE DLIPATQPST  **901** YALRTSVTTP AALSRAASQP SKSEQSSRMP KGKKAKAAAS AQTSSAVETL PMMPPQSAPI QPKATNKLGK SKLPEKQQLA PGYPKAPRSS EDSSDTSSED  **1001** EEDAKRPQMP KSAHRLDPDP SQKETVVEET PTESSEDEMV APSQSLLSGY MTPGLTVANS QASKATPRPD SNSLASSAPA TKDNPDGKQK SKSQHAADTA  **1101** LPKTGRK**EAS SGSTPQKPK**K LKKSTSSSPA PTQTLPNSIT QRLLEQAWPL SEAQVQASVV K**VLTELLEQE R**LKATEAIK**E SGKKSQK**RK**L SGDLEAGAPK**  **1201** NKKKKEQPVP RASAVSPEKA PMTSKAKSKL DKGSAGGKGK GSPGPQGAKE KPDGELLGIK LESGEQSDPK SKSKKKKSLK KKKDKEKKEK KKGKKSLAKD  1301 SASPIQKKKK KKKKSAEPAV  **Start - End Observed Mr(expt) Mr(calc) ppm Miss Sequence**  **38 - 62 978.5071 2932.4979 2932.4971 0 0 K.SFLTQPVTLLDIYTHWQQTSELGQK.Q**  ([Ions score 57](http://10.139.25.109/mascot/cgi/peptide_view.pl?file=../data/20120413/F007791.dat&query=52584&hit=1&index=TCOF_MOUSE&px=1&section=5&ave_thresh=1&_ignoreionsscorebelow=20&report=0&_sigthreshold=0.05&_msresflags=1089&_msresflags2=2&percolate=-1&percolate_rt=0))  **1108 - 1119 608.8153 1215.6150 1215.6095 5 0 K.EASSGSTPQKPK.K**  ([Ions score 35](http://10.139.25.109/mascot/cgi/peptide_view.pl?file=../data/20120413/F007791.dat&query=12746&hit=1&index=TCOF_MOUSE&px=1&section=5&ave_thresh=1&_ignoreionsscorebelow=20&report=0&_sigthreshold=0.05&_msresflags=1089&_msresflags2=2&percolate=-1&percolate_rt=0))  **1162 - 1171 615.3393 1228.6640 1228.6663 -2 0 K.VLTELLEQER.L**  ([Ions score 65](http://10.139.25.109/mascot/cgi/peptide_view.pl?file=../data/20120413/F007791.dat&query=13388&hit=1&index=TCOF_MOUSE&px=1&section=5&ave_thresh=1&_ignoreionsscorebelow=20&report=0&_sigthreshold=0.05&_msresflags=1089&_msresflags2=2&percolate=-1&percolate_rt=0))  **1180 - 1187 446.2510 890.4874 890.4821 6 2 K.ESGKKSQK.R**  ([Ions score 24](http://10.139.25.109/mascot/cgi/peptide_view.pl?file=../data/20120413/F007793.dat&query=4788&hit=1&index=TCOF_MOUSE&px=1&section=5&ave_thresh=1&_ignoreionsscorebelow=20&report=0&_sigthreshold=0.05&_msresflags=1089&_msresflags2=2&percolate=-1&percolate_rt=0))  **1190 - 1200 529.2788 1056.5431 1056.5451 -2 0 K.LSGDLEAGAPK.N**  ([Ions score 23](http://10.139.25.109/mascot/cgi/peptide_view.pl?file=../data/20120413/F007793.dat&query=11468&hit=1&index=TCOF_MOUSE&px=1&section=5&ave_thresh=1&_ignoreionsscorebelow=20&report=0&_sigthreshold=0.05&_msresflags=1089&_msresflags2=2&percolate=-1&percolate_rt=0))  22. [YBOX2_MOUSE](http://10.139.25.109/mascot/cgi/protein_view.pl?file=../data/20120413/F007791.dat&hit=YBOX2_MOUSE&db_idx=1&px=1&ave_thresh=1&_ignoreionsscorebelow=20&report=0&_sigthreshold=0.05&_msresflags=1089&_msresflags2=2&percolate=-1&percolate_rt=0)    **Mass:** 38248    **Score:** 315    **Matches:** 6(6)  **Sequences:** 5(5)  Y-box-binding protein 2  Sequence Coverage: **12%**; Matched peptides shown in **Bold Red**  **1** MSEAEASVVA TAAPAATVPA TAAGVVAVVV PVPAGEPQKA GGGAGGGGGA ASGPAAGTPL HAPGPRTPGN QATAASGTPA PPARSQADKP VLAIQVLGTV  **101** K**WFNVRNGYG FINRNDTKED VFVHQTAIK**R NNPRKFLR**SV GDGETVEFDV VEGEK**GARAA NVTGPGGVPV KGSRYAPNRR RFRRFIPRPR PAAPPPMVAE  **201** APSGGTEPGS EGERAEDSGQ RPRRRRPPPF FYRRRFVRGP RPPNQQQPIE GSDGVEPKET APLEGDQQQG DERVPPPRFR PRYRRPFRPR PPQQPTTEGG  **301** DGETKPSQGP TDGSRPEPQR PRNRPYFQRR RQQPPGPRQP IAAETSAPIN SGDPPTTILE    **Start - End Observed Mr(expt) Mr(calc) ppm Miss Sequence**  **102 - 106 361.1927 720.3709 720.3707 0 0 K.WFNVR.N**  ([Ions score 22](http://10.139.25.109/mascot/cgi/peptide_view.pl?file=../data/20120413/F007791.dat&query=139&hit=1&index=YBOX2_MOUSE&px=1&section=5&ave_thresh=1&_ignoreionsscorebelow=20&report=0&_sigthreshold=0.05&_msresflags=1089&_msresflags2=2&percolate=-1&percolate_rt=0))  **107 - 114 470.7346 939.4547 939.4563 -2 0 R.NGYGFINR.N**  ([Ions score 42](http://10.139.25.109/mascot/cgi/peptide_view.pl?file=../data/20120413/F007791.dat&query=3229&hit=1&index=YBOX2_MOUSE&px=1&section=5&ave_thresh=1&_ignoreionsscorebelow=20&report=0&_sigthreshold=0.05&_msresflags=1089&_msresflags2=2&percolate=-1&percolate_rt=0))  **107 - 114 471.2276 940.4406 940.4403 0 0 R.NGYGFINR.N**  Deamidated (NQ) ([Ions score 48](http://10.139.25.109/mascot/cgi/peptide_view.pl?file=../data/20120413/F007791.dat&query=3259&hit=1&index=YBOX2_MOUSE&px=1&section=5&ave_thresh=1&_ignoreionsscorebelow=20&report=0&_sigthreshold=0.05&_msresflags=1089&_msresflags2=2&percolate=-1&percolate_rt=0))  **107 - 129 667.5880 2666.3229 2666.3089 5 2 R.NGYGFINRNDTKEDVFVHQTAIK.R**  Deamidated (NQ) ([Ions score 23](http://10.139.25.109/mascot/cgi/peptide_view.pl?file=../data/20120413/F007791.dat&query=50019&hit=1&index=YBOX2_MOUSE&px=1&section=5&ave_thresh=1&_ignoreionsscorebelow=20&report=0&_sigthreshold=0.05&_msresflags=1089&_msresflags2=2&percolate=-1&percolate_rt=0))  **115 - 129 582.2999 1743.8778 1743.8792 -1 1 R.NDTKEDVFVHQTAIK.R**  ([Ions score 47](http://10.139.25.109/mascot/cgi/peptide_view.pl?file=../data/20120413/F007791.dat&query=31289&hit=1&index=YBOX2_MOUSE&px=1&section=5&ave_thresh=1&_ignoreionsscorebelow=20&report=0&_sigthreshold=0.05&_msresflags=1089&_msresflags2=2&percolate=-1&percolate_rt=0))  **139 - 155 898.4148 1794.8150 1794.8160 -1 0 R.SVGDGETVEFDVVEGEK.G**  ([Ions score 133](http://10.139.25.109/mascot/cgi/peptide_view.pl?file=../data/20120413/F007791.dat&query=33279&hit=1&index=YBOX2_MOUSE&px=1&section=5&ave_thresh=1&_ignoreionsscorebelow=20&report=0&_sigthreshold=0.05&_msresflags=1089&_msresflags2=2&percolate=-1&percolate_rt=0))  23. [MDR1B_MOUSE](http://10.139.25.109/mascot/cgi/protein_view.pl?file=../data/20120413/F007793.dat&hit=MDR1B_MOUSE&db_idx=1&px=1&ave_thresh=1&_ignoreionsscorebelow=20&report=0&_sigthreshold=0.05&_msresflags=1089&_msresflags2=2&percolate=-1&percolate_rt=0)    **Mass:** 141475   **Score:** 358    **Matches:** 6(6)  **Sequences:** 6(6)  Multidrug resistance protein 1B  Sequence Coverage: **8%**; Matched peptides shown in **Bold Red**  **1** MEFEENLKGR ADKNFSKMGK KSKKEKKEKK PAVGVFGMFR YADWLDKLCM ILGTLAAIIH GTLLPLLMLV FGNMTDSFTK AEASILPSIT NQSGPNSTLI  **101** ISNSSLEEEM AIYAYYYTGI GAGVLIVAYI QVSLWCLAAG RQIHKIRQKF FHAIMNQEIG WFDVHDVGEL NTRLTDDVSK INDGIGDKIG MFFQSITTFL  **201** AGFIIGFISG WKLTLVILAV SPLIGLSSAL WAKVLTSFTN KELQAYAKAG AVAEEVLAAI RTVIAFGGQQ KELERYNKNL EEAKNVGIKK AITASISIGI  **301** AYLLVYASYA LAFWYGTSLV LSNEYSIGEV LTVFFSILLG TFSIGHLAPN IEAFANARGA AFEIFKIIDN EPSIDSFSTK GYKPDSIMGN LEFKNVHFNY  **401** PSRSEVQILK GLNLKVKSGQ TVALVGNSGC GKSTTVQLMQ R**LYDPLEGVV SIDGQDIR**TI NVRYLR**EIIG VVSQEPVLFA TTIAENIR**YG REDVTMDEIE  **501** KAVKEANAYD FIMKLPHQFD TLVGERGAQL SGGQKQRIAI ARALVRNPK**I LLLDEATSAL DTESEAVVQA ALDK**AREGRT TIVIAHRLST VRNADVIAGF  **601** DGGVIVEQGN HDELMREKGI YFKLVMTQTR GNEIEPGNNA YGSQSDTDAS ELTSEESKSP LIRRSIYRSV HRKQDQERRL SMKEAVDEDV PLVSFWRILN  **701** LNLSEWPYLL VGVLCAVING CIQPVFAIVF SRIVGVFSRD DDHETKRQNC NLFSLFFLVM GLISFVTYFF QGFTFGK**AGE ILTKR**VRYMV FKSMLRQDIS  **801** WFDDHKNSTG SLTTRLASDA SSVKGAMGAR LAVVTQNVAN LGTGVILSLV YGWQLTLLLV VIIPLIVLGG IIEMKLLSGQ ALKDKKQLEI SGK**IATEAIE**  **901 NFR**TIVSLTR EQKFETMYAQ SLQVPYRNAM KKAHVFGITF SFTQAMMYFS YAACFRFGAY LVAQQLMTFE NVMLVFSAVV FGAMAAGNTS SFAPDYAKAK  **1001** VSASHIIRII EKTPEIDSYS TEGLKPTLLE GNVK**FNGVQF NYPTRPNIPV LQGLSLEVK**K GQTLALVGSS GCGKSTVVQL LERFYDPMAG SVFLDGKEIK  **1101** QLNVQWLRAH LGIVSQEPIL FDCSIAENIA YGDNSRAVSH EEIVRAAKEA NIHQFIDSLP DKYNTRVGDK GTQLSGGQKQ RIAIARALVR QPHILLLDEA  **1201** TSALDTESEK VVQEALDKAR EGRTCIVIAH RLSTIQNADL IVVIENGKVK EHGTHQQLLA QKGIYFSMVQ AGAKRS  **Start - End Observed Mr(expt) Mr(calc) ppm Miss Sequence**  **442 - 458 944.9867 1887.9578 1887.9578 0 0 R.LYDPLEGVVSIDGQDIR.T**  [Ions score 105](http://10.139.25.109/mascot/cgi/peptide_view.pl?file=../data/20120413/F007793.dat&query=38231&hit=1&index=MDR1B_MOUSE&px=1&section=5&ave_thresh=1&_ignoreionsscorebelow=20&report=0&_sigthreshold=0.05&_msresflags=1089&_msresflags2=2&percolate=-1&percolate_rt=0))  **467 - 488 800.4448 2398.3110 2398.3108 0 0 R.EIIGVVSQEPVLFATTIAENIR.Y**([Ions score 48](http://10.139.25.109/mascot/cgi/peptide_view.pl?file=../data/20120413/F007793.dat&query=43960&hit=1&index=MDR1B_MOUSE&px=1&section=5&ave_thresh=1&_ignoreionsscorebelow=20&report=0&_sigthreshold=0.05&_msresflags=1089&_msresflags2=2&percolate=-1&percolate_rt=0))  **550 - 574 872.4605 2614.3581 2614.3589 0 0 K.ILLLDEATSALDTESEAVVQAALDK.A**  [Ions score 60](http://10.139.25.109/mascot/cgi/peptide_view.pl?file=../data/20120413/F007793.dat&query=45497&hit=1&index=MDR1B_MOUSE&px=1&section=5&ave_thresh=1&_ignoreionsscorebelow=20&report=0&_sigthreshold=0.05&_msresflags=1089&_msresflags2=2&percolate=-1&percolate_rt=0))  **778 - 785 444.2687 886.5228 886.5236 -1 1 K.AGEILTKR.V**  ([Ions score 27](http://10.139.25.109/mascot/cgi/peptide_view.pl?file=../data/20120413/F007793.dat&query=4675&hit=1&index=MDR1B_MOUSE&px=1&section=5&ave_thresh=1&_ignoreionsscorebelow=20&report=0&_sigthreshold=0.05&_msresflags=1089&_msresflags2=2&percolate=-1&percolate_rt=0))  **894 - 903 582.3061 1162.5976 1162.5982 0 0 K.IATEAIENFR.T**  ([Ions score 63](http://10.139.25.109/mascot/cgi/peptide_view.pl?file=../data/20120413/F007793.dat&query=16495&hit=1&index=MDR1B_MOUSE&px=1&section=5&ave_thresh=1&_ignoreionsscorebelow=20&report=0&_sigthreshold=0.05&_msresflags=1089&_msresflags2=2&percolate=-1&percolate_rt=0))  **1035 - 1059 944.1811 2829.5199 2829.5177 1 0 K.FNGVQFNYPTRPNIPVLQGLSLEVK.K** ([Ions score 55](http://10.139.25.109/mascot/cgi/peptide_view.pl?file=../data/20120413/F007793.dat&query=46533&hit=1&index=MDR1B_MOUSE&px=1&section=5&ave_thresh=1&_ignoreionsscorebelow=20&report=0&_sigthreshold=0.05&_msresflags=1089&_msresflags2=2&percolate=-1&percolate_rt=0))  24. [FBLN2_MOUSE](http://10.139.25.109/mascot/cgi/protein_view.pl?file=../data/20120413/F007791.dat&hit=FBLN2_MOUSE&db_idx=1&px=1&ave_thresh=1&_ignoreionsscorebelow=20&report=0&_sigthreshold=0.05&_msresflags=1089&_msresflags2=2&percolate=-1&percolate_rt=0)    **Mass:** 137832   **Score:** 149    **Matches:** 3(3)  **Sequences:** 3(3)  Fibulin-2  Sequence Coverage: **4%**; Matched peptides shown in **Bold Red**  **1** MLLQESAGVW LALALVTALT PSPSMAVPWQ DCTGAECPLL ENCIEEALEP GACCATCVQQ GCACEGYQYY DCVQGGFVDG RVPAGQSYFV DFGSTECSCP  **101** PGGGKISCQF MLCPELPPNC IEAVVVADSC PQCGQVGCVH SGRKYAAGHT VHLSSCRACH CPDAGGELIC YQLPGCHGNF SDAEEGDSER QYEDPYSYDQ  **201** EVAEAEATTA IVNEVQAGAE GPPAALGGGN LPPSSIRVTP WPVALPRPTA AAALGPPAPV QAKARRVTLD TEEDEEEEEE ETLVTEPPTA GSPGRLDSLP  **301** TRSPARPGFP VQEKEAEAKA GPEENLIPDA QVTPRSVMQE GAAPVPRSGL AALSPSLATD SSSEDPVKPS DHPTLSTLPP DR**AQVSPSPE TPEEIPQHPQ**  **401 LLPR**FRAEED IDPNSVHSVP RGDLDGSTKD LIETCCAAGQ QWAIDNDECQ EIPENGAQSD ICRIAQRQCC ISYLKEKSCV AGVMGAKEGE TCGAEDNDTC  **501** GVSLYKQCCD CCGLGLRVRA EGQSCESNPN LGYPCNHVML SCCEGEEPLI VPEVRRPPEP EAAPRRVSEM EMASREALSL GTEAELPNSL PGDDQDECLM  **601** LPGELCQHLC INTVGSYRCA CFPGFELQGD GRTCRPDRGA PQLDTARESA PRSESAQVSP NTIPLPVPQP NTCKDNGPCR QVCRVVGDTA MCSCFPGYAI  **701** MADGVSCEDQ DECLMGTHDC SWKQFCVNTL GSFYCVNHTV LCAEGYILNA HRKCVDINEC VTDLHTCTRA EHCVNTPGSF QCYKALTCEP GYVLTDGECT  **801** DVDECVTGTH NCQAGFSCQN TKGSFYCQAR QRCMDGFLQD PEGNCVDINE CTSLLEPCRS GFSCINTVGS YTCQRNPLVC GRGYHANEEG SECVDVNECE  **901** TGVHRCGEGQ LCYNLPGSYR CDCKPGFQRD AFGRTCIDVN ECWVSPGRLC QHTCENTPGS YRCSCAAGFL LAADGKHCED VNECETRRCS QECANIYGSY  **1001** QCYCRQGYQL AEDGHTCTDI DECAQGAGIL CTFRCVNVPG SYQCACPEQG YTMMANGRSC KDLDECALGT HNCSEAETCH NIQGSFRCLR FDCPPNYVRV  **1101** SQTKCERTTC QDITECQTSP ARITHYQLNF QTGLLVPAHI FR**IGPAPAFA GDTISLTITK** GNEEGYFVTR R**LNAYTGVVS LQR**SVLEPRD FALDVEMKLW  **1201** RQGSVTTFLA KMYIFFTTFA P  **Start - End Observed Mr(expt) Mr(calc) ppm Miss Sequence**  **383 - 404 817.4257 2449.2552 2449.2601 -2 0 R.AQVSPSPETPEEIPQHPQLLPR.F**  ([Ions score 35](http://10.139.25.109/mascot/cgi/peptide_view.pl?file=../data/20120413/F007791.dat&query=47466&hit=1&index=FBLN2_MOUSE&px=1&section=5&ave_thresh=1&_ignoreionsscorebelow=20&report=0&_sigthreshold=0.05&_msresflags=1089&_msresflags2=2&percolate=-1&percolate_rt=0))  **1143 - 1160 886.9934 1771.9723 1771.9720 0 0 R.IGPAPAFAGDTISLTITK.G**  ([Ions score 66](http://10.139.25.109/mascot/cgi/peptide_view.pl?file=../data/20120413/F007791.dat&query=32308&hit=1&index=FBLN2_MOUSE&px=1&section=5&ave_thresh=1&_ignoreionsscorebelow=20&report=0&_sigthreshold=0.05&_msresflags=1089&_msresflags2=2&percolate=-1&percolate_rt=0))  **1172 - 1183 660.8659 1319.7162 1319.7197 -3 0 R.LNAYTGVVSLQR.S**  ([Ions score 48](http://10.139.25.109/mascot/cgi/peptide_view.pl?file=../data/20120413/F007791.dat&query=17509&hit=1&index=FBLN2_MOUSE&px=1&section=5&ave_thresh=1&_ignoreionsscorebelow=20&report=0&_sigthreshold=0.05&_msresflags=1089&_msresflags2=2&percolate=-1&percolate_rt=0))  25. [MMP14_MOUSE](http://10.139.25.109/mascot/cgi/protein_view.pl?file=../data/20120413/F007793.dat&hit=MMP14_MOUSE&db_idx=1&px=1&ave_thresh=1&_ignoreionsscorebelow=20&report=0&_sigthreshold=0.05&_msresflags=1089&_msresflags2=2&percolate=-1&percolate_rt=0)    **Mass:** 66162    **Score:** 171    **Matches:** 4(4)  **Sequences:** 4(4)  Matrix metalloproteinase-14  Sequence Coverage: **6%**; Matched peptides shown in **Bold Red**  **1** MSPAPRPSRS LLLPLLTLGT ALASLGWAQG SNFSPEAWLQ QYGYLPPGDL RTHTQRSPQS LSAAIAAMQK FYGLQVTGKA DLATMMAMRR PRCGVPDKFG  **101** TEIKANVRRK RYAIQGLKWQ HNEITFCIQN YTPKVGEYAT FEAIRKAFRV WESATPLRFR EVPYAYIREG HEKQADIMIL FAEGFHGDST PFDGEGGFLA  **201** HAYFPGPNIG GDTHFDSAEP WTVQNEDLNG NDIFLVAVHE LGHALGLEHS NDPSAIMAPF YQWMDTENFV LPDDDRR**GIQ QLYGSK**SGSP TKMPPQPRTT  **301** SRPSVPDKPK NPAYGPNICD GNFDTVAMLR GEMFVFKERW FWRVRNNQVM DGYPMPIGQF WR**GLPASINT AYER**KDGKFV FFKGDKHWVF DEASLEPGYP  **401** KHIKELGRGL PTDKIDAALF WMPNGKTYFF RGNKYYR**FNE EFR**AVDSEYP KNIK**VWEGIP ESPR**GSFMGS DEVFTYFYKG NKYWKFNNQK LKVEPGYPKS  **501** ALRDWMGCPS GGRPDEGTEE ETEVIIIEVD EEGSGAVSAA AVVLPVLLLL LVLAVGLAVF FFRRHGTPKR LLYCQRSLLD KV  **Start - End Observed Mr(expt) Mr(calc) ppm Miss Sequence**  **278 - 286 497.2719 992.5293 992.5291 0 0 R.GIQQLYGSK.S**  ([Ions score 43](http://10.139.25.109/mascot/cgi/peptide_view.pl?file=../data/20120413/F007793.dat&query=8759&hit=1&index=MMP14_MOUSE&px=1&section=5&ave_thresh=1&_ignoreionsscorebelow=20&report=0&_sigthreshold=0.05&_msresflags=1089&_msresflags2=2&percolate=-1&percolate_rt=0))  **363 - 374 646.3345 1290.6545 1290.6568 -2 0 R.GLPASINTAYER.K**  ([Ions score 59](http://10.139.25.109/mascot/cgi/peptide_view.pl?file=../data/20120413/F007793.dat&query=22577&hit=1&index=MMP14_MOUSE&px=1&section=5&ave_thresh=1&_ignoreionsscorebelow=20&report=0&_sigthreshold=0.05&_msresflags=1089&_msresflags2=2&percolate=-1&percolate_rt=0))  **438 - 443 421.1958 840.3770 840.3766 1 0 R.FNEEFR.A**  ([Ions score 31](http://10.139.25.109/mascot/cgi/peptide_view.pl?file=../data/20120413/F007793.dat&query=3186&hit=1&index=MMP14_MOUSE&px=1&section=5&ave_thresh=1&_ignoreionsscorebelow=20&report=0&_sigthreshold=0.05&_msresflags=1089&_msresflags2=2&percolate=-1&percolate_rt=0))  **455 - 464 585.3026 1168.5907 1168.5877 3 0 K.VWEGIPESPR.G**  ([Ions score 38](http://10.139.25.109/mascot/cgi/peptide_view.pl?file=../data/20120413/F007793.dat&query=16786&hit=1&index=MMP14_MOUSE&px=1&section=5&ave_thresh=1&_ignoreionsscorebelow=20&report=0&_sigthreshold=0.05&_msresflags=1089&_msresflags2=2&percolate=-1&percolate_rt=0)) |
| --- |
